# Defect Passivation Strategies in Halide Perovskite Solar Cells and LEDs

**DOI:** 10.1021/acsenergylett.5c04238

**Published:** 2026-03-19

**Authors:** Siddharth Singh, Arghya Sen, S. L. Aneesha, Woo Hyeon Jeong, Arup Ghorai, Junzhi Ye, Takuya Okamoto, Deepika Gaur, Subarna Samanta, Chun-Yun Wang, Sergio Gómez-Graña, Michael Saliba, Vishal Govind Rao, Pratik Sen, Robert L. Z. Hoye, Vasudevanpillai Biju, Lakshminarayana Polavarapu

**Affiliations:** † Department of Chemistry, 30077 Indian Institute of Technology Kanpur, Kanpur, Uttar Pradesh 208016, India; ‡ Graduate School of Environmental Science, 12810 Hokkaido University, Sapporo, Hokkaido 060-0810, Japan; § Inorganic Chemistry Laboratory, Department of Chemistry, 6396 University of Oxford, South Parks Road, Oxford, OX1 3QR, United Kingdom; ∥ Department of Materials Science and Engineering Pohang University of Science and Technology (POSTECH)77 Cheongam-ro, Nam-gu, Pohang 37673, Republic of Korea; ⊥ Research Institute for Electronic Science, Hokkaido University, Sapporo, Hokkaido 001-0020, Japan; △ CINBIO, Universidade de Vigo, Campus Universitario As Lagoas-Marcosende, Vigo, 36310, Spain; ▽ Institute for Photovoltaics (ipv), 9149 University of Stuttgart, Pfaffenwaldring 47, 70569 Stuttgart, Germany

## Abstract

Metal halide perovskites (MHPs) have rapidly emerged as a major focal point of modern materials research owing to their solution processability and attractive optoelectronic properties, including bright, efficient, and narrow luminescence. The rapid development of MHPs has largely been driven by their exceptional performance in solar cells, light-emitting diodes, and other optoelectronic devices. Although low-bandgap MHPs are defect-tolerant, their performance and stability in devices remain highly sensitive to surface and interfacial defects as well as grain boundaries. These imperfections hinder device performance by affecting charge-carrier transport within perovskite layers and across interfaces by introducing nonradiative recombination pathways. Consequently, the passivation of MHPs has emerged as a key driver in perovskite optoelectronics, advancing device efficiencies and long-term operational stability. This Review presents a state-of-the-art overview of defect types in bulk and nanocrystalline MHPs, and their effects on charge-carrier recombination. We also discuss passivation strategies and mechanisms, along with the passivator molecules that have been effective in enhancing the performance and stability of solar cells and LEDs. Ultimately, we highlight the outstanding challenges and future prospects.

## Graphical abstract

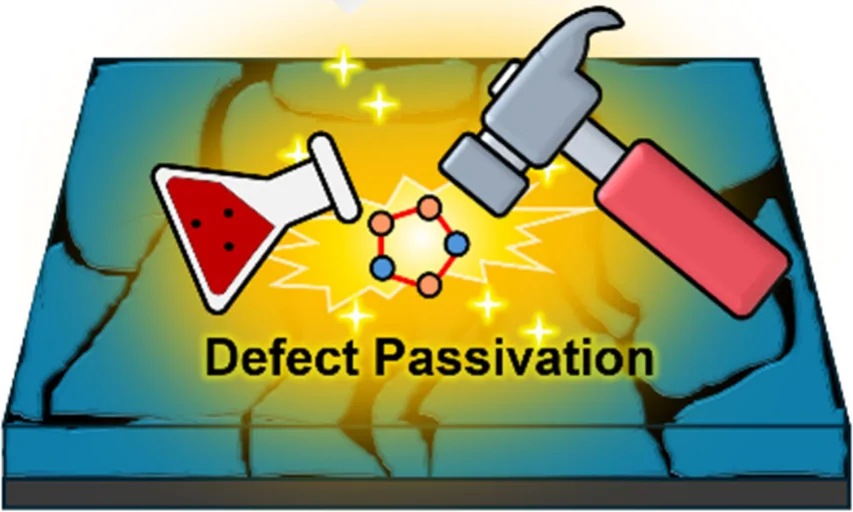

Metal halide perovskites (MHPs) have distinguished themselves as an emerging class of optoelectronic materials, achieving remarkable progress in devices, including photovoltaics, light-emitting diodes (LEDs), radiation detectors, and photocatalysis.^1–8^ They have taken the field by storm with their record-breaking efficiencies, with solar cell power conversion efficiency (PCE) advancing from ∼3.8% in 2009 to over 26% from 2023 onwards,^9–11^ and the external quantum efficiency (EQE) of LEDs evolving from below 1% in their earliest demonstrations to above 30%, all within almost one and a half decades.^12–14^ Although the remarkable photophysics of MHPs often take the spotlight, their impressive rise in device efficiency is equally driven by advances achieved in morphology control, compositional tuning, interface engineering, architectural design, and, above all, the effective management of defects (traps) and grain boundaries through passivation.^15–18^

Despite the defect-tolerant nature of iodide-based perovskites, among the broader family of metal-halide perovskites, structural heterogeneities, surface defects, and grain boundaries, which often occur during synthesis and crystallization, play a critical role in determining their performance in solar cells and LEDs.

Generally, defects (vacancies, dopants, or impurity-related) play a critical role in semiconductor devices, either beneficial or detrimental depending on their concentration and type. Ideally, semiconductors with the desired and controlled dopant concentration provide the required electronic properties, such as majority carrier type and their conductivity. However, if they form midgap states, their optoelectronic properties can be significantly affected. These properties include electrical conductivity, luminescence quantum yield (QY), carrier mobility, and carrier lifetime, and, in doing so, such defects would typically have a detrimental role on device performance. Defects often occur by thermodynamic and kinetic factors, as well as at interfaces and grain boundaries due to lattice termination, or because of the processing conditions used.^17,19–22^ Although defect formation may seem trivial in most materials, they critically affect the performance of semiconductor devices by impairing fundamental photophysical processes that hamper charge-carrier extraction and suppress radiative recombination through charge trapping. Although the low-bandgap iodide-based perovskites are known for their defect-tolerant nature, MHPs more generally [formula = ABX_3_, where A = monovalent cation (usually MA^+^, FA^+^, or Cs^+^), B = bivalent cation (usually Pb^2+^, Sn^2+^, or Ge^2+^), and X = Cl^–^, Br^–^, or I^–^] are not untouched by point defects and their detrimental consequences.^23^ For instance, solution-processed or vacuum-deposited MHP thin films typically exhibit grain boundaries and surface defects, whereas colloidal perovskite nanocrystals (NCs) tend to accumulate surface defects, resulting from the detachment of ligands and the concomitant removal of surface atoms during purification (Figure 1).^23,24^ Ultimately, these defects largely impact the hot carrier dynamics, charge-carrier transport, and radiative recombination, thus decreasing the corresponding device efficiency.^25–31^

**Figure 1:**
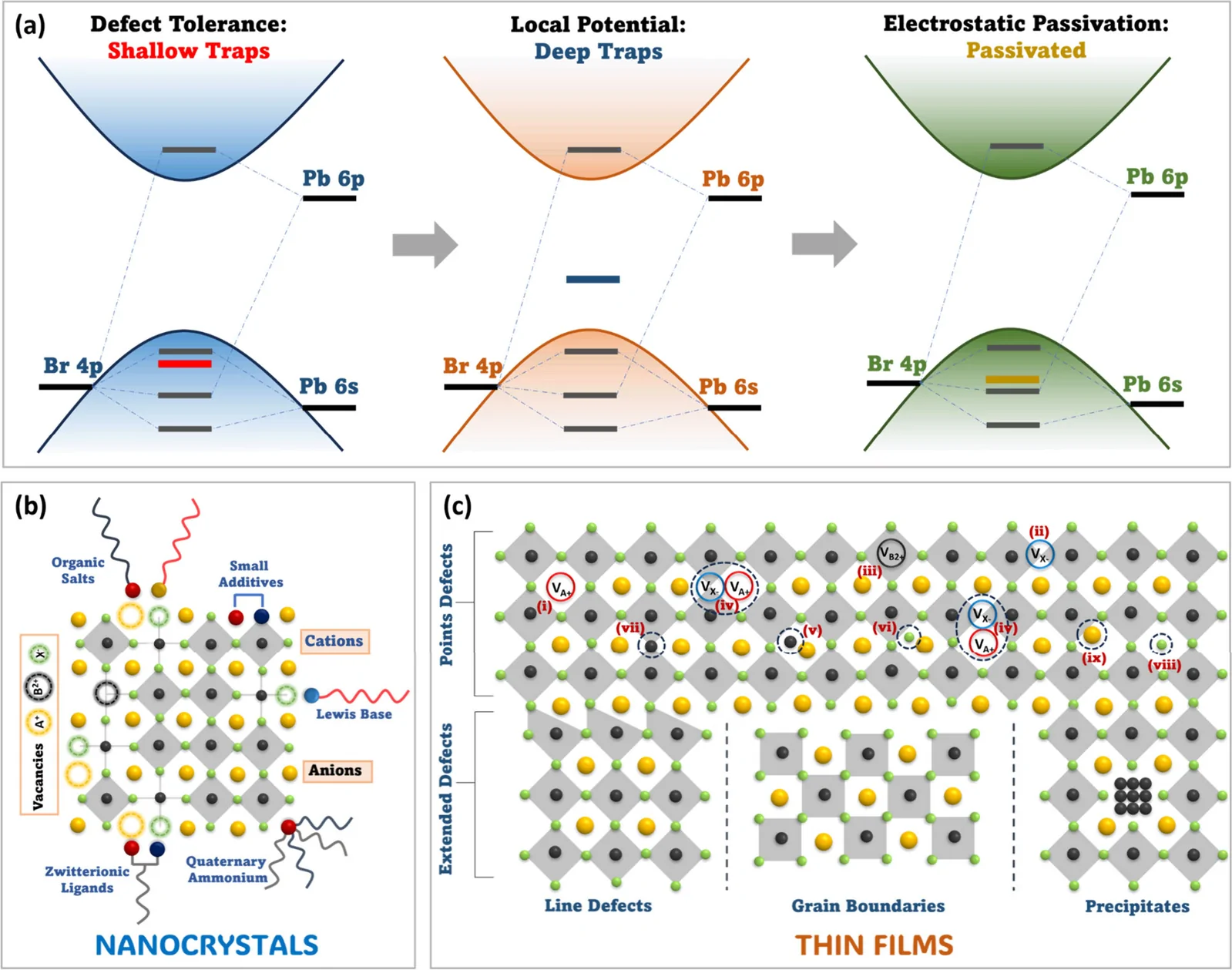
Defects in halide perovskites: (a) Mechanistic illustration of defect formation and mitigation: defect-tolerant MHPs (left), formation of midgap states induced by surface destabilization (middle), and recovery of defect tolerance through passivation (right).^47^ (b) Types of surface defects (A, B, and X vacancies) and different kinds of ligands and ionic species that can passivate the defects. (c) Type of potential point and extended defects in halide perovskite (ABX_3_) films. Identified point defects include: (i) A-site vacancy, (ii) halide (X) vacancy, (iii) B-site vacancy, (iv) Schottky defect, (v) B-site interstitial, (vi) X-site interstitial, (vii) B-site antisite, (viii) X-site antisite, and (ix) Frenkel defects.

Defect mitigation and efficiency enhancement in MHP-based optoelectronic devices critically rely on well-designed passivation strategies.^32,33^ Interestingly, one of the key features of MHPs is their remarkable ability to undergo effective defect passivation with the addition of organic molecules or inorganic ions. In materials chemistry, passivation refers to the process by which a material evolves from an “active state” into a stable, less reactive “passive state” by a thin protective layer.^34,35^ Primarily, the “active state” is characterized by midgap and near-band-edge states, which are suppressed through passivation, resulting in a cleaner bandgap in the “passive state”.^36,37^ This has been accomplished in MHPs mainly through two approaches: (1) chemical passivation, which addresses intrinsic defects by filling trap states within the perovskite lattice or its surfaces or the interface between perovskite and charge transport layers,^5,23,38^ and (2) physical passivation, which counters extrinsic degradation by protecting the material from external stressors such as UV radiation and moisture, while concurrently enhancing interfacial properties.^39–41^ For example, chemical passivation of an MHP layer with cyclohexylethylammonium iodide enhanced the absolute PCE by approximately 3%.^42^ In colloidal perovskite nanocrystals, chemical passivation increases the photoluminescence (PL) QY, in some cases to values approaching unity.^28,43^ The surface passivation is crucial for perovskite NCs because of weak ligand binding to their surfaces.^23^ Alternatively, a physical passivation method employing microencapsulation was adopted to prevent water penetration into the perovskite layer, resulting in a 95-fold increase in phase stability.^44^ These results highlight the importance of defect passivation for realizing high-performance perovskite solar cells and LEDs. Currently, passivation of MHPs is being extensively investigated, and over the years, a broad spectrum of passivators, including organic small molecules, polymers, and metal salts, has been reported for NC and bulk thin film MHPs.^5,45,46^

Accordingly, this review is aimed at providing state-of-the-art and perspective on the passivation of MHPs to improve the stability and efficiency of optoelectronic devices, especially solar cells and LEDs. We first discuss the current understanding of defects and passivation in MHPs and their potential impacts on photophysical properties, including PL and charge carrier relaxation. Further, the dedicated sections for photovoltaics and LEDs systematically categorize and discuss passivation strategies according to their interaction mechanisms, functional roles, and chemical nature, along with different kinds of passivators ranging from small molecules to polymers and metal ions to metal halides. Specifically, the discussion focuses on the role of passivating agents, emphasizing their influence on key device metrics, including the open-circuit voltage (*V*_oc_), fill factor (FF), PCE, luminance, and EQE.

## Types of Defects in MHPs and Their Effects on Devices

MHPs are soft materials due to their low M (metal)–X (halide) bond energies, dynamic lattices with strong ionic character, mobile ions, structural flexibility, and low defect formation energies. Therefore, they are not only easy to make but also easy to break. As a result, they are prone to defect formation and these defects can significantly influence their properties and device performance, despite their inherent defect tolerance. Mechanistically, Figure 1a illustrates the defect-tolerant nature of the MHPs, where nonbonding orbitals reside within the VB and do not generate midgap states. However, undercoordinated bromide ions at the surface experience a local destabilizing potential that shifts these states into the bandgap, rendering the defects electronically active. A subsequent passivation with appropriate ions or ligands restores the local electrostatic environment, driving the states back into the VB and thus suppressing defect activity.^23,47^

The types of defects in MHPs, in both colloidal NCs and thin films, are illustrated in Figure 1. Colloidal perovskite NCs are capped with long-chain ligands, typically in the form of ammonium cations or carboxylate anions, which noncovalently bind to the NC surface by occupying the A-site or X-site, respectively. After colloidal synthesis, it is essential to purify the NCs by washing them with antisolvents before using them in devices; otherwise, the resulting NC films remain sticky, and the excess ligands hinder charge transport. However, the washing process removes not only excess ligands but also some of the ligands that are bound to NC’s surface as well as surface atoms (A, B, and X), leading to surface defects (Figure 1b). This results in a significant increase in nonradiative charge-carrier recombination and a reduction in the PLQY. Interestingly, the PLQY can be recovered by passivation with organic molecules and metal halides, as illustrated in Figure 1b (more details about the effect of traps on charge carrier relaxation are discussed in the following section).

On the other hand, in bulk or thin film MHPs (ABX_3_), the intrinsic point defects include vacancies, interstitials, and antisite defects, where A and X vacancies generally lead to shallow states due to their low formation energies and thus are less detrimental. In contrast, interstitials or antisite defects can lead to the formation of deep traps, but they are less likely to form in MHPs due to their high formation energies.^48,49^ The point defects mainly arise during crystal growth and are influenced by chemical and environmental conditions. Moreover, in some instances, the point defects evolve into extended, higher-dimensional defects, such as line defects, pinholes, precipitates, and two-dimensional grain boundaries. This evolution is typically driven by defect migration and clustering during crystallization and subsequent stress such as from thermal energy, light or electrical bias, which induces local nonstoichiometry and lattice distortion, particularly at surfaces and grain boundaries.^50^ The extended defects are widely recognized for their detrimental impact on the performance of solar cells and LEDs.^17,51,52^ In addition to this, defect pairs also form in crystals, such as Frenkel defects, which consist of a vacancy and a corresponding interstitial involving the same ion, and Schottky defects, which involve a pair of vacancies with opposite charges.^17,36,53^

Figure 1c illustrates the common defects found in MHPs. These include point defects such as A-site vacancies (i), halide (X) vacancies (ii), B-site vacancies (iii), Schottky defects (iv), B-site interstitials (v), X-site interstitials (vi), B-site antisite defects (vii), X-site antisite defects (viii), and Frenkel defects (ix). In addition, extended defects are also present, including lattice dislocations (line defects), grain boundaries, and impurity substitution or precipitates and are inevitable in bulk thin films.

Importantly, the surface defects in MHPs, regardless of their form, are critical and have a profound impact on their optical and optoelectronic performance, as discussed in the following sections.

### Chemistry of Surface Defects

In this section, we discuss the types of defects that can form in halide perovskites and the factors that influence their concentration. Our discussion here is general, covering nanocrystals, thin films, and single crystals, as well as lead-halide perovskites and tin-halide perovskites. Later sections go into more specific details.

Surfaces, grain boundaries, and interfaces between two semiconductors (e.g., heterojunctions) constitute an abrupt change to the lattice of a material and can give rise to substantially different defect chemistry than the bulk. It has been widely found that there is a higher density of traps at surfaces and grain boundaries of perovskites, which can arise from under-coordinated ions or dangling bonds that introduce deep trap states.^54^ For example, the surface recombination velocity (SRV) of methylammonium lead bromide (MAPbBr_3_) single crystals was found to be 3.4 ± 0.1 × 10^3^ cm s^–1^.^55^ Although this SRV value is orders of magnitude lower than those of traditional defect-sensitive semiconductors (including Si and GaAs), the SRV of MAPbBr_3_ single crystals could still be improved by an order of magnitude through passivation.^55,56^

The types of surface defects that form are influenced by whether the surface termination is PbI_2_ or a salt of the A-site cation (e.g., methylammonium iodide).^56–59^ A computational investigation into MAPbI_3_ by Ambrosio et al. illustrated that surfaces fully terminated with PbI_2_ or MAI led to the spatial separation of electron and hole polarons, whereas the partial coverage of the surface with MAI resulted in the polarons being closer together and more prone to nonradiative recombination,^59^ especially for under-coordinated lead cations. Furthermore, the authors found that the MAI-terminated surface was more defect-tolerant than the PbI_2_-terminated surface, which is susceptible to the formation of deep lead and iodide vacancies.^59^ Other computational and experimental works have otherwise suggested that PbI_2_ termination on surfaces is beneficial for passivation in both thin films and nanocrystals.^29,60,61^ That is, PbI_2_-excess perovskites were found to exhibit higher short-circuit current densities in devices, and this was attributed to avoiding grain boundaries that were enriched with organic species.^61,62^ Furthermore, there are contradictory results in the literature on whether having excess PbI_2_ is beneficial for photovoltaic devices, with some reports claiming that PbI_2_-rich surfaces lowers the formation energy of I_i_^–^/V_I_^+^ Frenkel pairs,^61,63^ while others report excess PbI_2_ to suppress nonradiative recombination.^29^ Certainly, excess-PbI_2_ perovskites are widely used in efficient perovskite photovoltaics.^64–66^

In addition, computational studies suggest that whether the perovskite surface is PbI_2_- or MAI-terminated determines the band positions of the surface, and therefore the energy alignment with the charge transport layer, thus affecting charge-carrier extraction or injection.^67^ The surface termination can also influence the likelihood of metallic lead (Pb^0^) formation, which is widely considered to be detrimental and cause increased nonradiative recombination.^61,68,69^ It has been proposed that this can take place more readily on PbI_2_-rich surfaces, where iodide vacancies and iodide interstitials (a Frenkel pair) are thermodynamically favored to form.^63^ This is proposed to lead to the formation of I_2_ on the surface, which accompanies the formation of Pb^0^.^61^ Thus, adding excess methylammonium halide can suppress the generation of these species and avoid the introduction of recombination centers due to Pb^0^.^68,69^

The energetics of the surface also lead to different behaviors between the surface and bulk in tin-based perovskites. Ricciarelli et al. found through computations on MASnI_3_ that Sn^2+^ is more stable than Sn^4+^ in the bulk, provided that external oxidizing agents are absent. This occurs due to the high ionization potential of MASnI_3_, such that the defect transition levels appear within the valence band. By contrast, at the surface, Sn^4+^ can be stabilized due to Fermi level pinning, especially on SnI_2_-rich surfaces. This Sn^4+^ can act as a deep electron trap, and when reduced to Sn^2+^, will release two holes that lead to the *p*-type self-doping typically found in tin-based perovskites. Achieving increased MAI coverage on the surface mitigates these effects, and it was found that the transition between Sn^2+^ and Sn^4+^ shifted closer to the valence band, making Sn^4+^ formation less likely.^70^

The processing of the perovskites can also affect the defect chemistry of the surface. As discussed above, the purification of all-inorganic perovskite nanocrystals using polar antisolvents can result in the removal and etching of halides from the surface.^71,72^ If the antisolvents are protic, they can cause the removal of X-type ligands (commonly oleate and oleylammonium) through proton transfer, such that these ligands are no longer charged and are unable to bind to the perovskite surface. In addition, antisolvents of high relative polarity (e.g., butanol or acetone) can also cause the removal of oleate and oleylammonium ligands through amide condensation reactions. That is, antisolvents of high relative polarity are more likely to shift the equilibrium of these X-type ligands toward their neutral carboxylic acid and amine form, which then react together through condensation reactions to form amides that would not bind to the perovskite surface. This was substantiated by ^1^H NMR measurements, which showed an increased concentration of amides and water in the supernatant when using more polar antisolvents. The removal of these ligands can also cause the removal of surface halides hydrogen bonded to the oleylammonium ligand, and iodide is especially prone to removal, since this is more weakly bound in the perovskite lattice than bromide. Halide removal and the introduction of vacancies at the perovskite surface not only lowered PLQY, but also exacerbated halide segregation when used in LEDs.^71^

Structural heterogeneities in MHP thin films, such as grain boundaries and local lattice distortions, are closely linked to the formation and redistribution of point defects. Extended defects can locally alter coordination environments and chemical potentials, lowering the formation energy of vacancies, interstitials, or antisites. Conversely, the accumulation and reconstruction of point defects can further distort the lattice, leading to defect complexes that deviate from the ideal perovskite structure.^50,73^ Recent advances in high-resolution electron microscopy techniques, which avoid damaging the delicate perovskite specimens, have enabled new insights into how the nanoscale structure influences optoelectronic properties.^74^ For example, Uller-Rothmann et al. obtained high-resolution transmission electron microscopy images of FAPbI_3_ and MAPbI_3_, and directly observed point defects occurring in these materials, verifying calculations. These authors also found many grain boundaries to be highly pristine, helping to explain why polycrystalline thin films can perform efficiently. At the same time, they spotted the presence of amorphous material and the generation of point defects at some grain boundaries.^75^ Furthermore, Doherty et al. used photoemission electron microscopy (PEEM) to identify the existence of clusters of deep traps in triple-cation perovskites, forming almost exclusively at grain boundaries.^76^

### Effects of Defects

Defects are widely found in the bulk, surfaces, and interfaces of semiconductors, where they are almost ubiquitously thermodynamically favored to form.^77^ Such defects span over a wide range of different types, from atomic-level point defects (vacancies, interstitials, and antisites) through to macroscopic-level structural defects and voids.^5,77–79^ These defects have many detrimental effects on the performance of semiconductors, such as by (i) reducing the PLQY, quasi-Fermi level splitting and *V*_OC_ in devices by acting as recombination centers that increase the rate of nonradiative recombination,^55^ (ii) scattering charge-carriers and lowering mobility, (iii) localizing charge-carriers, such that they can only move by hopping between adjacent defects,^59^ (iv) acting as sites of extrinsic self-trapping, which can introduce further loss channels,^78^ or (v) enabling photoinduced phase-separation through halide migration.^61,80^ Beyond these detrimental effects on charge-carriers at the band-edge (or ‘cold’ carriers), point defects can also influence the cooling of hot carriers (HCs). HCs are generated through high injection or following the absorption of photons with energies above the bandgap. Typically, the excess energy is lost as heat through the dissipation of phonons when the HCs cool to the band-edge. But if this process could be sufficiently slowed down to allow the HCs to be extracted, then solar cells with PCEs exceeding the Shockley-Queisser (SQ) limit could be achieved.^81–83^ Defects influence the HC cooling process by modifying the degeneracy and energy gap between states in the bands,^26^ which affects their cooling rate. Furthermore, HCs with greater excess energy have a higher thermodynamic driving force to relax into traps, turning shallow traps from benign states for cold carriers to harmful states for hot carriers,^84^ thus leading to reductions in PLQY. Mitigating the effects of these traps on HC cooling is important, since many recent applications of perovskites involve illumination with high-energy photons (UV-backlit LEDs, PVs absorbing UV/blue light) or high-injection operating conditions (LEDs and lasers).^84^ A key enabling property of lead-halide perovskites is their defect tolerance. This is widely regarded as the ability of point defects to not be detrimental to the nonradiative recombination rate or mobility of free charge-carriers.^85,86^ Defect tolerance for free charge-carriers occurs when the most common point defects form shallow traps, which, according to the one-trap Shockley-Read-Hall model, can allow a low nonradiative recombination rate to be preserved despite a high trap density.^86–88^ For example, in MAPbI_3_, several computational investigations have demonstrated that the lowest formation energy (and therefore most common) point defects are iodide vacancies (V_I_), methylammonium vacancies (MA_V_), lead interstitials (Pb_i_) and methylammonium interstitials (MA_i_).^37,63,84,89^ As a result, high PV efficiencies and PLQYs can be achieved despite high densities of point defects on the order of 1 × 10^16^ – 1 × 10^17^ cm^–3^. However, the defect tolerance of MAPbI_3_ is not generalized over the entire family of lead-halide perovskites. In changing the halide changed from iodide to lighter halides, the ionicity of the lead-halide bond increases, leading to an enhancement of the bandgap. The lattice parameter of the perovskite also decreases. Both factors lead to halide vacancies changing from being shallow in iodide-based perovskites to deep in chloride-based perovskites, as shown computationally and experimentally.^90–92^ Furthermore, even in the iodide-based perovskites, there have been conflicting reports on the shallow vs deep nature of traps from computational investigations. For example, some groups have found iodide interstitials and lead vacancies to be shallow, while others believe these to be low-concentration deep traps.^37,89,93,94^ Experimental measurements of iodide-based perovskites using deep level transient spectroscopy (DLTS) have indeed revealed deep traps to be present, with trap energies of 0.76 eV and relatively high concentrations of 9.5 × 10^14^ cm^–3^. 18%-efficient PV devices were still achievable because of the low capture cross sections of these defects (10^–14^ cm^2^),^95^ which may be enabled by the high dielectric constants and low effective mass of the perovskites.^86,87^ However, it is clear that approaching the radiative limit in efficiency requires strategies to suppress or passivate defects in bulk and interfaces in perovskite optoelectronics.^33,96,97^ In addition, the extent to which shallow traps in halide perovskites influence the HC cooling rate is unknown, but it is clear that passivating traps is beneficial for slowing down HC cooling.^25,84^

## Effects of Passivation on the Charge-Carrier Kinetics of MHPs

To fully assess the role and effectiveness of surface passivation on the performance of MHPs, it is important to understand changes in the nonradiative recombination process for cold carriers and hot carriers (Figure 2). In this section, we discuss the mechanism of defect-induced nonradiative recombination and how the suppression of interface recombination could be quantified. We discuss the role of passivation in the kinetics of both cold carriers and hot carriers.

**Figure 2:**
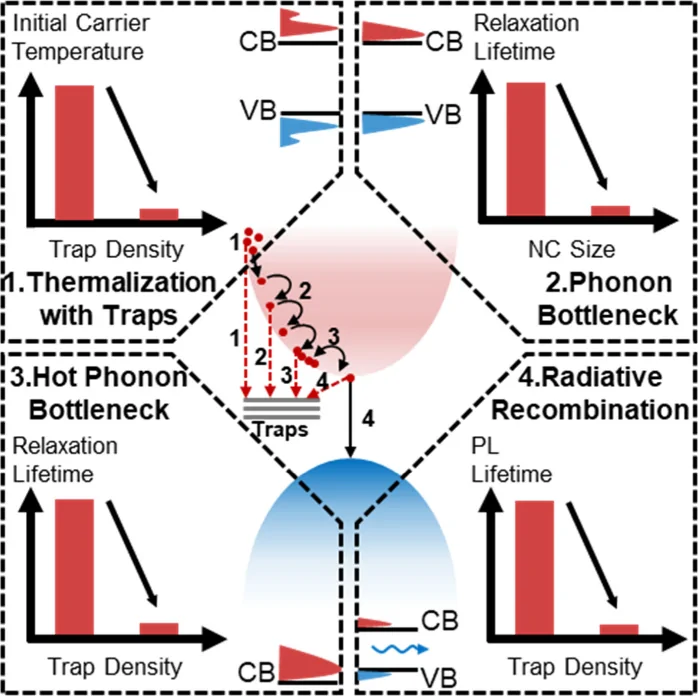
Effect of traps on hot carrier (HC) relaxation and recombination kinetics. Schematic summary highlights trap-mediated HC losses: following photoexcitation, hot carriers undergo thermalization via carrier–carrier scattering (Process 1), relaxation toward the band-edge via carrier–phonon scattering and phonon bottleneck effects (Process 2), trap-assisted HC capture that bypasses the hot phonon bottleneck and accelerates cooling (Process 3), and band-edge radiative and nonradiative recombination, which can be influenced by trap densities (Process 4).

### Suppression of Nonradiative Recombination and Enhancement in Cold-Carrier Lifetime

The types of recombination processes that take place depend on whether the material is dominated by free charge-carriers or excitons. In the case of free carriers, three major types of recombination take place: trap-assisted (nonradiative), radiative, and Auger. Trap-assisted recombination takes place when a defect state within the bandgap captures one charge-carrier first, before capturing the opposite charge-carriers, causing a loss of energy as heat and a reduction in the free carrier population. Radiative recombination occurs when photogenerated electrons and holes at the conduction- and valence-band edges recombine and emit photons with energies corresponding to the electronic transition, typically the bandgap. Auger recombination occurs when a photogenerated electron recombines with a hole, followed by energy and momentum transfer to another electron or hole, making this a three-body process.^98^ The simplest governing equation for the excess concentration of free carriers after photoexcitation (i.e., extra concentration of charge-carriers under illumination than in the dark; Δ*n*) is given in eq 1.^5,99^

1 $$\frac{d\Delta n}{dt}=G\left(t\right)-k_{1}\Delta n\left(t\right)-k_{2}\Delta n{\left(t\right)}^{2}-k_{3}\Delta n{\left(t\right)}^{3}=G\left(t\right)-\Delta n\left(t\right)R\left(t\right)$$

In eq 1, *G*(*t*) is the generation rate, which is constant if under 1-sun illumination, while *k*_1_, *k*_2_ and *k*_3_ are the rate constants for trap-assisted, radiative and Auger recombination, respectively, and *R*(*t*) is the overall recombination rate.

Under open-circuit conditions, then $\frac{d\Delta n}{dt}=0$ , and so *G*(*t*) = *R*(*t*). From this, it can be seen that if there is a higher nonradiative recombination rate constant, *k*_1_, due to the role of defects, then the excess carrier density will be lower, resulting in a lower quasi-Fermi level splitting and lower *V*_OC_. However, the role of defects is not simply uniform within the bulk but also at surfaces. To account for this, we can consider the excess carrier density not only as a function of time, but also as a function of distance away from the surface which is illuminated, *x*, i.e., *G*(*x*, *t*) and *R*(*x*, *t*). Modeling a flat slab that is uniformly illuminated on its surface, we can apply Fick’s second law:

2 $$\frac{\partial \Delta n\left(x,t\right)}{\partial t}=G\left(x,t\right)-n\left(x,t\right)R+D\frac{\partial^{2}\Delta n\left(x,t\right)}{\partial x^{2}}$$

In eq 2, *D* is the diffusion coefficient of charge carriers, which depends on their mobility. Nonradiative recombination at the surfaces can then be modeled as a surface recombination velocity, *S*, which can be applied as a boundary condition. For example, if *S* is the same at the front and rear surfaces, then eq 3 applies.

3 $${D\frac{\partial^{2}\Delta n\left(x,t\right)}{\partial x^{2}}|}_{x=0,d}=S\Delta n\left(x,t\right)$$

The recombination rate constants, *S* and *D*, can all be obtained by fitting time-resolved PLdecay curves, measured at different fluences from the sample.^100,101^ From these parameters, the effective charge-carrier lifetime can be obtained from eq 4.

4 $$\tau_{\mathrm{eff}}=\frac{\overset{®}{\Delta n\left(x,t\right)}}{\overset{®}{\Delta n\left(x,t\right)}\mathrm{R̅}}$$

In eq 4, all parameters are averaged over time and through the depth of the sample. This effective charge-carrier lifetime can be split into its different components, as shown in eq 5.

5 $$\frac{1}{\tau_{\mathrm{eff}}}=\frac{1}{\tau_{n,r}}+\frac{1}{\tau_{\mathrm{rad}}}+\frac{1}{\tau_{\mathrm{Auger}}}+\frac{1}{\tau_{\mathrm{surf}}}$$

Each lifetime in eq 5 is inversely proportional to the rate constants, while the surface lifetime is inversely proportional to *S*. Thus, minimizing nonradiative recombination and surface recombination velocity are critical.

Charged surface defects and other defects, such as Pb vacancies, Pb metal interstitials, do contribute toward deep trap defects, which cause large *k*_1_. Numerous studies have shown that passivation strategies successfully minimize these nonradiative loss processes. Specifically, ionic, molecular, and structural passivation approaches have been demonstrated to substantially reduce the surface recombination velocity (SRV). Oliver et al. reported that the combination of ionic additive passivation and interfacial LiF treatment reduced the SRV from over 5000 cm/s to below 500 cm/s. This synergistic approach suppressed both bulk and interfacial losses, leading to significant improvements in PLQY, carrier lifetime, and overall device performance.^102^ Building upon this, Pan et al. introduced a chemical surface reconstruction strategy for Sn/Pb alloy perovskites using 1,4-butanediamine (BDA) and ethylenediammonium diiodide (EDAI_2_). This treatment reduced Sn^4+^-related surface traps and passivated A-site and halide vacancies, resulting in enhanced carrier lifetimes, increased PLQY, and improved device efficiency in all-perovskite tandem solar cells.^103^ In a similar vein, chemical reconstruction at Pb-based MHP interfaces can be achieved by forming well-defined 2D/3D heterojunctions using alkyl amine ligands. Azmi et al. demonstrated that incorporating an alkyl amine ligand enables near-phase-pure 2D perovskite formation at both the top and the buried bottom interfaces of 3D perovskite absorbers, which mitigates interfacial recombination, ion migration, and electric-field inhomogeneities. Collectively, these double side 2D/3D heterojunctions reduce interfacial trap density and suppress nonradiative recombination in MHP films, leading to higher *V*_OC_ and improved device efficiency and operational stability.^104^ Interfacial engineering has also been shown to play a pivotal role in extending carrier lifetimes. Zhu et al. demonstrated that hole-selective contact molecules of donor–acceptor type can effectively passivate interfacial traps and suppress nonradiative recombination at the perovskite/charge transporting layer (CTL) interface, while also enabling efficient hole extraction in tandem perovskite solar cells (PSCs).^105^ Additionally, deQuilettes et al. revealed that chemical surface post-treatment of 3D perovskites with hexylammonium bromide (HABr) enables the simultaneous formation of an iodide-rich 2D surface layer and a bromide gradient extending into the bulk. This results in a tunable 2D/3D heterostructure that induces built-in surface electric fields through band bending and bandgap grading. This approach facilitates efficient charge separation and extends carrier lifetime by suppressing nonradiative recombination.^106^

Finally, if we go toward a system dominated by excitons rather than free-carriers (i.e., a material with high exciton binding energy), eq 1 is modified to eq 6.^107^

6 $$\frac{dn_{\mathrm{ex}}}{dt}=G\left(t\right)-k_{1}n_{\mathrm{ex}}\left(t\right)-k_{2}n_{\mathrm{ex}}\left(t\right)-\frac{1}{2}k_{3}n_{\mathrm{ex}}{\left(t\right)}^{2}$$

In eq 6, *n*_ex_ is the population of excitons, *k*_1_ is the trap-mediate recombination rate constant, *k*_2_ is the radiative rate constant, and *k*_3_ is the exciton–exciton annihilation rate constant. The orders are different in this case because excitons are electron–hole pairs that behave as a quasi-particle. Nonradiative recombination involves the entire exciton being captured by a trap state and is still a first order process. For radiative recombination, this changes from second to first order since both electrons and holes behave as one unit. For exciton–exciton annihilation, this involves two excitons coming together, and so is a second-order process.^108^

These different processes for excitons could be determined through a combination of time-resolved PL, transient absorption spectroscopy, and PLQY measurements. For example, Ye et al. passivated α-CsPbI_3_ perovskite nanoplatelets with PbI_2_ coordinated with oleate and oleylammonium ligands, which repaired damaged PbI_6_ octahedra at the surface. This led to an improvement in the PLQY from 48 ± 2% to 65 ± 1% at 0.3 mW cm^–2^ power density (405 nm continuous wave excitation laser). In transient absorption spectroscopy measurements, an initial fast decrease in the population of cold-carriers was observed, followed by a slower decay. The initial fast decay was attributed to exciton–exciton annihilation since this is a higher-order process than monomolecular recombination. For time-resolved PL measurements at lower fluences, the higher-order exciton–exciton annihilation process is negligible, and the kinetics could be entirely attributed to trap-dominated and radiative recombination. Thus, comparing these measurements allowed the rate constants in eq 6 to be obtained. It was found that passivation led to *k*_3_ constant decreasing from 0.382 cm^2^ s^–1^ to 0.282 cm^2^ s^–1^, while the trap-dominated recombination rate constant also decreased from 0.009 ns^–1^ to 0.002 ns^–1^.^108^

### Influence of Defects and Passivation on Hot Carrier Cooling Dynamics

Modulating hot carrier (HC) thermalization and relaxation in halide perovskites is important after photoexcitation to allow more carriers to cool down to the band-edge, which offers opportunities to develop more efficient optoelectronic devices.^109,110^ Although collecting HCs offer the potential to exceed the SQ limit, their excess energy is typically lost within picoseconds due to rapid hot carrier–carrier interactions (thermalization), hot carrier–phonon scattering (relaxation) and more recently hot carrier-defect interactions (hot carrier trapping) (Figure 2).^84^ Interestingly, lead halide perovskites exhibit relatively slow HC cooling, attributed to low optical phonon energies and hot phonon bottleneck effects. However, defects introduce alternative energy loss pathways during the thermalization and relaxation processes that bypass this bottleneck, accelerating HC cooling. First, the thermalization process refers to hot carriers at nonthermal equilibrium states after photoexcitation (Figure 2, Process 1). The energy loss during this process primarily comes from carrier–carrier scattering at subpicosecond time scale.^107,111^ Commonly used Maxwell–Boltzmann distribution function (eq 7) is not suitable for analyzing this process as the equation only valid by approximating HCs follow the Fermi–Dirac distribution^112,113^

$$-\frac{\Delta T}{T}\left(E\right)\propto e^{-\left(E-E_{f}\right)/k_{B}T_{c}}$$ 7

where $\frac{\Delta T}{T}\left(E\right)$ is the transient transmittance obtained from the TA measurement, *T*_c_ is the carrier temperature. The influence of defects on thermalization can be visualized by comparing the initial carrier temperature fitted from eq 7 when the HCs just reach equilibrium conditions. The NCs with higher defects tend to have lower initial hot carrier temperature, typically at around 0.3–0.5 ps.^25^ For example, Ye et al. fabricated CsPbBr_3_ NCs with different surface defects by intentionally removing different amount of ligands and halides from NC surface, the higher defect NCs show much lower initial hot carrier temperature than the lower defect NCs.^25^ This defect-induced thermalization loss can be mitigated by effective surface passivation. Dai et al. increase the initial carrier temperature for lead–tin halide perovskite NCs from 5018 to 9742 K via Na passivation of the NC surface.^111^ The presence of excess Na on the NC surface blocks the loss channel and decouples the HCs from defects during the thermalization process.

Passivation suppresses the loss pathways of carriers, reduces non-radiative recombination, preserves the hot phonon bottleneck, extends carrier thermalization times, and minimizes energy loss through trap-mediated scattering.

Hot carrier relaxation process allows HCs at thermal equilibrium to exchange energy with their surroundings to cool down to band-edge including lattices (carrier-phonon) and adjacent HCs (hot phonon bottleneck or Auger reheating). The intrinsic phonon bottleneck is governed by NC composition,^114,115^ i.e., phonon energy, and the size of the NCs (Figure 2, Process 2).^113,116^ The A-site or B-site cation doping can modulate the perovskite Pb-halide octahedral cage, which is vital for lattice tilting and alternatively influences the cooling kinetics. Additionally, the size of the NCs determines the energy offsets between the high-energy and low-energy states. For example, for tin-based perovskite NCs, the smaller NC size can lead to larger and more discrete energy offsets, so that the relaxation time from higher energy states to lower energy states increases.^113,117^ It is noted that the phonon bottleneck is independent of excitation carrier density, and it is governed by intrinsic material properties. However, the change in composition and size often alternates surface conditions. For instance, the smaller dots tend to have higher surface defects due to larger surface-to-volume ratio and faster nucleation and growth process;^5^ the hybrid organic–inorganic A-site NCs tend to have less surface ligand loss due to stronger halide-ligand binding.^118,119^ Decoupling the effect of surface defects from the intrinsic cooling process needs to be carefully studied when performing spectroscopy analysis of NC relaxation.

Trap-induced HC loss significantly influences the hot carrier population density during the relaxation process (Figure 2, Process 3), as HCs can be directly trapped into the defects.^25,84,120^ The effect of hot phonon bottleneck and Auger reheating relies on the carrier density reaching over 1 × 10^18^ cm^–3^. As the trap states offer a new pathway, the carrier density would be significantly lower in the NCs with high defects than those NCs with low defects under the same laser excitation fluence. As a result, the HC relaxation time would be reduced due to the absence of hot phonon bottleneck effect and Auger reheating.^25,120^ Mondal et al. established a quantitative hot carrier trapping model to illustrate the various HC relaxation routes, particularly carrier–trap and carrier–phonon interactions,^25^ a simple kinetic model was developed based on eq 8:

8 $$\frac{dn_{\mathrm{hot}}}{dt}=Ĩ\left(t\right)n_{\mathrm{cold}}-\alpha n_{\mathrm{cold}}n_{\mathrm{hot}}-\phi n_{\mathrm{ph}}n_{\mathrm{hot}}-\gamma \left(N_{t}-n_{t}^{*}\right)n_{\mathrm{hot}}$$

where *Ĩ* represents the push Gaussian envelope. *n*_hot_, *n*_cold_, *n*_ph_, *N*_t_ and *n*_t_^*^ are the densities of hot and cold carriers, vacant phonons, and total and occupied traps, respectively. The coefficients α and ϕ represent the hot–cold carrier and carrier–phonon scattering, respectively, and are based on values extracted from our previous work,^116,121,122^ while γ denotes the propensity for these defects to act as traps for HCs. It shows that the cooling time depends on the NC bandgaps, excitation fluence, and trap energy position or the coupling strength between HCs and trap states.

These defect-induced cooling losses can be mitigated through surface passivation, which reduces the coupling between HCs and localized states. Such strategies preserve the hot phonon bottleneck and extend cooling lifetimes, maintaining elevated carrier temperatures longer. Thus, defect passivation not only improves steady-state carrier dynamics but also emerges as a critical factor in realizing HC-based optoelectronic devices. One notable example is the use of dual-passivation ligands targeting both the bulk and the interface. A bulk passivator with an electron-withdrawing core, namely, a diammonium-based molecule, was incorporated to suppress both shallow and deep traps. This dual passivation improved PL lifetimes and device efficiency while delaying hot carrier relaxation, as evidenced by transient absorption measurements showing extended bleaching signals and elevated carrier temperatures.^123^ In addition to ligand and interfacial treatments, recent efforts have focused on engineering electron transport materials (ETMs) to further influence hot carrier dynamics through both passivation and energetic alignment. A representative example is the rational design of nonfullerene ETMs that combine strong defect passivation with favorable band alignment. For instance, a cyano-functionalized bithiophene imide dimer (CNI2)-based polymer (PCNI2-BTI) forms strong Lewis acid–base interactions with undercoordinated Pb^2+^ ions and adopts a face-on π-π stacking geometry at the interface. This configuration reduces the interfacial trap density and improves carrier extraction. Transient absorption spectroscopy revealed more efficient hot electron injections, suppressing trap-mediated recombination and decelerating hot carrier cooling.^124^ Complementary to these approaches, fullerene-based ETM strategies have also been explored. Gong et al. demonstrated that the modification of [6,6]-phenyl-C_61_-butyric acid methyl ester (PCBM) with tetramethylthiuram disulfide (TMDS) under UV activation led to n-type doping and enhanced electron mobility. This modification also facilitated strong Pb^2+^ coordination and smoother interface contact, significantly suppressing defect-mediated fast cooling and enabling prolonged carrier relaxation times. Time-resolved PL and differential lifetime analysis confirmed that TMDS treatment results in higher carrier temperature retention over extended periods.^125^ Apart from passivation, different ligand shells also influence the relaxation process of NCs. Damping ligands such as polysiloxane shell around CsPbBr_3_ NCs would increase the relaxation time as it alters the electron–phonon coupling.^81^

In summary, defect passivation strategies not only suppress the loss pathway of the HCs but also reduce nonradiative recombination at the band edge in halide perovskite NCs. By reducing trap densities, passivation preserves the hot phonon bottleneck, extends carrier thermalization times, and minimizes energy loss through trap-mediated scattering. Various approaches, including molecular surface passivation and interfacial engineering with tailored electron transport layers, have demonstrated the ability to slow the hot carrier relaxation and enhance high-temperature carrier retention. These strategies collectively highlight that controlling defects is essential not only for improving steady-state photovoltaic performance but also for realizing the next generation of hot-carrier-based optoelectronic devices. Continued research integrating passivation strategies with structural engineering will be the key to fully unlocking the potential of slow hot carrier cooling in perovskite systems.

## Defects in MHP Solar Cells and Their Passivation

### How Defect Passivation Affects *J*_sc_, *V*_oc_, FF, and PCE

The overall PCE of a solar cell, under one sun illumination of 100 mW/cm^2^, is governed by the product of the short-circuit current density (*J*_sc_), open circuit voltage (*V*_oc_), and fill factor (FF). The performance of PSCs is influenced by the defects in the bulk of the perovskite, at grain boundaries (GBs), the surface of the thin-films, and interfacial contact layers, as discussed in the above sections.^36^ Such defects can act as nonradiative recombination centers, provoking a reduction in charge carrier lifetime and overall device efficiency. For instance, Sherkar et al.^126^ studied the influence of GB- and interfaces on the charge carrier recombination, and found that trap-assisted recombination at interfaces is the dominant loss mechanism of PSCs efficiency. Given its substantial impact on device behavior, defect passivation is considered a key strategy for improving the stability and efficiency of PSCs.^80,127^ It is worth noting that one of the highest reported PCEs, with 25.8% (certified 25.5%) with improved stability, has also been achieved via an interfacial passivation approach.^128^ For further development of high-performance perovskite solar cells, it is essential to understand the underlying mechanism of defect passivation effects on the key performance parameters, including *J*_sc_, *V*_oc_, and FF.

In general, *J*_sc_ is mainly governed by the optical absorption of the active medium (Perovskite) and the efficiency with which the photogenerated charge carriers are extracted.^129^ To achieve a high *J*_sc_, considerable efforts have been devoted to optimizing the bandgap, improving the light-harvesting efficiency, e.g. by depositing a sufficiently thick active material, as well as enhancing the charge collection capability of PSCs. The defect passivation at the surfaces and GBs helps produce highly compact films, increase the charge carrier diffusion length, and suppress the defect traps states that would cause nonradiative recombination, further enhancing the charge extraction efficiency and thus *J*_sc_ of the devices.^127^ For example, Hu et al.^130^ strategically selected ethylenediammonium diiodide (EDAI_2_) and glycine hydrochloride (GlyHCl) to passivate the top and bottom Sn–Pb mixture perovskite layer, respectively. The surface treatment with EDAI_2_ leads to lower carrier trap densities, while GlyHCl treatment helps to passivate recombination centers at the bottom of the perovskite layer and increase the crystallinity of perovskite film. In addition, EDA^2+^ and GlyH^+^ cations induce surface dipoles, which facilitate electron and hole transfer to the charge collection layers, respectively. The overall bifacial passivation leads to an increase of *J*_sc_ from 31.6 to 32.5 mA cm^–2^ with a *V*_oc_ increasing from 0.79 to 0.89 V, resulting in the PCE to 23.6%, which is the record PCE for Sn–Pb mixture PSCs. Similarly, Tang et al.^131^ adopted trometamol additive to passivate both bulk and surface defects of MAPbI_3_ film. Trometamol molecule effectively reduces ionic defects and enhances the grain size of the MAPbI_3_ film through Pb^2+^/–NH_2_ coordination bonds and I^–^/–OH hydrogen bonds. As a result of the dual passivation approach, *J*_sc_ is increased from 20.59 to 21.98 mA cm^–2^. Note that the *J*_sc_ enhancement via defect passivation requires very careful selection of passivators and controlling the concentration of passivators in order to enhance both film quality and charge extraction kinetics. Furthermore, the interfacial passivation by using chemical additives or inserting a 2D layer can facilitate the charge extraction, reducing the energy mismatch between the perovskite and charge transport layer.^132,133^ Li et al.^134^ incorporated a small amount of 2-aminoindan hydrochloride additive into the precursor inks to manipulate the crystallization of methylammonium-free perovskite (Cs_0.05_FA_0.95_PbI_3_), leading to an improved morphology with larger grain size. This additive also modulates carrier recombination and extraction dynamics at the buried interface via formation of a bottom-up 2D/3D heterojunction. This strategy enabled high-quality MA-free perovskite films with improved crystallization, fewer trap states, and enhanced interfacial hole extraction and transport, which stabilized this important interface and minimized nonradiative recombination losses. The resultant inverted device delivered a PCE of 25.12% (certified 24.6%), with an increase of Jsc from 25.36 to 26.22 mA cm^–2^.

On the other hand, FF is strongly affected by the series resistance (*R*_s_) and shunt resistance (*R*_sh_), as well as charge carrier recombination.^129,135^ In general, *R*_s_ is determined by the resistance of the device materials and contact resistance, whereas *R*_sh_ arises from the leakage current at the pinholes and shunting pathways. To obtain a higher FF, a lower *R*_s_ and a larger *R*_sh_ are needed.^129,136^ Defect passivation using additives, such as polymers, Lewis acid/base agents and organic molecules, helps to obtain smooth perovskite film with less pin-holes and defect trapping states, leads to an increase of FF.^137^ For instance, Cao et al.^138^ reported an effective and universal strategy via a star-shaped polymer (PPP) passivation to increase FF for different perovskites such as MAPbI_3_, MA_0.5_FA_0.5_PbI_3_ and triple-cation perovskite. A high FF of 0.862 has been achieved for inverted triple-cation PSCs with a PCE of 22.1%. The enhancement of FF is attributed to the fact that the PPP molecule acts as a 3D skeleton template to control crystallization and to passivate defects at GBs and the surface, as well as to reduce nonradiative recombination, charge-transport losses, and ion migration.

Over the past decade, the efficiency of PSCs has increased monotonically, improving from 3.8 ^9^ to 26.08%.^139^ The record *J*_SC_ (26.7 mA cm^2^)^140^ and FF (86%)^141^ of PSCs are approaching their theoretical limit. The *V*_oc_ loss (or *V*_oc_ deficit) is defined as (E_g_/q) - *V*_oc_, where *E*_g_ is the optical bandgap of the perovskite material, and *q* is the elementary charge. Much effort has been undertaken to reduce *V*_oc_ deficit by controlling the perovskite crystallization, defect passivation, interface engineering and formation of graded junctions.^142^ However, the *V*_oc_ deficit of the best performing PSCs is at ∼0.34–0.41 V, which is still above the losses based on the Shockley–Queisser (SQ) limit (∼0.27 V). In addition, for wide bandgap perovskite materials with >2 eV, the *V*_oc_ loss is still ∼0.5 V and larger.^143^ The *V*_oc_ of a solar cell is directly related to the quasi-Fermi level splitting and is determined by the difference between the quasi-Fermi levels for electrons and holes. The quasi-Fermi level splitting is controlled by the charge density. Any defect-related nonradiative recombination can reduce the steady-state charge density, resulting in a decrease of *V*_oc_. The relationship between the PLQY and the achievable *V*_oc_ in perovskites at 1-sun can be expressed as follows (eq 9).^144^

9 $$V_{\mathrm{oc}}=V_{\mathrm{oc,rad}}+\frac{k_{B}T}{q}\ln\left(\mathrm{PLQY}\right)$$

where the *V*_oc_ is the expected open-circuit voltage, *V*_oc,rad_ represents the radiative (ideal) limit of the open-circuit voltage, *k*_B_ is the Boltzmann constant, and *q* comprises the elementary charge.

In general, the PLQY is affected by the quality of perovskite film and the energy level of the interfacial charge-extraction/transport layers.^145^ Therefore, one of the major strategies for improving *V*_oc_ is to lower the charge recombination rate and increase the carrier lifetime as well as optimize the energy level alignment between the halide perovskite and the charge transport layer. Grain-boundary and surface passivation using small-molecule, Lewis acid/base agents or polymeric passivators effectively reduces deep trap densities and extends carrier lifetimes.^135^ Beyond chemical passivation, 2D/3D perovskite heterojunction offers an additional pathway by combining defect passivation with interfacial energetic modulation, thereby reducing interfacial recombination and promoting efficient charge transport.^133^

### Classification of Passivating Agents in Perovskite Solar Cells

As discussed above, the PCEs and stability of PSCs are often affected by factors such as surface defects, grain boundaries, pinhole formation, and moisture sensitivity. Surface passivation by ligand engineering has emerged as a powerful strategy for improving the efficiency and stability of PSCs. Ligands can be classified from small organic and inorganic molecules to organic–inorganic hybrid salts and random polymers. Such ligands help to improve the stability and performance of PSCs by passivating halogen and A-site cation defects, mitigating pinholes, and enabling active carrier extraction and transport. Commonly used ligands include fullerenes (C_60_ and C_61_) and their derivatives, alkyl and aryl amines and ammonium salts, thiols, alcohols, carboxylic acids, esters, carbon quantum dots, graphitic carbon nitride, carbon nanotubes, organic dyes, and certain metal oxides and salts. Incorporating these ligands onto halide perovskites in PSCs helps achieve excellent stability and PCE, including high values of the open-circuit voltage, short-circuit current, and fill factor. In the following, we discuss different kinds of common passivating ligands and passivation mechanisms, the improvement in PCEs and the stability of PSCs by their incorporation.

#### Fullerene-Based Ligands

Buckminsterfullerene (fullerene) and its derivatives have been utilized not only as excellent electron acceptors for interfacial electron transfer but also as passivators in PSCs. Its inherent icosahedral and π-conjugated structure makes it highly electron-deficient, which makes fullerene an excellent multielectron acceptor with a low reduction potential, resulting in neutralizing trap states. They accept excess electrons from defect states and form delocalized charge-transfer complexes and suppresses nonradiative recombination. A large number of C_60_ derivatives with various functional groups (e.g., −COOH, −NH_2_, −OH, −SH) have played a significant role in the development of high-efficiency PSCs, as summarized in Table 1.^125,146–159^ These derivatives differ in their modes of interfacial electronic coupling, defect passivation, pinhole reduction, and efficiency rollout, determined by their functional groups, amine, ammonium ion, carboxylic acid, cyano, ester, thiol, and alcohol. These functional groups can fill either halide vacancies by coordination with B-site cations or compensate for A-site cation vacancies. Additionally, functional groups, including carboxylic acids,^146,151,157^ esters,^146,147,154–157^ amines,^150,152,153^ alcohol,^157^ and cyanine,^149^ help bind efficiently to the electron transport materials such as TiO_2_. Hence, it has been widely used to alter the alignment at the electron transport layer (ETL)/perovskite junction.

**Table 1:** List of Fullerene Ligands in PSCs and the Corresponding PCE and FF Values

perovskite	ligand	PCE (%)	FF (%)	ref
FA_0.85_MA_0.15_-Pb(I_*x*_ Br_1–*x*_)_3_	[6,6]-phenyl-C_61_-butyric acid methyl ester (PCBM) and [6,6]-phenyl-C_61_-butyric acid (PCBA), SnO_2_	14.7–18.8	64–76	^146^
FA_*x*_MA_1–*x*_-Pb(I_*y*_Br_1–*y*_)_3_	[6,6]-phenyl-C_61_-butyric acid-*N*,*N*-dimethyl-3-(2-thienyl)propanam ester (PCBB-S-N) containing a sulfur atom and C_60_	18.61–21.08	72.25–79.09	^147^
MAPbI_3_	fullerene electrolyte (PCBB-3N-3I)	17.7–21.1	81.36	^148^
MAPbI_3_	triblock fullerene derivative (PCBB-2CN-2C8)	12.58–20.7	72.2–79.1	^149^
FA_*x*_MA_(1–*x*)_-Pb(I_1–*y*_Br_*y*_)_3_	[6,6]-phenyl-C_61_-butyric acid methyl ester (PCBM)	18.47–23.13	74.92–81	^150^
MAPbI_3_	amino-functionalized fullerene derivative (C_60_-BPAM)	19.36–20.21	78.34–78.53	^151^
FAPbI_3_	C_60_-porphyrin conjugate	22.93	81.67	^152^
(Cs_0.17_FA_0.83_)-Pb(I_0.8_Br_0.2_)_3_	bis-fulleropyrrolidium iodide (bFPI)	21.11	81.3	^153^
FA_0.85_MA_0.15_-Pb(I_*x*_ Br_1–*x*_)_3_	fullerene self-assembled monolayer (C_60_-SAM), benzoic acid	15.7–17.3	75	^155^
FA_0.85_MA_0.15_-Pb(I_*x*_Br_1–*x*_)_3_	PCBM and its polymer	15.7, 10.4	71.4, 48.7	^156^
FAMASnI_3_	PCBM, NiO, CuI	21.51 (MASnI_3_) to 24.27 (FAMASnI_3_)	80.08–84.30	^157^
FA_0.85_MA_0.15_-Pb(I_*x*_Br_1–*x*_)_3_	fullerene bis(phenethyl alcohol) malonate (FMPE-I), fullerene bis(ethylenediamine) malonamide (FEDA-I), and fullerene bis(propanediamine) malonamide (FPDA-I), phenyl-C_61_-butyric acid methyl ester (PCBM)	14.96–17.63	75.23–78.43	^158^
FA_0.95_Cs_0.05_PbI_3_	PCBM, tetramethylthiuram disulfide (TMDS)	22.9–26.10 (certified 25.39)	83.7	^125^
Cs_0.04_MA_0.14_FA_0.82_-Pb(I_0.987_Br_0.013_)_3_	4-(1′,5′-dihydro-1′-methyl-2′*H*-[5,6]fullereno-C_60_-Ih-[1,9-*c*]pyrrol-2′-yl)-phenylmethanaminium chloride (CPMAC)	25.5–26.1	84.9–85.5	^159^

A few studies have found that Fullerene and its derivatives improve the film uniformity and grain boundary quality of perovskites during crystallization. Fullerene derivatives applied to PSCs include [6,6]-phenyl-C_61_-butyric acid methyl ester (PCBM), [6,6]-phenyl-C_61_-butyric acid (PCBA),^146^ [6,6]-phenyl-C_61_-butyric acid-*N*,*N*-dimethyl-3-(2-thienyl)­propanam ester (PCBB-S-N),^147^ an iodide quaternized fullerene derivative terminated with tris­(dimethylamine) (PCBB-3N-3I),^148^ [6,6]-phenyl-C_61_-butyric acid-dioctyl-3,3′-(5-hydroxy-1,3-phenylene)-bis­(2-cyanoacrylate) ester (PCBB-2CN-2C8),^149^ phenyl-C_61_-butyric acid methyl ester (PCBM),^150^ functional fullerene with a porphyrin ring and three pentafluorophenyl groups (FPD),^152^ pyrrolidine-phenyl-functionalized fullerene derivative (C_60_-BPAM) and bis-fulleropyrrolidium iodide (bFPI).^153^ The PSCs passivated with these fullerene ligands, along with corresponding improvements in PCE and FF, are summarized in Table 1. For example, Abrusci et al. introduced a self-assembled monolayer (SAM) of fullerene as the interfacial transport layer between TiO_2_ and CH_3_NH_3_PbI_3–*x*_Cl_*x*_ perovskite layer.^154^ They found that the fullerene SAM forms a selective bridge that extracts electrons from the perovskite while blocking direct electron transfer to TiO_2_, suppressing recombination, which leads to an increase in PCE from 3.8% (only TiO_2_) to 11.7% and FF from 65% to 72%.

Subsequently, numerous C_60_ derivatives have been introduced for interface engineering to enhance the passivation and long-term stability. In this regard, Sanith and co-workers^154^ functionalized C_60_ with benzoic acid and used it as a rigid spacer for electronic coupling. It strongly binds to TiO_2_, offering stronger electronic coupling and allowing ultrafast electron transfer from the perovskite to TiO_2_, leading to an increase in PCE of 17.3% with an FF of 75%. Similarly, Zhang et al. designed a Lewis acid functionalized fullerene derivative terminated with tris­(dimethylamine) (PCBB-3N-3I).^148^ The iodide site in the ligand binds to the surface defects of the perovskite, thus, reducing the charge carrier recombination at the interface. These ionic anchoring combined with steric modulation ensures that the ligand forms a stable SAM with its dipole pointing from the perovskite to the ETL, resulting in improved performance and stability with a 21.1% PCE, 81.36% FF, 1.105 V *V*_oc_, and 23.46 mA cm^–2^ *J*_sc_.

Later, Li et al. developed a p-i-n (inverted) PSC in which the perovskite layer is capped with PCBM,^150^ resulting in halide ion diffusion from the perovskite layer to the PCBM layer, thereby improving electrical conductivity. However, this could deplete halide ions on the perovskite surface, which was prevented by predoping the transport layer with halides, thereby stabilizing and preventing degradation. The fabricated device yielded a high PCE, increasing from 18.47% (pristine) to 23.1%, and an improved FF, rising from 74.9% to 81%. Recently, Gong et al. obtained a high PCE of 26.1% (certified 25.39%) and an FF of 84.7% using a fullerene derivative.^125^ The commonly used PCBM suffers from several issues, including poor intrinsic conductivity, a tendency to form aggregates, and inadequate defect passivation. To overcome these issues, they conducted a phototriggered molecular doping strategy using tetramethylthiuram disulfide (TMDS). Figure 3 shows the scheme demonstrating the device fabrication, passivation mechanism, efficiency, and stability of the device fabricated with TDMS, compared to those of a PCBM-only device. Under UV light, the TMDS cleaves and liberates sulfur radicals, which are then donated to the PCBM, leading to an increase in carrier concentration and mobility by 20 times. Simultaneously, TMDS passivates the perovskite surface defects by coordinating with Pb^2+^ ions at the interface. Moreover, under UV light, TMDS readily forms radicals, which facilitates n doping of the electron acceptor PCBM. As a result, it significantly suppresses charge recombination and homogeneous electron transport. The device retains 95% of its initial stability after 1090 h under 85 °C. Overall, incorporating TMDS with PCBM enhances the built-in electric field, high efficiency, and commercial-grade device stability of PSCs.

**Figure 3:**
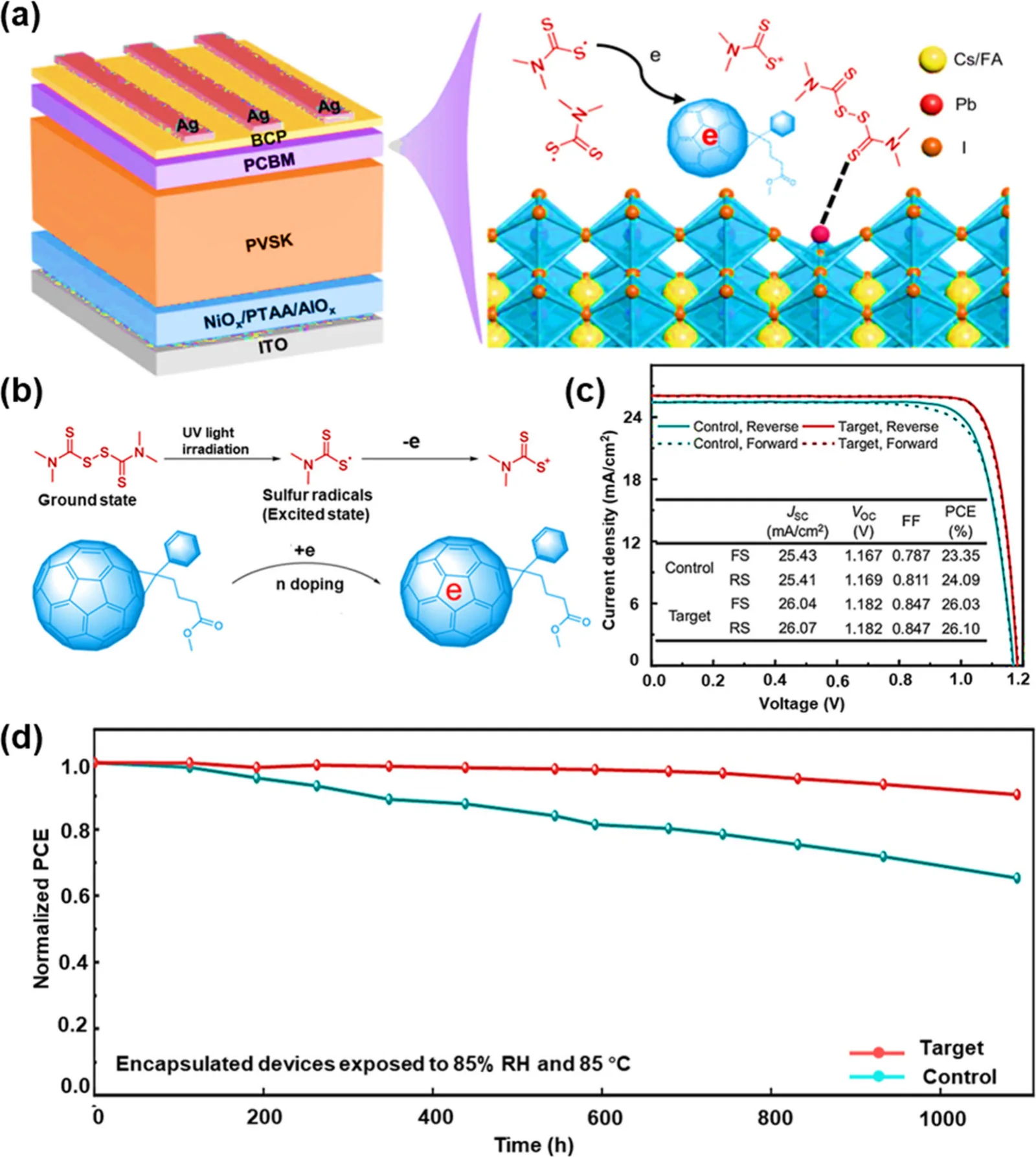
(a) A scheme of a PSC fabrication using PCBM and TDMS and the chemical interaction between ligands and the perovskite structure, (b) the mechanism of sulfur radical formation from TDMS and doping with PCBM, (c) I–V characteristics of the PSC under forward and reverse scans with the PCBM as the control cell, and (d) the stability of the TDMS incorporated device at 85 °C and 85% humidity for 1090 h. Panels (a–d) reproduced from ref 125. Available under CC-BY 4.0. Copyright 2024 The Author(s). Published by Springer Nature.

In another study, by replacing C_60_ with an ionic salt, 4-(1′,5′- dihydro-1′-methyl-2′H-[5,6] fullereno-C_60_-Ih-[1,9-*c*]­pyrrol-2′-yl) phenylmethanaminium chloride (CPMAC) in inverted (p-i-n) PSCs, You et al. obtained a certified champion PCE of 26.1%, FF of 85.5% *J*_sc_ of 26.0 mA cm^–2^, and *V*_oc_ of 1.18 V.^159^ They introduced the CPMAC into the interface of the perovskite and Ag-SnO_*x*_. electrode. It was proposed that the CPMAC enhances the interface binding through its CH_2_–NH_3_^+^ cation, forming a stronger chemical and ionic interaction with the perovskite surface sites, resulting in a significant reduction of the ETL/perovskite interface roughness compared to C_60_, thereby enhancing electronic coupling and electron mobility and effectively facilitating efficient and stable charge transfer across the interface. The fabricated device showed higher stability and maintained 91.5% of its initial PCE after 2200 h of continuous light illumination at 55 °C.

#### Alkyl and Aryl Amines and Their Corresponding Ammonium Salts

The Lewis base (molecules or ions that have lone-pair electrons) defect passivation properties, coordination with the B-site, compensation for surface A-site cations, and halide vacancy filling by amines or ammonium ions are vital for improving the efficiency and stability of PSCs. Lewis bases, such as thiocyanate SCN^–^, pyridine, amines, thiols, and phosphines, donate lone-pair electrons and coordinate to the metal defect sites. Therefore, amines and ammonium salts have been extensively used in PSCs for defect passivation, pinhole suppression, and efficiency improvement.^42,160–191^ Although it is difficult to classify amines and ammonium salts based on their impact on PCE or FF improvement, alkyl phenyl amines and ammonium salts appear to be the most promising ligands.^160–164^ These molecules are used alone or in combination with other ligands, such as polyamines,^162–172^ thiocyanates,^173^ organic/inorganic salts,^42,174–183^ thiophenes,^184^ thioles,^185^ C_60_ and its derivatives,^187–189^ and urea.^190^ Several studies have also demonstrated the advantages of bifunctional amine or ammonium-based ligands with their second as thiols, carboxylic acids, and organic dyes and C_60_ derivatives, which enables simultaneous passivation of both the top and bottom surfaces and grain boundaries of the perovskite layer. Some of these ligands, their corresponding perovskites, and the resulting PCE and FF values of PSCs reported in the literature are given in Table 2. Interestingly, the use of phenylethylammonium chloride/bromide/iodide capping on the perovskite layer resulted in an increase of PCE and FF from 8.68% to 29.8% and from 60% to 84%, respectively. Besides, a few early studies have shown that spin coating phenylethylammonium iodide (PEAI) on perovskite layer produces a smooth surface layer by its coordination with the Pb^2+^ ions at the grain boundaries and subsequent passivation of surface defects.^191^ For instance, Xu et al. demonstrated that PEAI accumulates at the grain boundaries and surfaces by forming a uniform film with fewer pinholes on the MAPbI_3_/hole transport layer (HTL) surface.^191^ The PEAI significantly passivates defects by chemically binding to uncoordinated lead ions, thereby reducing defect-assisted and nonradiative recombination and facilitating efficient hole extraction and transport through the perovskite. However, the fabricated device achieved a lower PCE of 8.68% with a FF of 60% only.

**Table 2:** List of Alkyl Amines, Aryl Amines, and Ammonium Salts in PSCs and the Corresponding PCE and FF Values

perovskite	ligand	PCE (%)	FF (%)	ref
FA_0.8_Cs_0.2_-Pb(I_*x*_Br_1–*x*_)_3_	phenethylammonium iodide (PEAI)	18.64–24.1	NA	^161^
CsPbI_3_	benzoyl hydrazine (BH)	20.47	81.23	^162^
Cs_0.05_(FA_0.95_MA_0.05_)_0.95_-Pb(I_0.95_Br_0.05_)_3_	piperazinium iodide (PI)	23.37 (22.26 ± 0.49)	80.17 (79.62 ± 0.94)	^163^
MAPbI_3_	phenylhydrazinium iodide (PHAI)	18.18	76.00	^164^
FA_0.83_Cs_0.17_-Pb(I_0.75_Br_0.25_)_3_	ethylenediamine (EDA)	19.07	82	^165^
FA_0.85_MA_0.15_-Pb(I_*x*_Br_1–*x*_)_3_	PEAI on HC(NH_2_)_2_–CH_3_NH_3_	20–23.32	79–81.4	^166^
FA_0.85_MA_0.15_-Pb(I_*x*_Br_1–*x*_)_3_	PEAL and ethylenediamine diiodide	17.07–22.51	71.68–81.17	^167^
CsFAMA mixed perovskite	3-(1-pyridinio)-1-propanesulfonate (PPS)	21.7	81.2	^168^
CsPbI_3–*x*_Br_*x*_	tryptamine (TA)	21.8	83.9	^169^
FAPbI_3_	4-dimethylaminopyridine (DMAP)	22.41	78.08	^170^
CsPbI_3–*x*_Br_*x*_	histamine (HA), 2-(1*H*-imidazol-4-yl)ethanamine	20.8	81.9	^171^
FA_0.85_Cs_0.15_-Pb(I_*x*_Br_1–*x*_)_3_	methylammonium thiocyanate (MASCN) and PEAI	20.2–29.8	80.6–83	^173^
FA_0.85_MA_0.15_-Pb(I_*x*_Br_1–*x*_)_3_	4-MeO-PEACl	18.39–22.63	74.05–82.31	^174^
FA_0.8_Cs_0.2_-Pb(I_*x*_Br_1–*x*_)_3_	PEAI and PI	16.26–26.36	72.51–73.62	^175^
FA_0.65_MA_0.20_Cs_0.15_-Pb(I_0.80_Br_0.20_)_3_	phenylammonium bromide (PhABr), phenmethylammonium bromide (PMABr) and phenethylammonium bromide (PEABr)	15.72 to PMABr (17.32)	76.56–78.21	^176^
		PEABr (16.83)		
		PhABr (15.98)		
Cs_0.05_(MA_0.15_FA_0.85_)_0.95_-Pb(I_0.85_Br_0.15_)_3_	MAI, PEA_2_PbI_4_	20.80	73	^177^
Cs_0.15_FA_0.85_-Pb(I_0.95_Br_0.05_)_3_	1,4-butanediamine dihydriodide (BDAI_2_)	23.1	82.1	^178^
Cs_0.05_MA_0.1_FA_0.85_-PbI_2.9_Br_0.1–0.05_PbI_2_	cyclohexylethyl-ammonium iodide (CEAI)	23.57	82.6	^42^
(FAPbI_3_)_1–*x*_-(MAPbBr_3_)*x*	4-fluorophenethyl-ammonium iodide (F-PEAI)	21.0	77.5	^179^
(FA_0.85_MA_0.1_Cs_0.05_-Pb(I_0.97_Br_0.03_)_3_)	fluoroethylamine (FC_2_H_4_NH_3_, FEA)	23.40	81.27	^180^
CsPbI_3_	4-amino-2,3,5,6-tetrafluorobenzoate cesium (ATFC)	21.11	82.82	^182^
((FAPbI_3_)_1–*x*_-(MAPbBr_3–*y*_Cl_*y*_)_*x*_)	PEAI	22.98	77.79	^183^
CsFAMA mixed perovskite	poly(bithiophene imide) (PBTI)	20.67 (19.66 ± 0.43)	79.8 (77.7 ± 1.3)	^184^
CsFAMA perovskite	cysteamine hydrochloride (CSA-Cl)	19.12 ± 0.90	77.35 ± 2.68 (80.22)	^185^
		30 (20.98)		
MAPbI_3_	2-aminoterephthalic acid	21.09	78.62	^186^
MAPbI_3_	Lewis base, TBA-Azo	18.3–19.6	73.8–76.0	^187^
	PCBM			
CH_3_NH_3_PbI_3_	C_60_NH_2_	15.92–18.34	68–76	^188^
FAPbI_3_	Lewis base urea additive	16.80–18.25	72–75	^190^

In another study, Wei et al. reported that spin-coating a PEAI layer onto Sn–Pb perovskite layer produces an ultrathin 2D capping layer,^160^ which passivates the traps with bulky PEA^+^ cations, leading to the stability enhancement by suppressing the oxidation of Sn^2+^ to Sn^4+^, and also acts as a good charge transport layer, resulting in a much higher PCE of 23.7% with an FF of 80.4%. Similarly, PEAI on a wide-bandgap perovskite [FA_0.85_Cs_0.15_Pb­(I_0.6_Br_0.4_)_3_]-HTL layer,^161^ resulted in an increase of the activation energy for ion migration, thereby suppressing halide ion migration and increasing the open circuit voltage (1.25 V). This led to an appreciable increase in PCE from 18.64% (without PEAI) to 24.1%.

In addition, PEAI with other ligands has also been used in combination to give bifunctional advantages. For example, when piperazinium iodide was used along with PEAI on perovskite layer,^175^ their bifunctional ammonium groups modify the dipole moments, resulting in a lower work function at the ETL/perovskite interface. This results in suppressing the deep-level traps, enhancing the built-in electric field at the ETL interface and improving energy-level alignment. The device exhibited an increase of PCE and FF from 16.26% (only PEAI) to 26.36% and from 72.75% to 73.62%, respectively. Recently, Liu et al. obtained a champion PCE of 29.8% (4-T tandem) with an FF of 83% by incorporating methylammonium thiocyanate (MASCN) with PEAI.^173^ This dual-ligand 2D perovskite capping strategy effectively passivates defects at both the perovskite grain boundaries and the HTL interface, suppressing interface degradation and phase instability. Consequently, the device enhanced stability and maintained nearly 100% of its initial PCE after 500 h of continuous illumination. Hence, bifunctional passivation enables enhanced thermal, photo, and ambient stability.

Generally, alkyl amines act as Lewis bases and coordinate to Pb^2+^/Sn^2+^ ions on perovskite surfaces, thereby passivating defects, reducing nonradiative recombination, enhancing film quality, and improving long-term stability. For example, alkyldiammonium iodides such as 1,4-butanediammonium iodide (BDAI_2_)^178^ contain two NH_3_^+^ groups, which passivate both Pb^2+^ and halide vacancies at the perovskite/ETL interface by forming a dense and uniform interfacial layer that promotes efficient carrier extraction. Moreover, the bidentate ammonium groups of BDAI_2_ match well to synergistically passivate two defect sites on the surface of the perovskite unit cell. The corresponding PSCs demonstrated a high PCE of 23.1% with an FF of 82.1%, and retains 92% of its initial PCE after 1000 h of continuous illumination. Likewise, cyclohexylethylammonium iodide (CEAI),^42^ introduced at the perovskite/HTL interface, not only blocks trap-assisted carrier recombination at the interface but also protects the PSC from moisture, enhancing cell stability with its hydrophobic cyclohexyl group (Figure 4). The CEAI on the perovskite layer produces a 2D layer with a CEA_2_PbI_4_ quasiperovskite phase, which modifies the energy-level alignment and facilitates efficient hole extraction to the HTL layer. The primary amine group in the CEAI binds with Pb^2+^, effectively passivating deep traps, suppressing halide ion migration, and reducing nonradiative recombination. Simultaneously, the cyclohexyl ring imparts a hydrophobic environment, thereby improving moisture resistance and long-term operational stability. The corresponding PSC exhibited a PCE of 23.57% with a FF of 82.1%, and the cell retained 96% of its initial PCE after 1500 h under continuous sunlight. Figure 4 shows the corresponding device and its passivated surface structure, stability, *I*–*V* characteristics, and PCE.

**Figure 4:**
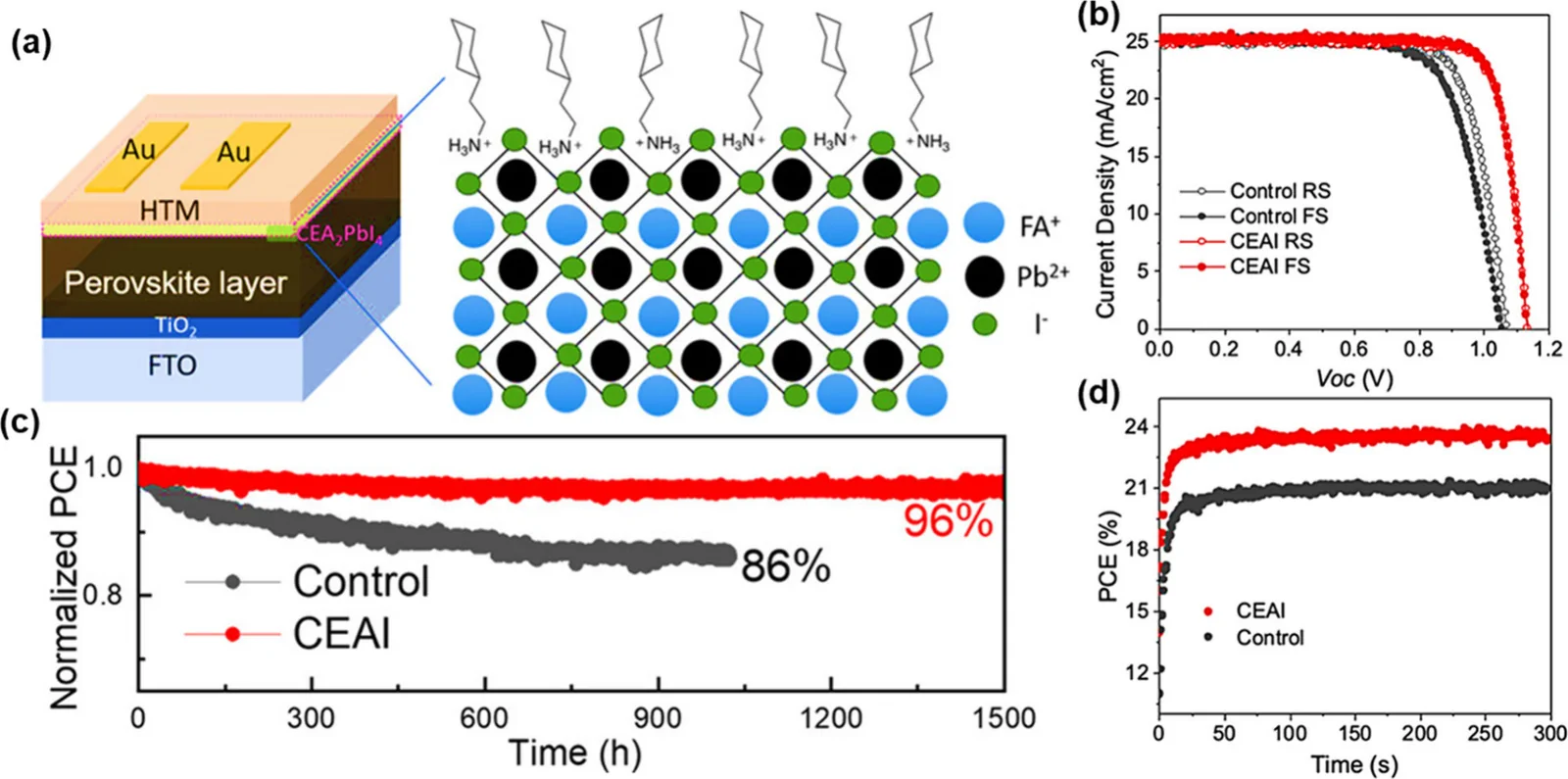
(a) Scheme of a FAPbI_3_ PSC with CEAI and the chemical interaction between the ligands and the perovskite structure, (b) comparison of *I*–*V* characteristics under forward and reverse scans with and without CEAI, (c) stability of the CEAI-treated device under sunlight for 1500 h, and (d) PCE with time. Panels (a)–(d) reproduced from ref 42. Available under a CC-BY 4.0. Copyright 2021 The Author(s). Published by the American Chemical Society.

Additionally, incorporating heteroatoms, such as halogens, into amines enhances their interaction with the perovskite surface. For example, fluoroethylamine (FEA)^180^ acts as a multifunctional surface passivator, where the amine group coordinates with the Pb^2+^ cation for surface passivation, and the fluorine atom forms hydrogen bonds and electrostatic interactions, inducing a strong dipole moment that aligns the energy level between perovskite and ETL. Moreover, this dipole enhances the built-in electric field, suppressing ion migration, and stabilizes surface and bulk defects, resulting in a higher PCE of 23.4%, an FF of 81.27%, and long-term device stability. Amines with other functional moieties such as thiols (cysteamine hydrochloride),^185^ carboxylic acids (2-aminoterephthalic acid),^186^ pyridine (4-dimethylaminopyridine),^170^ and imides (poly bithiophene imide)^184^ also exhibit higher PCE (>20%), enabled by the ability to coordinate via multiple binding sites. Therefore, such bifunctional molecules help to reduce trap densities, facilitating efficient interfacial charge transfer and higher PCE and operational stability.

Beyond the effects of surface termination and processing conditions, the contact layer with which the perovskite is paired can also introduce defects at the interface. For example, Boyd et al. found that Ni^3+^ sites in solution-processed nickel oxide (NiO_*x*_) acted as a Brønsted base (proton acceptor) and Lewis acid (electron acceptor), triggering the deprotonation of the perovskite and oxidation of iodide species. This gives rise to the formation of a PbI_2–*x*_Br_*x*_-rich layer, which increases nonradiative recombination and acts as a hole extraction barrier. The authors found that that these effects could be mitigated by adding excess alkylammonium iodide, enabling 19.7%-efficient perovskite solar cells to be achieved, with the open-circuit voltage improving from 0.90 to 1.15 V.^192^

#### Carboxylic Acids and Esters

Multidentate carboxylic acids and esters serve as effective chelating ligands that can passivate defects in perovskite films. They can readily donate lone pairs of electrons from the oxygen in the carboxylate or hydroxyl group to coordinate with Pb^2+^ ions on the perovskite surface, leading to a thermodynamically stable surface at the interface. Table 3 summarizes some of the PSCs passivated with these ligands, with or without esters, carbon quantum dots, graphitic carbon nitride, or carbon nanotubes, and their corresponding PCE and FF.^193–199^ For instance, the chelation effect of various carboxylic acids, such as citric acid, tartaric acid, malic acid, and adipic acid, has been systematically studied by depositing them at the perovskite/HTL interface.^193^ It was found that carboxylic acids passivate the perovskite grain boundaries by forming strong multiple Pb–O chelation complexes, thereby reducing the number of traps, enhancing ion migration, improving surface roughness, and increasing the PL intensity. Among these acids, citric acid was proved to be the most efficient, increasing the PCE from 20.7% to 22.5%, enabled by the three carboxylic acid groups, which facilitate efficient coordination. Moreover, the corresponding PSC with a citric acid passivator exhibited a PCE exceeding 90% of its initial value under continuous illumination in ambient air.

**Table 3:** List of Carboxylic Acids, Esters, Carbon Quantum Dots, Graphitic Carbon Nitride, and Carbon Nanotubes in PSCs and Their Corresponding PCE and FF Values

perovskite	ligand	PCE (%)	FF (%)	ref
MA/FAPbBr_3_	adipic acid, malic acid, tartaric acid, and citric acid	20.7–22.05 (CA)	NA	^193^
Rb_0.02_(FA_0.95_Cs_0.05_)_0.98_-PbI_2.91_Br_0.03_Cl_0.06_	1-adamantaneacetic acid (ADAA)	22.25 ± 0.239	82.7 ± 0.5	^194^
FAPbI_3_	γ-valerolactone (GVL) and *n*-butyl acetate	∼18 (DMF/DMSO) to 25.09	70–82	^195^
MAPbI_3_	graphitic carbon nitride (*g*-C_3_N_4_)	16.535–19.49	65–74	^196^
FA_1–*x*_MA_*x*_PbI_3_	natriumion-functionalized carbon nanodots	20.42	NA	^197^
NH_3_CH_3_PbI_3_-based perovskite	4,6-di(anthracen-9-yl)-1,3-phenylene bis(dimethylcarbamate), carbon nanotube-added NH_3_CH_3_PbI_3_-based perovskite solar cells	18.4–20.7	79	^198^
(FAPbI_3_)_0.95_-(MAPbBr_3_)_0.05_	multifunctional carbon quantum dots	22.31–24.48	80.84–84.54	^199^
Cs_0.05_(FA_0.83_MA_0.17_)_0.95_-Pb(I_0.83_Br_0.17_)_3_	pentafluorophenyl acrylate (PFPA)	22.42	79.37	^199^
(FAPbI_3_)_0.875_-(MAPbBr_3_)_0.075_-(CsPbI_3_)_0.05_-(PbI_2_)_0.03_	merocyanine	22.04 (21.15 ± 0.51)	81.00 (79.00 ± 1)	^199^
(FAPbI_3_)_0.85_-(MAPbBr_3_)_0.15_	organic dye (AQ310)	19.19	76.8	^199^

In another study, a series of adamantane carboxylic acids, such as 2-adamantanone (AD), 1-adamantane carboxylic acid (ADCA), and 1-adamantaneacetic acid (ADAA) passivators, were introduced on the SnO_2_/perovskite interface.^194^ Depending on the distance between the carbonyl group and the SnO_2_/perovskite interface, steric hindrance governs the extent to which each molecule can interact with the interface. It was found that ADAA is the most sterically hindered modifier, being too bulky to penetrate deeply into the interface, resulting in a more uniform capping layer with fewer defects. Also, the carbonyl group of ADAA helps its chelation with metal ions (Pb^2+^/Sn^4+^), and they effectively block the diffusion of moisture and oxygen. The corresponding PSC demonstrated a higher PCE of 23.18% and FF of 84.1%. Moreover, ADAA suppresses mechanical degradation and improves the long-term thermal stability of PSCs. Recently, the use of greener solvents, such as γ-valerolactone (GVL), has become attractive owing to the exceptional stability they provide to perovskite precursor inks.^195^ GVL promotes a stronger interaction between FA^+^ and ([PbI_*x*_]^2–*x*^) species, thereby preventing chemical decomposition of FA^+^. It also facilitates controlled and uniform film formation with larger grains. The corresponding PSC fabricated using GVL exhibited an excellent increase in PCE, from 18% (DMF/DMSO) to 25.09% (GVL), and FF, from 70% to 82%. Additionally, it exhibited stability against heat and moisture, maintaining over 95% of its initial PCE after 1000 h of continuous illumination.

#### Carbon Quantum Dots, Graphitic Carbon Nitride, and Carbon Nanotubes

Carbon nanomaterial additives, including carbon quantum dots, graphitic carbon nitride, and carbon nanotubes, offer significant advantages for PSCs, such as defect passivation, efficient charge transport, and enhanced stability. Some of these ligand additives, the corresponding PSCs, along with their PCEs and FFs, are listed in Table 3. A few early studies have shown that the use of graphitic carbon nitride (g-C_3_N_4_) additive at the perovskite/ETL layer slows the crystallization rate of MAPbBr_3_, thereby forming a uniform layer with larger grains.^196^ The nitrogen atoms of g-C_3_N_4_ coordinate with Pb^2+^ cations and passivate defects in the grain boundaries, forming a conductive network that reduces the grain boundaries and enables better charge extraction. This results in an increase in PCE from 16.53% to 19.49% with an FF from 65% to 74%.

Another study demonstrated the use of cation-functionalized carbon nanodot additives in PSCs, resulting in a higher PCE of 20.42%.^197^ It was found that the doping of carbon nano dots with sodium ions leads to a reduction of trap-assisted recombination by passivation of the grain boundaries and halide vacancies at the perovskite surface. Na^+^-doping enables favorable energy level alignment, which helps the efficient extraction of electrons from the perovskite to the ETL. Moreover, the device exhibited long-term stability, retaining approximately 90% of its initial PCE after 1000 h of continuous illumination under ambient air conditions. Likewise, the use of bifunctional ligands prepared by functionalizing polyaromatic nanotweezers, such as 4,6-di­(anthracene-9-yl)-1,3-phenylene bis­(dimethylcarbamate), with functionalized single-walled carbon nanotube (s-SWCNTs) in PSCs with over 20% PCE.^198^ In this case, the polyaromatic anthracene group forms π–π stacking with CNT and the carbamate group, which then interacts with the uncoordinated lead cations, facilitating perovskite grain growth and defect passivation. SWCNTs in contact with the perovskite serve as efficient one-dimensional conductive pathways to the ETL, facilitating efficient charge extraction and transport. The corresponding PSC exhibited a higher PCE of 20.7%; however, ∼10% of its initial PCE drops after 300 h of illumination in ambient. Recently, a champion efficiency of 24.48% has been obtained using carbon quantum dots functionalized with carbonyl and amino groups as additives.^199^ The carbonyl group coordinates with the Pb^2+^ cations on the perovskite surface, whereas the amine group interacts with organic cations and halides. This dual functionality of carbon quantum dots chemically passivates the surface defects, optimizes the energy-level alignment, and shields the perovskite from moisture.

#### Organic Dyes

Organic dyes or molecules with an extended π-conjugated system that efficiently absorb visible light have been found to improve the PCE and FF of PSCs.^200–202^ Various dyes have been incorporated within the perovskite layer primarily to enhance light absorption, improve charge transfer, and facilitate defect passivation. Table 3 summarizes some of these dye-incorporated PSCs, along with their corresponding PCEs and FFs. For instance, the incorporation of organic dye, pentafluorophenyl acrylate (PFPA) with multifunctional groups, at the perovskite/HTL layer leads to an improvement of PCE of PSCs.^200^ PFPA not only passivates surface defects and suppresses nonradiative recombination pathways but also modifies the energy level alignment at the interface between the Cs_0.05_(FA_0.83_MA_0.17_)_0.95_Pb­(I_0.83_Br_0.17_)_3_/HTL layer, resulting in more efficient hole extraction from the perovskite layer and transfer to the HTL. This synergistic effect enhances the photovoltaic performance, resulting in a higher PCE of 22.42% with an FF of 79.39%. Similarly, bifunctional organic dye with conjugated and zwitterionic structure, merocyanine, acts as an efficient hole extractor from the (FAPbI_3_)_0.875_(MAPbBr_3_)_0.075_(CsPbI_3_)_0.05_(PbI_2_)_0.03_ perovskite and transports the holes to the electrodes.^201^ Additionally, its zwitterionic structure and Lewis base character enable passivation of surface defects, excellent charge transport, and energy level modulation, resulting in a PCE of 22.4% with an FF of 81%. Another example is AQ310, an organic dye with a carboxylic acid functional group that also serves as an efficient passivation layer at the HTL/perovskite interface.^202^

#### Inorganic Oxides and Salts

Inorganic compounds such as metal oxides represent another class of ligands and interface additive materials used in PSCs.^203–215^ They serve as excellent charge-transport layers and interfacial modifiers. Some examples include zinc oxide (ZnO), tin oxide (SnO_2_),^203,204^ aluminum-doped zinc oxide (AZO),^205,206^ and vanadium oxide (V_2_O_5_).^207^ Table 4 summarizes PSCs made of some a of these ligands, along with their corresponding PCEs, and FFs. For example, a few earlier studies have revealed that the incorporation of ZnO into MA-free PSCs results in excellent chemical stability with PCE of 20.3%.^204^ Similarly, the ZnO–SnO_2_ bilayer enhances the surface passivation, suppresses nonradiative recombination, and helps optimize band alignment, improving electron extraction and blocking hole backflow from the perovskite. Recently, Moradi’s group achieved a record PCE of 27.20% with a FF of 86.28% by using a combination of V_2_O_5_ and ZnO/AZO bilayer in PSCs.^207^ The ZnO/AZO bilayer produced an ultrathin ET layer, which promoted better electron transport and reduced recombination losses. Additionally, the V_2_O_5_ HTL features a deep valence band, enabling efficient hole extraction, blocking electron backflow, and minimizing recombination losses, resulting in thermally stable and moisture-resistant properties, and offering longevity and stability to the PSC.

**Table 4:** List of Inorganic Oxides and Salts in PSCs and the Corresponding PCE and FF Values

perovskite	ligand	PCE (%)	FF (%)	ref
FA_0.85_MA_0.15_-Pb(I_*x*_Br_1–*x*_)_3_	ZnO–SnO_2_	15.1 (only ZnO), 16.5 (only SnO_2_) to 18.1	64.8, 70.9–71.6	^203^
(MA)PbI_3_	ZnO, SnO_2_	21.1, 20.3	NA	^204^
MAPb(I_*x*_Br_1–*x*_)_3_	Al-doped ZnO films	17.20–18.45	79– 78	^205^
CH_3_NH_3_PbI_3_	PC_60_BM, Al:ZnO, triphenyl-phosphine oxide (TPPO)	14.6–19.2	69–82	^206^
Cs_2_BiAgI_6_-double perovskite	V_2_O_5_, ZnO/AZO	21.675–27.20	82.78–86.28	^207^
Cs_0.05_(FA_0.85_MA_0.15_)_0.95_-Pb(I_0.85_Br_0.15_)_3_	KI	20.58	0.79	^208^
(Cs_0.05_FA_0.54_MA_0.41_)-Pb(I_0.98_Br_0.02_)_3_	NaF	21.99	80.35	^209^
FAPbI_3_	NbF_5_	20.56	77.08	^210^
FA_0.85_MA_0.15_PbI_3_	cesium acetate	21.95	78.64	^211^
CH_3_NH_3_PbI_3_	NiCl_2_	20.6	81	^212^
FA_0.85_MA_0.15_-PbI_2.55_Br_0.45_	KI	17.55	72.5	^213^
(Cs_0.06_MA_0.15_FA_0.79_)-Pb(I_0.85_Br_0.15_)_3_	RbI	20.6	78	^214^
(FAPbI_3_)_0.98_-(MAPbBr_3_)_0.02_	CsI-passivated	24.1	82.2	^215^

Furthermore, inorganic salts also serve as effective passivators in PSCs, enabling defect passivation, phase stabilization, and long-term operational stability. They include alkali, alkaline earth metals, and transition metal salts,^208–215^ which fills surface traps to suppress nonradiative recombination and improve structural integrity. Some of the PSCs made using these inorganic salts, their corresponding PCEs, and FFs are provided in Table 4.

For example, Li et al. reported that the addition of NaF onto a perovskite surface prevents the movement of both A-site cations and anions.^209^ The fluoride ions interact with cations (Pb^2+^ and A-site) as well as fill halide anion vacancies, forming ionic bonds like Pb–F and strong hydrogen bonding with organic cations, thereby suppressing ion migration, improving surface passivation, and increasing the stability. The corresponding PSC exhibited 21.46% PCE and 80.35% FF. Figures 5a-c shows a scheme of defect passivation by NaF, and the PSC’s *I*–*V* characteristics and the PCE over time. In another study, Yuan et al. demonstrated that the addition of NbF_5_ prevents the phase transition of FAPbI_3_ from the photoactive black α-phase (cubic) into the nonphotoactive yellow δ-phase (hexagonal).^210^ In this case, Nb^5+^ acts as a Lewis acid and stabilizes the PbI_6_ octahedra, whereas F^–^ creates strong Pb–F bonds, thereby suppressing ion migration, passivating grain boundaries, enhancing stability, and yielding a PCE of 20.56% with a FF of 77.08%. Recently, Baumeler et al. achieved an improved PCE of 24.1% with a FF of 82.2% by treating FAPbI_3_ PSCs with CsI.^215^ They found that the CsI forms a uniform thin layer that passivates cationic and anionic defects, minimizes lattice distortion, and lowers the work function. The device retained ∼ 90% of its initial PCE after 600 h of continuous sunlight illumination. Figures 5d,e compares the *I*–*V* characteristics and normalized PCE for the fabricated device with and without CsI passivation.

**Figure 5:**
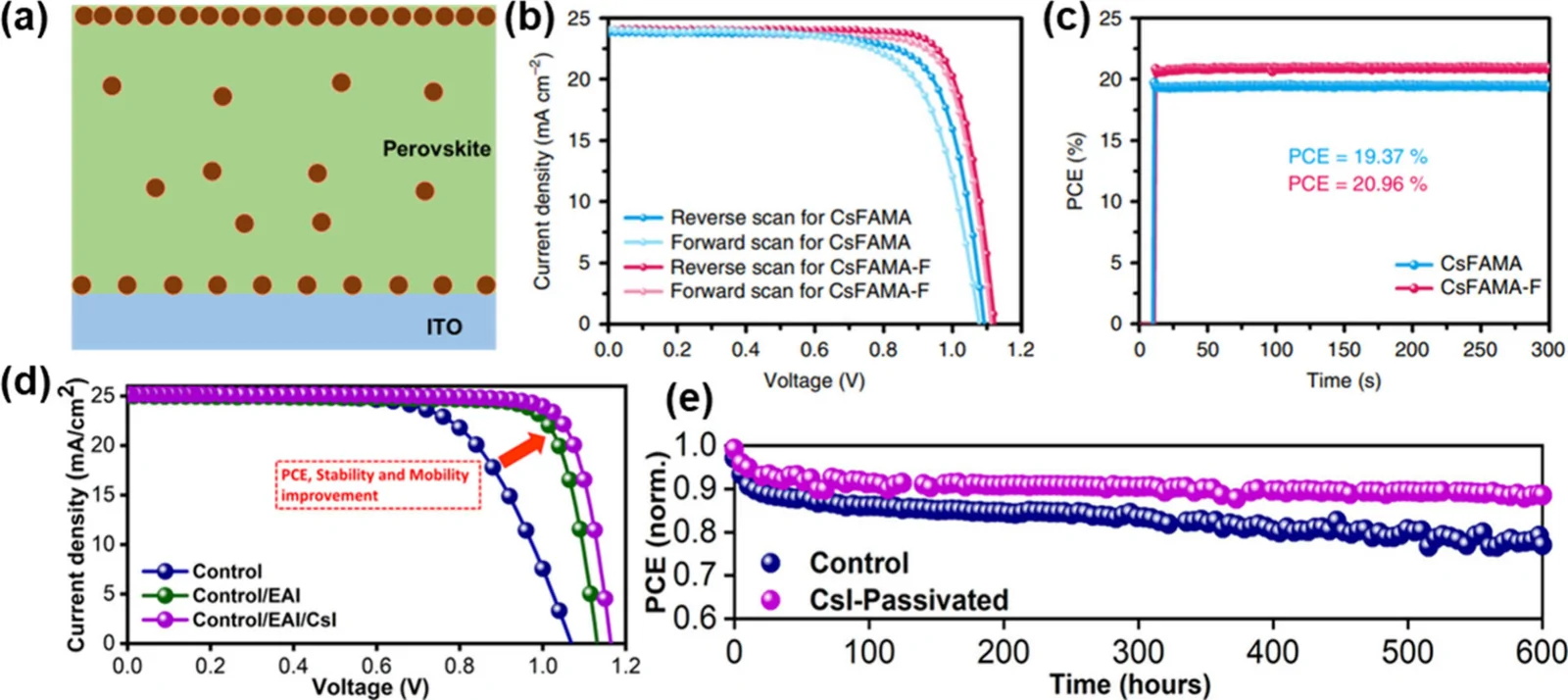
(a) Scheme demonstrating the movement of NaF in the perovskite layer, (b) *I*–*V* and (c) PCE characteristics of the corresponding PSC with and without NaF incorporation, and (d) *I*–*V* and (e) normalized PCE characteristics of a PSC with CsI-passivated FAPbI_3_ perovskite/TiO_2_ interface. (b, c) Reproduced with permission from ref 209. Copyright 2019 Springer Nature. (d, e) Reproduced with permission from ref 215. Copyright 2023 American Chemical Society.

Passivation of defects, either through the introduction of passivating agents, such as small molecules, Lewis acid/base agents, or polymeric passivators, together with the precursors or via post-crystallization treatment, is essential to achieve relatively high efficiency and stability (both long-term and operational) in perovskite LEDs and solar cells.

While the above organic or inorganic molecules or salts offer mostly monofunctional passivation, several bi- and multifunctional molecules offer bi- or multimodal passivation modes. We collected several such multifunctional molecules in Table 5, which provide prominent PCE and FF enhancements via the passivation of defects and suppression of grain boundaries. These molecules include, but are not limited to, alcohols, carboxylic acids, thiols, imides, silanes, thiophenes, sulfonates, hydrazines, phosphates, phosphonates, and Europium complexes.^216–228^

**Table 5:** List of Miscellaneous Examples of Ligands (Nonfullerene-Type) in PSCs and the Corresponding PCE and FF Values

perovskite	ligand	PCE (%)	FF (%)	ref
FA_0.83_Cs_0.17_-Pb(I_0.85_Br_0.15_)_3_	3-aminopropyl-trimethoxysilane (APTMS)	18.03 ± 0.66	77.78 ± 1.93	^216^
α-(FAPbI_3_)_1–*x*_-(MAPbBr_3_)_*x*_	potassium bis(fluorosulfonyl)-imide (KFSI)	18.95	71.85	^217^
FA_0.85_MA_0.15_PbI_3_	indaceno[1,2-*b*:5,6-*b*’]-dithiophene (IDT)-oxyindole	24.04	81.38	^218^
Rb_0.02_(FA_0.95_Cs_0.05_)_0.98_-PbI_2.91_Br_0.03_Cl_0.06_	(5-mercapto-1,3,4-thiadiazol-2-ylthio)acetic acid (MTDAA)	21.92	82.2	^219^
(FAPbI_3_)_0.875_-(MAPbBr_3_)_0.075_-(CsPbI_3_)_0.05_(PbI_2_)_0.03_	2,7-dioctyl[1]-benzothieno[3,2-*b*][1]-benzothiophene (C8-BTBT)	21.94	80.13	^220^
(FA_0.8_MA_0.2_-Pb(I_0.8_Br_0.2_)_3_)	(5*Z*,5′*Z*)-5,5′-((7,7′-(3,3′-dioctyl-[2,2′-bithiophene]-5,5′-diyl)bis(benzo[*c*][1,2,5]-thiadiazole-7,4-diyl))-bis(methanylylidene))-bis(3-ethyl-2-thioxothiazolidin-4-one) (Y-Th2)	21.5	79.5	^221^
FA_0.81_MA_0.14_-Cs_0.05_PbI_2.55_Br_0.45_	indacenodithieno[3,2-*b*] thiophene (IDTT)	21.2	75.2	^222^
CH_3_NH_3_-PbI_3_(MAPbI_3_)	europium acetylacetonate [Eu(acac)_3_]	21.89	80.7	^223^
plate-like 2D (Eu-pyP)_0.5_MA_*n*–1_-Pb_*n*_I_3*n*+1_/3D MAPbI_3_	Eu-porphyrin complex (Eu-pyP)	18.35	74.92	^224^
FA_0.9_Cs_0.1_PbI_3_	P-biguanylbenzoic acid hydrochloride (PBGH)	24.79	82.76	^225^
Cs_0.05_(FA_0.98_MA_0.02_)_0.95_-Pb(I_0.98_Br_0.02_)_3_	PY-IT	23.57	84	^226^
CsPbI_3–*x*_Br_*x*_	Boc-S-4-methoxy-benzyl-l-cysteine (BMBC)	21.75	83.57	^227^
(FAPbI_3_)_0.98_-(MAPbBr_3_)_0.02_	4-chlorophenyl-trifluoroborate potassium salt (4-ClPTFBK)	24.50	84.3	^228^

## Metal Halide Perovskite (Bulk and Nanocrystals) LEDs

Metal halide perovskites have emerged as exceptional materials for LEDs due to their unique combination of optical, electronic, and structural properties. One of their most notable features is their near-unity PLQYs, which are achieved even without a wide-bandgap shell and despite being synthesized from technical-grade precursors. Further, their emission color can be precisely tuned across the visible spectrum, from blue to green to red, simply by varying the halide ion composition (Cl, Br, and I). This tunability, coupled with their narrow emission line width (Full width half-maximum (fwhm) of ∼ 12–30 nm) and defect-tolerant electronic structure, makes them ideal for next-generation LEDs, especially in color-pure display and lasing applications. Their straightforward, low-temperature, solution-based synthesis further enhances their appeal, enabling cost-effective and scalable fabrication.

Moreover, the emission of MHPs is not limited to the visible spectrum; instead, it extends into the near-infrared (NIR) region, exhibiting both narrow- and broad-bandwidth radiation.^229–231^ These developments mainly rely on several strategies, including tin-based and mixed Pb–Sn halide perovskites (e.g., CsSnI_3_, CH_3_NH_3_(Pb_1–*x*_Sn_*x*_)­I_3_),^229,232,233^ lanthanide doping (e.g., CsPbCl_3_:Yb^3+^),^231,234^ zero-dimensional heavy-metal-based perovskites (e.g., Cs_2_MoCl_6_, Cs_2_WCl_6_),^235,236^ as well as defect- and low-dimensionality-induced near-infrared emission.^237^ In these materials, NIR emission is governed by multiple mechanisms, including intrinsic bandgap narrowing in tin-based perovskites, sharp *f*–*f* transitions of lanthanide dopants, and broadband emission from self-trapped excitons resulting from strong electron–phonon coupling in heavy-MHPs. Thus, these broad characteristics, together, position MHPs as highly promising candidates for advanced optoelectronic applications.

In a typical perovskite-based LED (PeLED), as illustrated in Figure 6a, charge carriers are injected via the electrodes: electrons travel through the electron injection layer (EIL) and ETL. At the same time, holes move through the hole injection layer (HIL) and HTL. These carriers then recombine within the perovskite emissive layer, generating electroluminescence (EL). Building on this mechanism, Figure 6b shows the energy-level alignment of representative HTLs and ETLs with red, green, and blue emissive MHPs. As the VBM deepens from red to blue, the hole-injection barrier increases due to the limited availability of HTLs with sufficiently high HOMO levels. This causes hole buildup at the HTL/emissive layer interface, leading to a charge imbalance in the MHP layer and ultimately reducing the efficiency. In contrast, CBM aligns well with a broad range of commonly used ETLs.^238,239^

**Figure 6:**
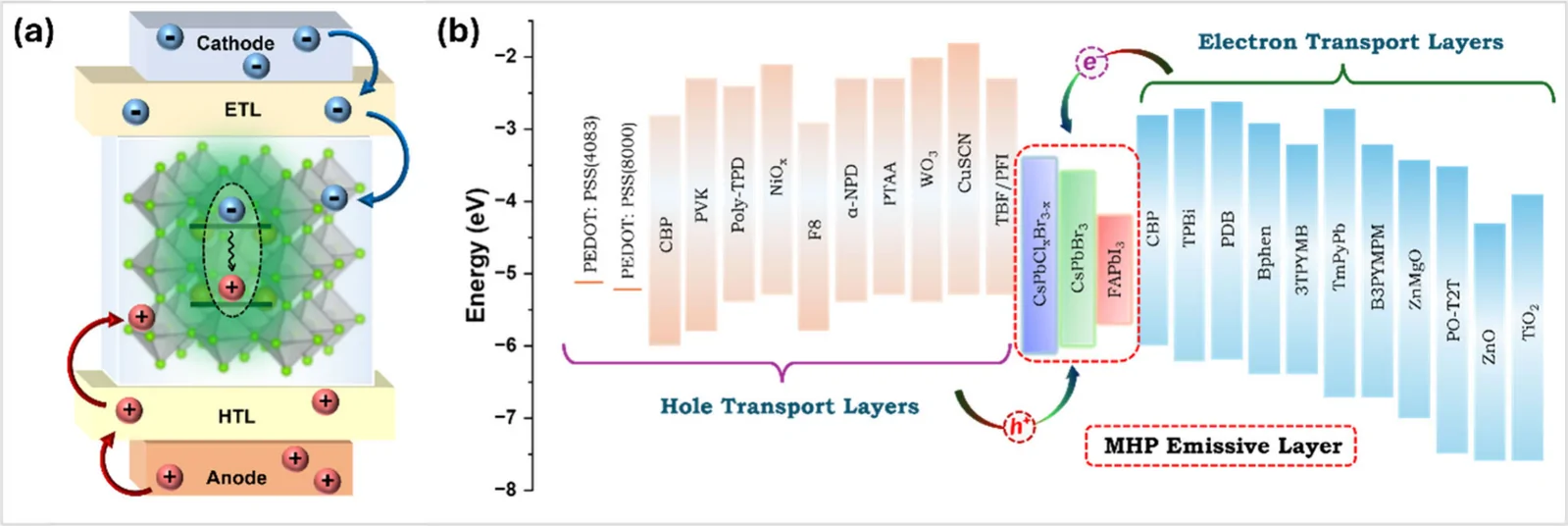
(a) Schematic illustration of LED operation. (b) Energy-level alignment of representative hole-transport and electron-transport layers relative to blue (CsPbCl_*x*_Br_3–*x*_), green (CsPbBr_3_), and red (FAPbI_3_) emissive metal halide perovskites.^239^

For the commercial viability of PeLEDs, two key performance metrics are critical: (1) EQE and (2) operational lifetime (*T*_50_). Here, the EQE can be expressed as follows:^240^

10 $$\mathrm{EQE}=\eta_{\mathrm{out}}\cdot \mathrm{IQE}$$

where η_out_ represents the out-coupling factor, and IQE denotes the internal quantum efficiency. The η_out_ primarily depends on the device architecture rather than the intrinsic material properties. In contrast, IQE is defined as

11 $$\mathrm{IQE}=\eta_{\mathrm{in}}\cdot \eta_{r}$$

where η_in_ refers to the ratio of the current associated with radiative recombination to the total injected current, while η_r_ represents the radiative efficiency, defined as the ratio of radiative recombination to total recombination (radiative and nonradiative).^17^ Therefore, achieving high radiative efficiency, inherently associated with balanced injection of electrons and holes, is essential for efficient LED operation.

On the other hand, the operational lifetime (*T*_50_) refers to the time it takes for the device’s luminance to degrade to 50% of its initial value, serving as an indicator of device stability.^241^ Stability testing for PeLED is typically performed under a constant driving current, with the initial luminance set at 100 cd·m^–2^.^241^ This standardized condition ensures a fair and consistent basis for comparing the device performance.

### Key Factors in Optimizing LED Performance

Over the past decade, PeLEDs have undergone significant advances, enabling record-breaking efficiencies in LED technologies.^242^ The best-performing perovskite NC LEDs have achieved impressive EQEs, exceeding 30% for green emission^243^ and reaching 28.5% for pure-red emission.^244^ These figures are approaching the highest efficiencies reported for perovskite bulk thin film-based LEDs, which have recently set benchmarks with EQEs of 32.1% for green and 32.14% for red emission.^245,246^

To achieve maximum EQE in LEDs, four critical factors must be considered: (1) Charge Carrier Balance: A balanced injection of electrons and holes into the emissive layer should be ensured, ideally with a 1:1 ratio, to promote efficient radiative recombination. (2) Minimization of the Leakage Current: The flow of charge carriers that traverse the device without forming excitons should be suppressed, thereby reducing energy loss due to unproductive charge transport. (3) Suppression of Nonradiative Recombination: Nonradiative recombination pathways (e.g., defect-related traps) should be minimized to ensure that charge recombination results in photon emission. (4) Enhanced Light Extraction (Outcoupling): The outcoupling efficiency by improving the extraction of internally generated photons into the external environment should be maximized.

PeLEDs still face significant challenges when compared with II–VI and III–V colloidal quantum dots (QDs) that are already in extensive use in real world devices. Recently, solution-processed colloidal QD-based LEDs (QDLEDs) have demonstrated an exceptional operational lifetime (*T*_50_) of 640 h at an initial luminance of 22270 cd m^–2^ which is equivalent to 18000000 h at a luminance of 100 cd m^–2^; however, the corresponding EQE remains limited to approximately 13%.^247^ This highlights a key trade-off in QDLED development. Although remarkable stability has been achieved, the EQE still lags behind that of state-of-the-art PeLEDs.^248^ The superior operational stability of QDLEDs largely originates from the intrinsic covalent bonding within II–VI and III–V semiconductor lattices, which effectively suppresses ion migration, a degradation pathway that critically limits the operational lifetime of PeLEDs. In contrast, MHP benefits from an outstanding defect tolerance property, allowing them to achieve record-high EQEs. However, their ionic nature renders them vulnerable to ion migration, phase segregation, and interfacial instability under electrical bias. Therefore, rather than viewing colloidal QDs and MHPs as competing platforms, their complementary strengths point toward a promising convergence. With appropriate materials and interfacial engineering, particularly through effective surface passivation of MHPs, PeLEDs hold great promise to simultaneously achieve a high EQE and appreciable operational lifetimes.

Though, MHP is not fully extricated from defects. During the colloidal synthesis of NCs, washing of colloidal NCs or NC-deposited substrates with mildly polar solvents may exacerbate the problem by stripping excess surface ligands. We note that the dynamic binding behavior of ligands, typically oleic acid and oleylamine, often results in incomplete surface coverage, leaving portions of the perovskite NC surface exposed as well as leads to the formation of surface vacancies.^249,250^ Additionally, the surface halide vacancies can promote ion migration, leading to severe phase segregation in mixed-halide perovskites, and affecting the stability of the EL peak under constant-bias conditions.^251^ Moreover, during device operation, joule heating can weaken ligand binding, accelerating surface degradation. These surface-related processes collectively reduce the efficiency loss in PeLED devices.

Following facile solution-processed synthesis, MHP typically crystallizes into thin films with surface terminations predominantly consisting of AX and BX_2_ facets.^252^ As discussed in above sections, vacancies at the A-site and X-site are particularly common due to their low formation energies compared to other defects.^252^ These surface vacancies primarily act as trap states, significantly hindering the radiative recombination of excitons during PeLED operation. To mitigate these issues, effective surface passivation is critical. By stabilizing the MHP surface and minimizing defect sites, surface passivation enhances carrier confinement, reduces nonradiative recombination pathways, and significantly improves both the EQE and operational lifetime of PeLEDs. In subsequent sections, we will discuss various surface passivation strategies for MHP thin films and NCs separately that have played a pivotal role in advancing the development of PeLEDs with improved efficiency and stability.

### Thin Film/Bulk-Based PeLED

This section discusses strategies aimed at mitigating defects and improving EQE in thin films made of bulk and low-dimensional MHPs, covering both pre- and postfabrication stages. These approaches include engineering charge transport layers, passivation via ionic and acid–base interactions, and phase engineering. Collectively, these strategies help balance charge transport, reduce surface defects, enable efficient energy funneling, and promote uniform phase distribution. (Figure 7)

**Figure 7:**
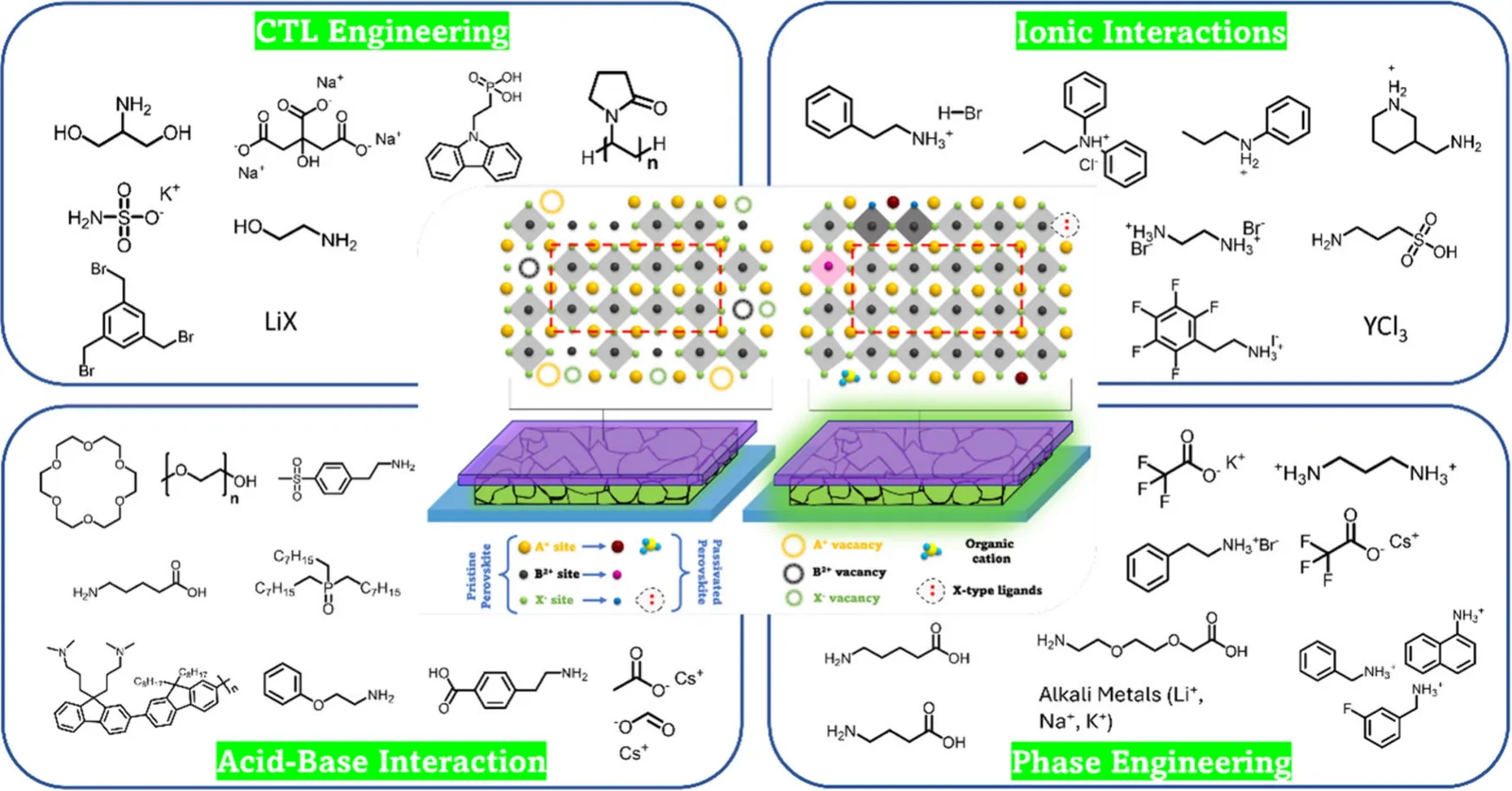
Summary of the passivating molecules discussed in this section, highlighting their potential mechanisms: charge transporting layer (CTL) engineering, ionic interactions, acid–base interactions, and phase engineering. References can be found in the tables provided in the various sections.

#### Charge Transporting Layer Engineering

The sequential deposition of multilayers during device fabrication results in the formation of a high density of interfacial defects including the interface between the CTL and perovskites. The deposited MHPs over CTL consist of a significant number of dangling bonds, pinholes, larger grain boundaries, etc., ultimately enhancing the nonradiative losses. Thus, CTL engineering before the MHPs fabrication is essential for boosting LEDs’ performance. Addressing this, a lithium fluoride (LiF) layer has been deposited on top of an organic semiconductor HTL (TFB) before MHPs deposition for a visible LED.^253^ LiF acted as a high dielectric template for MHPs fabrication, which was otherwise incompatible directly on a hydrophobic organic HTL. This resulted in larger and well-defined grains with significantly reduced pinhole density. In another work, the researchers have found the direct deposition of perovskite onto PEDOT: PSS HTL resulted in uneven hole transport, while introducing 2-amino-1,3-propanediol at the interface promoted smaller grain sizes, resulting in smoother hole transport and significantly improved device performance.^254^ Likewise, it was observed that hole injection through the NiO_*x*_/poly­(9-vinylcarbazole) (PVK) interface was less efficient than electron injection through ETL.^255^ To improve charge imbalance, the researchers incorporated a self-assembled monolayer (SAM) of [2-(9*H*-carbazol-9-yl)­ethyl]­phosphonic acid (2PACz), thereby modifying the hole transport structure to NiO_*x*_/2PACz/PVK (Figure 8a). The modified layer exhibited improved energy level alignment between NiO_*x*_ and PVK, leading to better charge balance, along with effective passivation at the NiO_*x*_ interface (Figure 8b). The smart design of the SAM, featuring a phosphonic acid group and a carbazole moiety, served dual functions: the phosphonic acid group anchored to NiO_*x*_ via tridentate binding (covalent interaction), while the carbazole moiety interacted strongly with PVK due to its structural similarity (van der Waals interaction) (Figure 8a). The deposited perovskite on a robust trilayer HTL exhibited the highest PLQY (85%) when compared with control HTLs: NiO_*x*_/PVK (13.9%) and NiO_*x*_ (6.5%). The incorporation of the SAM-modified layer resulted in enhanced EQE in both quasi-2D perovskite-based green and blue LEDs. Notably, the EQE of blue LEDs increased from 8.9% to 14.5% (Figure 8c), while that of green LEDs improved from 21.6% to 26.0% (Figure 8d).

**Figure 8:**
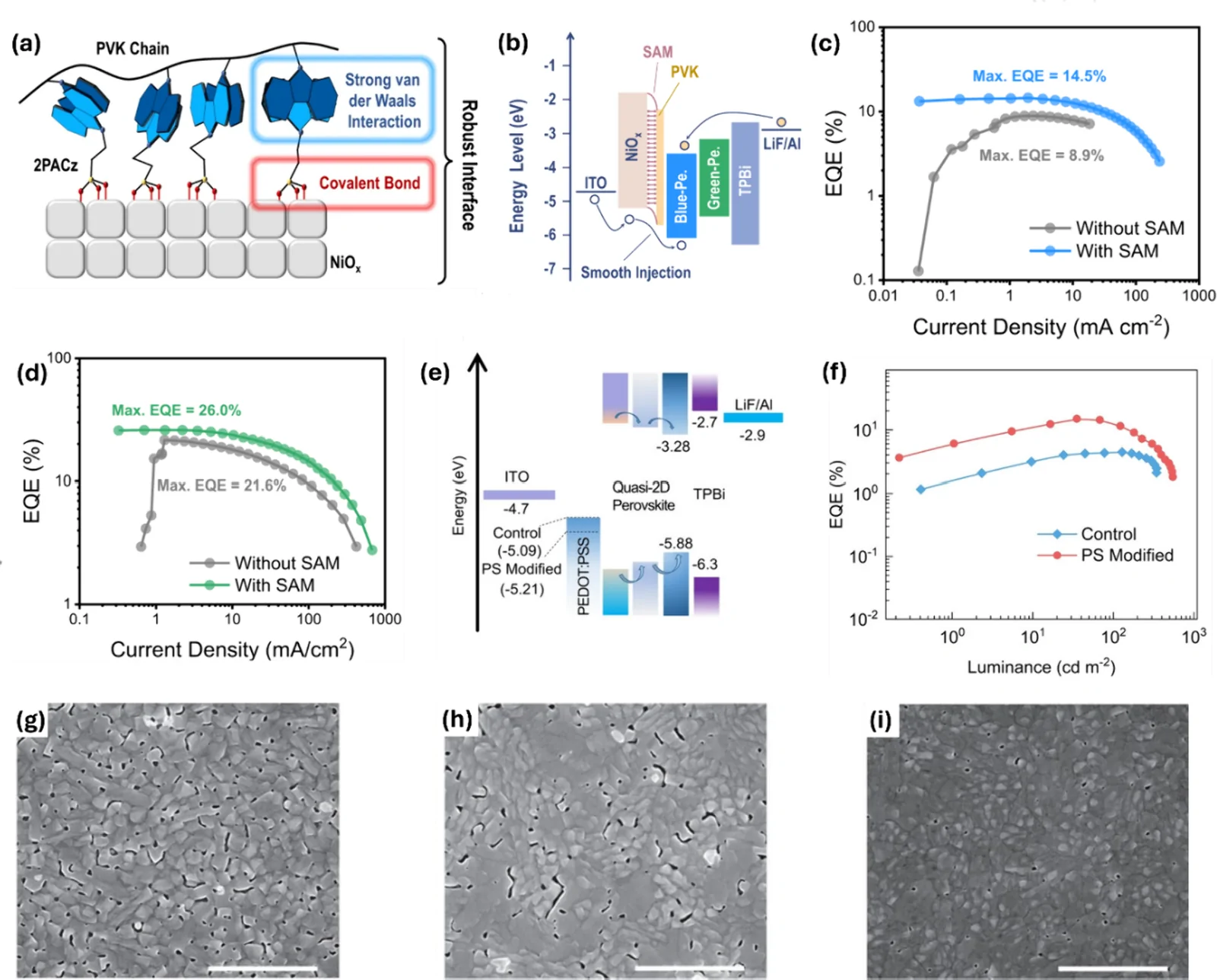
(a) Schematic illustration highlighting the enhanced robustness of the NiO_*x*_/2PACz/PVK HTL structure, attributed to potential covalent bonding between PACz and NiO_*x*_, and van der Waals interactions with PVK. (b) Energy level alignment of the various components in green and blue PeLEDs incorporating a self-assembled monolayer (SAM) of 2PACz. (c, d) EQE versus current density characteristics of blue (c) and green (d) PeLEDs, comparing devices with and without the 2PACz SAM. Panels (a)–(d) were reproduced from ref 255. Available under a CC-BY 4.0. Copyright 2023 The Author(s). Published by Springer Nature. (e) Modification of the flat band energy diagram upon treatment with zwitterionic potassium sulfamate (PS), indicating improved hole injection in the treated PeLED. (f) EQE vs luminance plots for PeLEDs with and without PS treatment. Panels (e) and (f) were reproduced with permission from ref 256. Copyright 2024 Wiley-VCH GmbH. (g–i) SEM images showing CsPbBr_3_ deposited directly on ZnO with prominent pinholes (g), improved film morphology when deposited on PVP-modified ZnO (h), and a smooth, defect-free surface achieved by incorporating CH_3_NH_3_Br in the precursor and depositing on PVP/ZnO (i). Panels (g)–(i) reproduced from ref 69. Available under a CC-BY 4.0. Copyright 2017 The Author(s). Published by Springer Nature.

In another approach, the incorporation of the zwitterionic additive potassium sulfamate (PS) into the HTL PEDOT:PSS improves hole injection, the phase distribution of quasi-2D perovskites, and effectively passivates trap states, resulting in an EQE of up to 17.32% with blue emission at 478 nm.^256^ XPS and EDS analyses confirmed the uniform distribution of potassium on the HTL, where it acts as a heterogeneous nucleation seed to regulate the growth of perovskite grains. Meanwhile, the sulfamate group can bind to undercoordinated lead sites, simultaneously regulating the phase distribution and passivating halide vacancies. In addition, the amine group facilitates hydrogen bonding with halides, thereby suppressing halide migration. Therefore, ultraviolet photoelectron spectroscopy (UPS) was employed to investigate the energy level changes in PEDOT: PSS and perovskites after PS incorporation. The HOMO level of PEDOT: PSS shifted to a deeper position, while the VBM of the perovskites shifted to a shallower level (Figure 8e). This improved energy level alignment facilitated more efficient hole injection from the anode into the perovskite layer, resulting in better charge balance and, consequently, reduced turn-on voltage and enhanced EQE (Figure 8f). Overall, this work highlights the potential of molecular-level interface engineering to synergistically optimize crystallization, defect passivation, and energy level alignment, paving the way for high-efficiency and stable perovskite-based optoelectronic devices. Table 6 lists several additives that helped improve the charge injection, quality of the film, and, consequently, the LED performance.

**Table 6:**
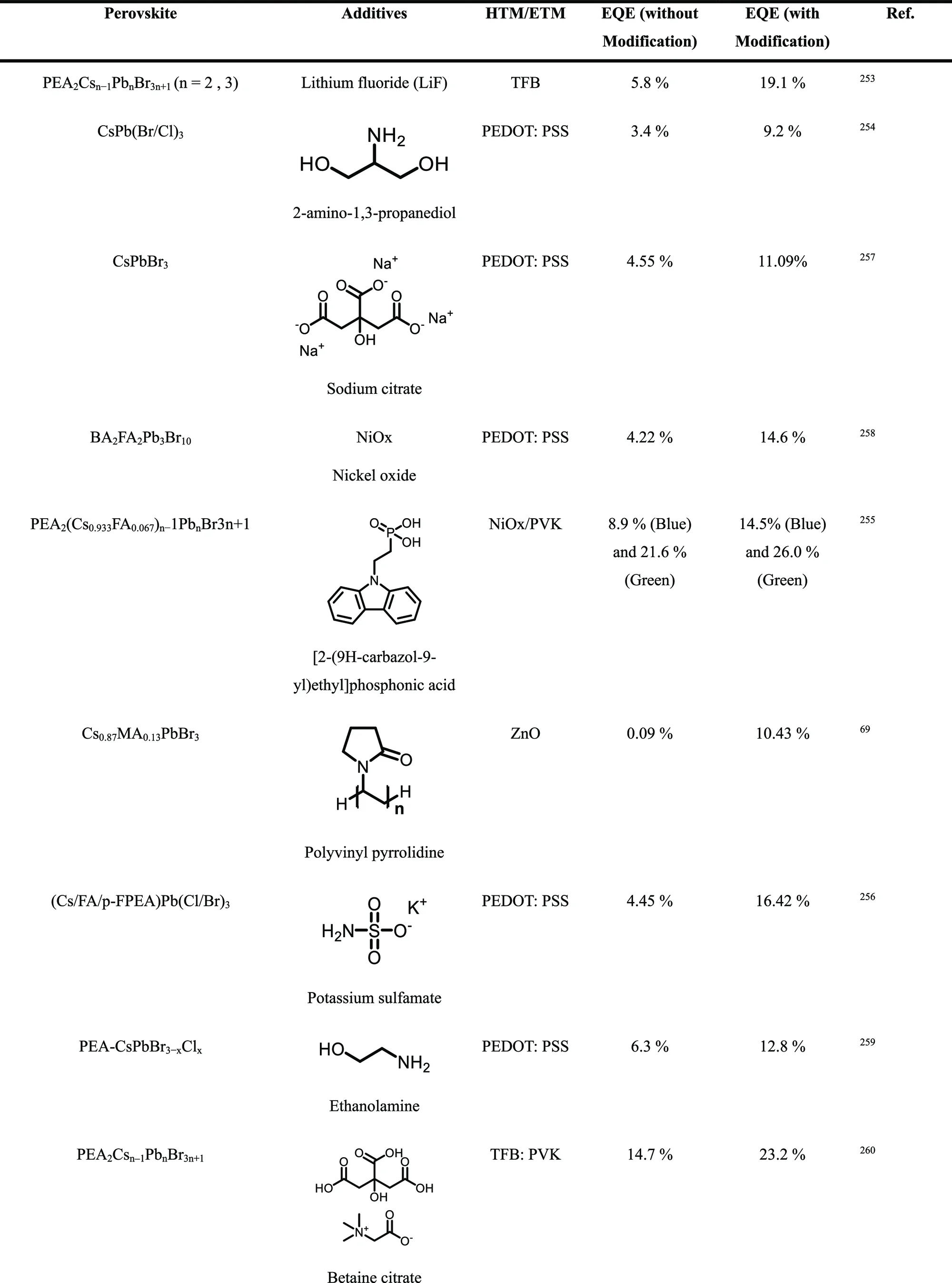
Additives for Modifying HTMs and ETMs to Enhance Charge Injection and Perovskite Film Passivation^69,253,254,255,256,257,258,259,260^

On the other hand, direct deposition of MHPs onto the ETL also results in degraded LED performance, similar to the effects discussed for HTLs. For instance, the use of ZnO as an ETL in PeLEDs results in the formation of a high density of pinholes within the perovskite layer (Figure 8g). To address this issue, a thin layer of polyvinylpyrrolidone (PVP), an insulating and hydrophilic polymer, was introduced between ZnO and CsPbBr_3_, leading to a significant increase in the PL intensity.^69^ The improved surface wettability resulting from the PVP modification facilitated a better interaction with the perovskite precursor solution, ultimately forming a more uniform perovskite film with significantly lower pinholes (Figure 8h). A subsequent treatment with MABr modulated the growth kinetics, leading to a smoother surface morphology (Figure 8i) and enhanced PL, ultimately achieving an EQE of 10.43% with an emission at 520 nm.

The strategies discussed above highlight the critical role of CTL surface modification with various additives in regulating nucleation kinetics, improving interfacial energy level alignment and minimizing defect densities at the perovskite/CTL interface. Such interfacial engineering not only promotes balanced charge transport but also enables reduced nonradiative recombination and uniform perovskite crystallization. Building on this foundation, we explore various passivators for MHP films in LEDs to mitigate defect states and enhance operational stability and efficiency.

#### Electrostatic Passivation of MHPs

Given the ionic nature of MHPs, the ionic interactions between the perovskite structure and passivator molecules play a crucial role in reducing trap states and thus enhancing PLQY/EQE, as summarized in Table 7. The typical organic ammonium ligands are not only known for stabilizing nanocrystals and controlling the dimensionality of perovskite structures but also serve effectively as passivating agents. Among them, phenethylammonium bromide (PEABr) was realized to be an effective passivator molecule for MHPs. For instance, PEABr has been used to passivate quasi-2D perovskites, leading to a ∼ 60% reduction in trap density and improving the EQE from 6.15% to 7.51%.^261^ In case of mixed halide perovkites, the traps can accelerate the halide migration, which poses a significant challenge to the operational stability of blue and red LEDs.^262^ To address this, organic cation diphenylpropylammonium chloride has been used to passivate chloride vacancies in CsPbBr_2_Cl, resulting in enhanced operational stability and an EQE of 3.03%.^263^ Similarly, phenyl alkyl ammonium iodide with varying chain length has been tested and found that longer chains promote more robust binding and steric hindrance on FA_0.83_Cs_0.17_PbI_3_ film, leading to enhanced stabilization of the lattice via inhibition of halide migration.^264^ Through the optimization of different chain lengths, a maximum EQE of 17.5% was achieved for phenyl propyl ammonium iodide with a remarkable *T*_50_ of 130 h. Likewise, Liang and co-workers synthesized a series of ammonium-, formamidinium-, and imidazolium-functionalized molecules based on phenyl and thienyl backbones to explore their potential in passivation of MHPs (Figure 9a). The incorporation of these molecules into 3D MAPbI_3_ perovskite films resulted in a reduction in the defect density, surface roughness, and grain size. The surface characterization using X-ray scattering revealed that the formation of low-dimensional perovskite phases on 3D-perovskites was responsible for the observed improvements in optoelectronic properties. Among the tested passivating molecules (Figure 9b), the imidazolium-based compound (PImI) yielded the highest LED performance, achieving an EQE of 15.6%.^265^

**Figure 9:**
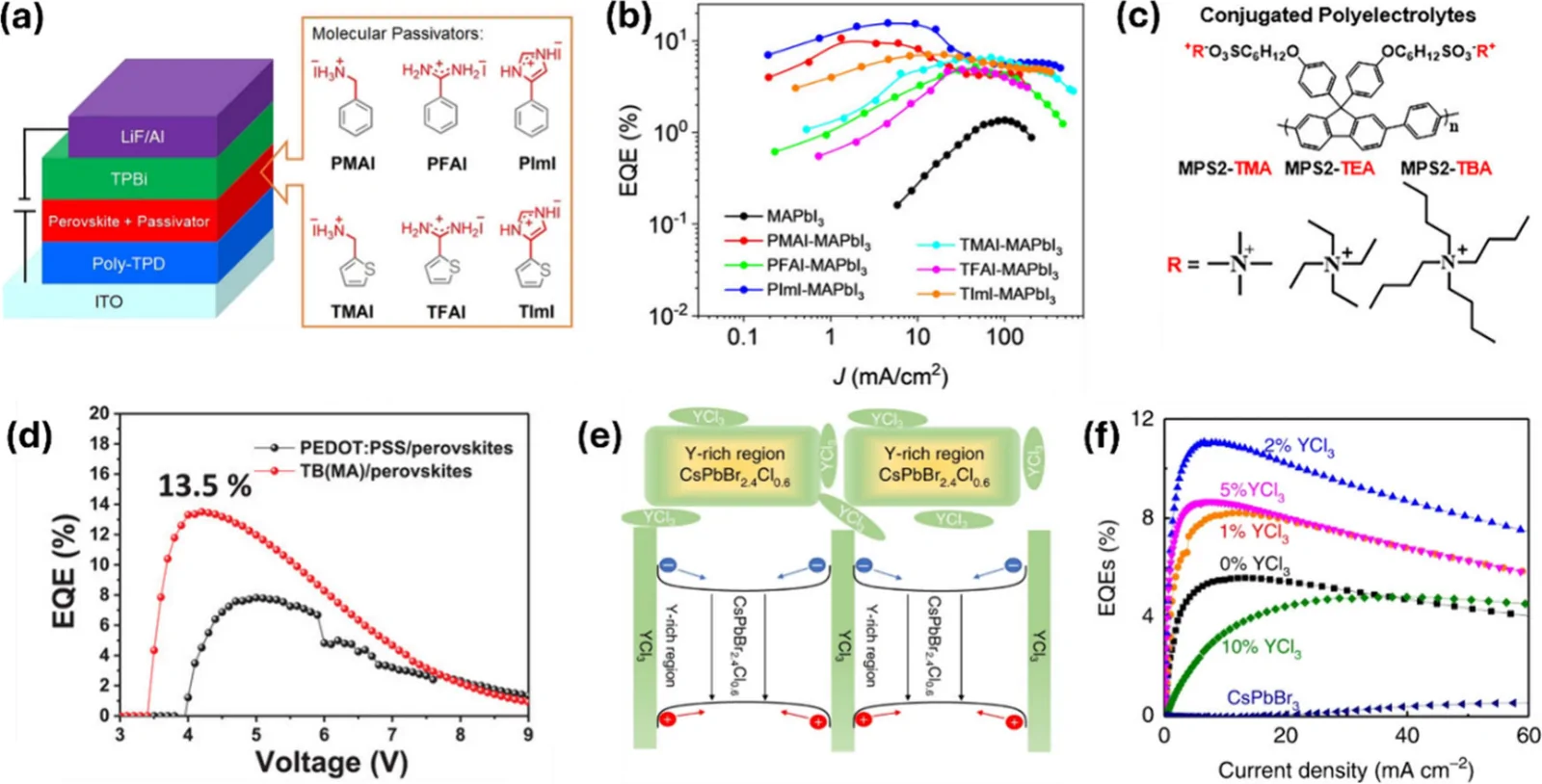
(a) Schematic representation of MAPbI_3_-based perovskite LEDs passivated with various nitrogen-containing molecules featuring different anchoring groups: methylammonium (MA^+^), formamidinium (FA^+^), and imidazolium (Im^+^). (b) EQE vs current density plots for LEDs incorporating the passivation molecules shown in (a). Panels (a) and (b) were reproduced with permission from ref 265. Copyright 2021 Wiley-VCH GmbH­(c). Structures of anionic conjugated polyelectrolytes (CPEs) with different counterions used for perovskite surface passivation. Reproduced with permission from ref 266. Copyright 2020 American Chemical Society. (d) EQE versus voltage characteristics of perovskite LEDs employing PEDOT: PSS and TB­(MA) were used as hole transport layers. Reproduced with permission from ref 267. Copyright 2021 Wiley-VCH GmbH. (e) Schematic of YCl_3_-treated perovskite grains, showing Y-rich boundaries that spatially confine charge carriers, enhancing radiative recombination. (f) EQE versus current density plots comparing pristine and YCl_3_-treated perovskite LEDs. Panels (e) and (f) were reproduced from ref 268. Available under a CC-BY 4.0. Copyright 2019 The Author(s). Published by Springer Nature.

**Table 7:**
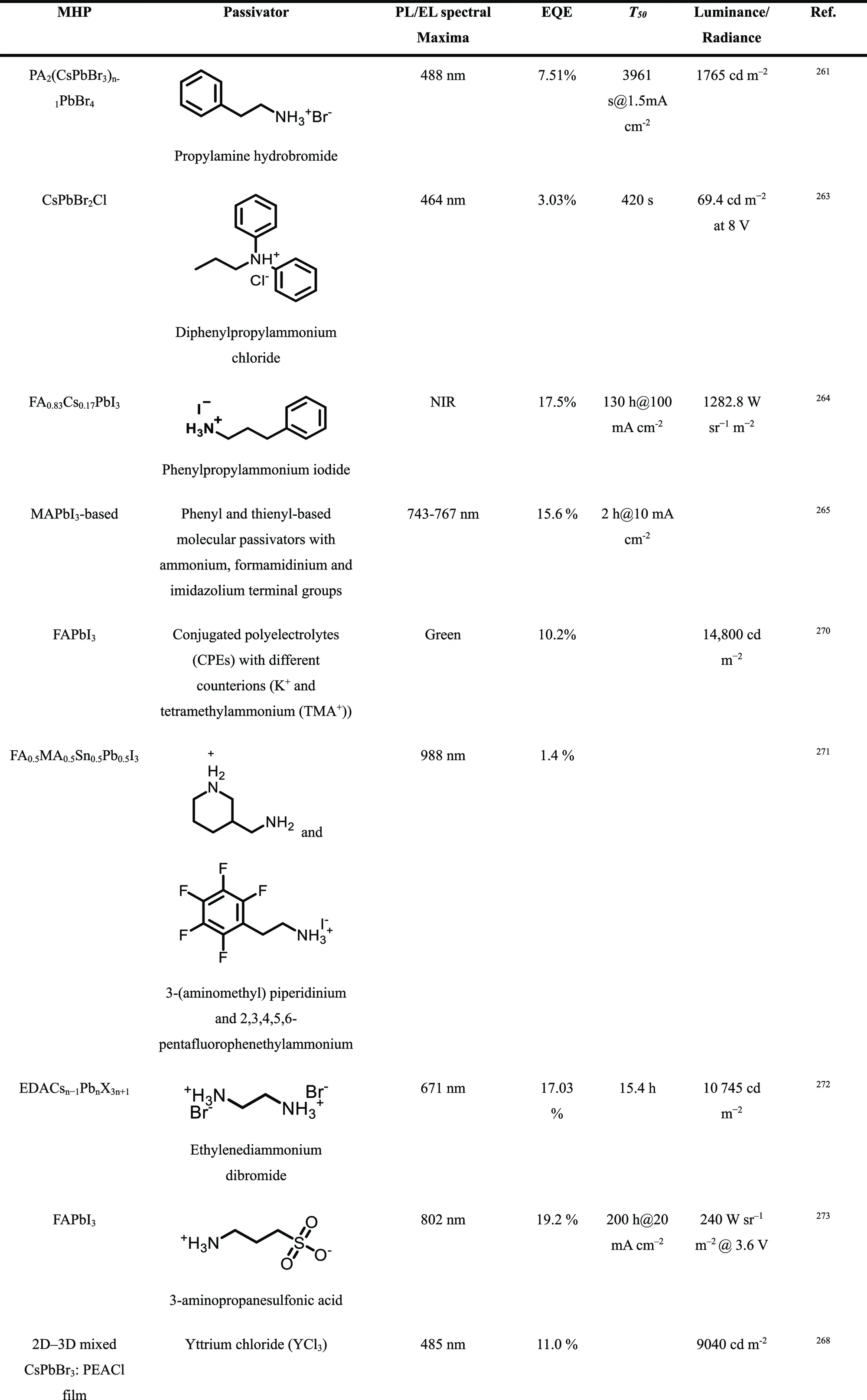
List of Passivating Molecules and Ions That Passivate Perovskite Films via Ionic Interactions^261,263,264,265,268,270,271,272,273^

Furthermore, conjugated polyelectrolytes (CPEs) with various counterions have also been investigated as HTLs to enhance the performance of PeLEDs. Primarily, CPEs promote the growth of highly crystalline MHP layers and improve the surface wettability, collectively leading to enhanced optoelectronic properties. In addition, an appropriate choice of counterions helps in passivating interfacial defects.^266,269^ For example, poly­(fluorene-phenylene)-based CPEs were utilized with potassium and tetramethylammonium cations. Notably, the ammonium cations demonstrated strong passivation, achieving a maximum EQE of 10.2%.^270^ In another report, Jang et al. tested the effect of size of the alkyl chain (*R* = C_*n*_H_2__*n*+1_) in tetraalkylammonium counterions in passivation of MHPs and found that the larger-sized ion (*n* = 4) led to more effective passivation and improved crystalline growth of the perovskite (Figure 9c).^266^ Similarly, CPE with MA^+^ (CH_3_NH_3_^+^) counterion, termed TB­(MA), was tested as a hole-transporting layer in place of the commonly used acidic PEDOT: PSS. The hydrophilic nature of TB­(MA) facilitated the formation of a high-quality perovskite layer and enabled interfacial defect passivation via MA^+^, leading to an EQE of up to 13.5% (Figure 9d).^267^

Despite having the ability to passivate MHPs effectively, organic cations pose a key challenge with their insulating nature, which prevents charge transport and thus leads to effects such as Joule heating. Therefore, inorganic ion pairs were employed, specifically LiX (X = Cl^–^, Br^–^, I^–^), as alternative passivating agents.^274^ The straightforward addition of LiX to the perovskite precursor solution established a halide-rich environment that effectively passivated halide vacancies formed during the crystallization and prevented the aggregation of free cesium on the surface without affecting the perovskite’s crystallinity and electronic properties. As a result, an EQE of 16.2% was achieved, accompanied by enhanced operational stability. Likewise, the incorporation of KBr has been shown to improve film quality and reduce defect density.^275,276^ Potassium ions can effectively passivate grain boundary defects, thereby suppressing halide migration and reducing the level of trap state formation. For instance, Ren et al. reported a ∼65% decrease in trap state density in KBr-treated perovskites compared to their pristine counterparts.^277^ The KBr passivation led to a notable improvement in EQE, from 0.78% to 1.45%. Moreover, yttrium chloride (YCl_3_) was tested as a passivating agent for blue-emissive polycrystalline perovskites (PEACl: CsPbBr_3_), and it significantly improved the PLQY, from 19.8% to 49.7%, leading to an EQE of up to 11% (Figure 9e,f).^268^ Single-crystal analysis and inductively coupled plasma mass spectrometry (ICP-MS) confirmed the presence of yttrium from the surface to the bulk with a gradual decrease in concentration. The enhancement was attributed to the incorporation of yttrium, leading to the passivation by creating an energy barrier at the grain shell, effectively confining charge carriers and promoting radiative recombination (Figure 9e). The effectiveness of ionic species in tailoring perovskite interfaces highlights the pivotal role of electrostatic interactions in defect mitigation and lattice stabilization. From vacancy passivation to the suppression of ion migration, these strategies offer versatile pathways to enhance device durability and emission efficiency.

#### Lewis Acid–Base Interaction

In MHPs, the undercoordinated B-cations (e.g., Pb^2+^) behave as Lewis acids, where halides (Cl^–^, Br^–^ and I^–^) behave as Lewis bases. Alternatively, these bases can be replaced with various functional groups such as hydroxyl, amine, carbonyl, carboxyl, phosphine oxide, sulfur oxide, arsenic oxide, etc., consisting of the electron pairs. Thus, these can form acid–base adducts with MHPs, thereby passivating the surface defects, leading to improved EQE. Table 8 summarizes different passivating molecules that interact with MHPs through Lewis acid–base interactions.^17^

**Table 8:**
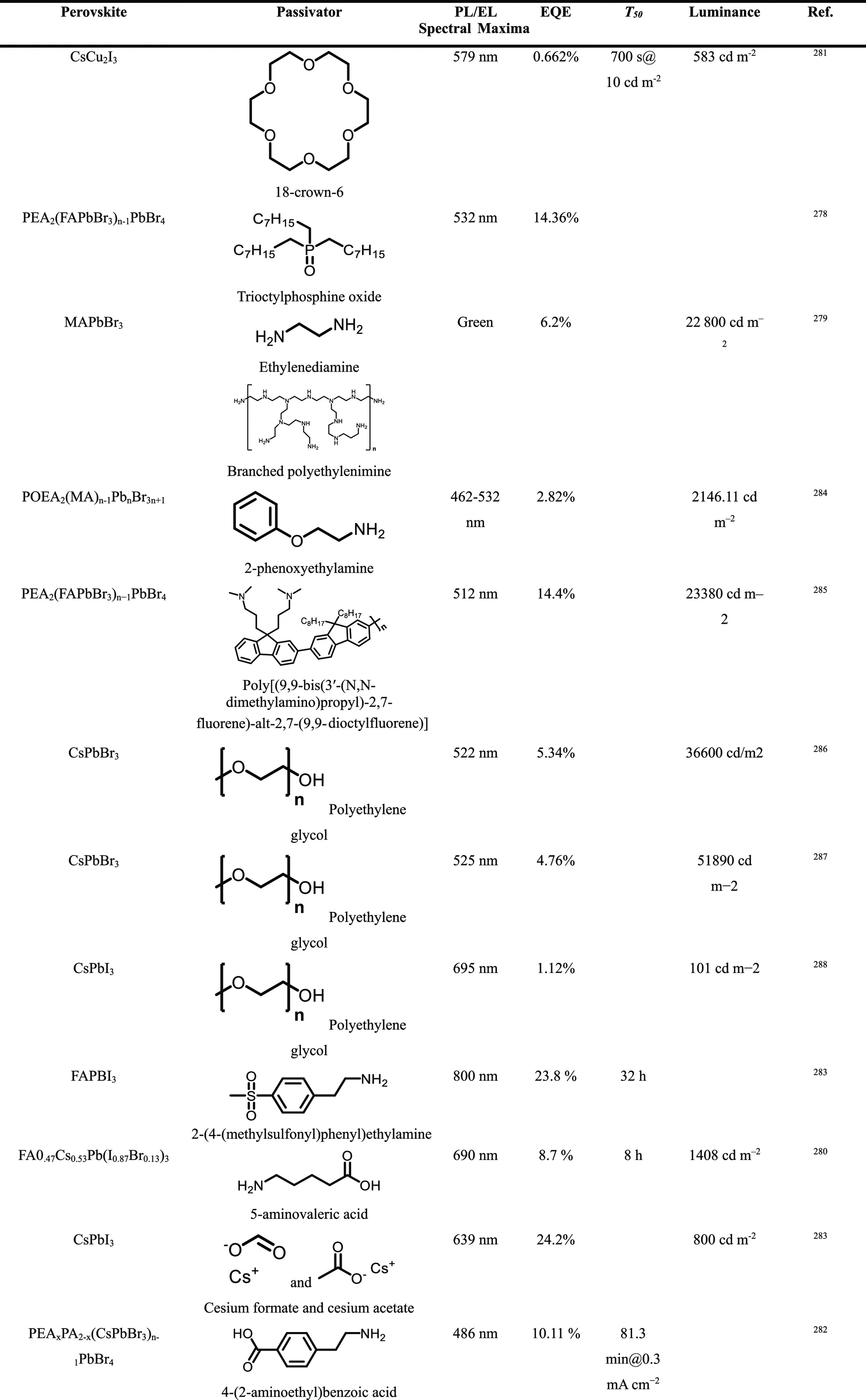
List of Passivating Molecules That Interact with Perovskite via Lewis Acid–Base Interactions^278,279,280,281,282,283,284,285,286,287,288^

For instance, quasi-2D perovskites generally have smaller crystal sizes than their 3D counterparts; they tend to exhibit higher defect densities and more trap states within the film and at grain boundaries. To address this issue, a trioctylphosphine oxide (TOPO) layer was introduced for surface passivation, leading to an increase of PLQY from 57.3% to 73.8%.^278^ The interaction between TOPO and undercoordinated lead ions was confirmed by FTIR spectroscopy, which showed a shift in the P=O stretching vibration from 1150 to 1100 cm^–1^. Notably, TOPO passivation enabled PeLEDs to reach an EQE of 14.36%, the highest reported efficiency at the time. Meanwhile, it has been found that electronic traps in MHPs can lead to emission blinking in perovskite LEDs. This was attributed to the presence of common defects in solution-processed MHPs. To suppress this effect, amine-based passivators, such as polyethylenimine (PEI) and ethylenediamine (EDA), unlike the previously discussed ammonium-based molecules, were employed as passivation layers on top of the MAPbI_3_ perovskite. This treatment led to enhanced photoluminescence (PL) intensity (Figure 10a), increased carrier lifetime, and reduced PL blinking.^279^ The passivation mechanism in this case has been attributed to interactions between nitrogen lone pairs and undercoordinated lead ions in the perovskite. The passivation additionally inhibited ion migration, thereby preventing Ag electrode corrosion, as confirmed by XRD and XPS measurements, which indicated the formation of AgBr.

**Figure 10:**
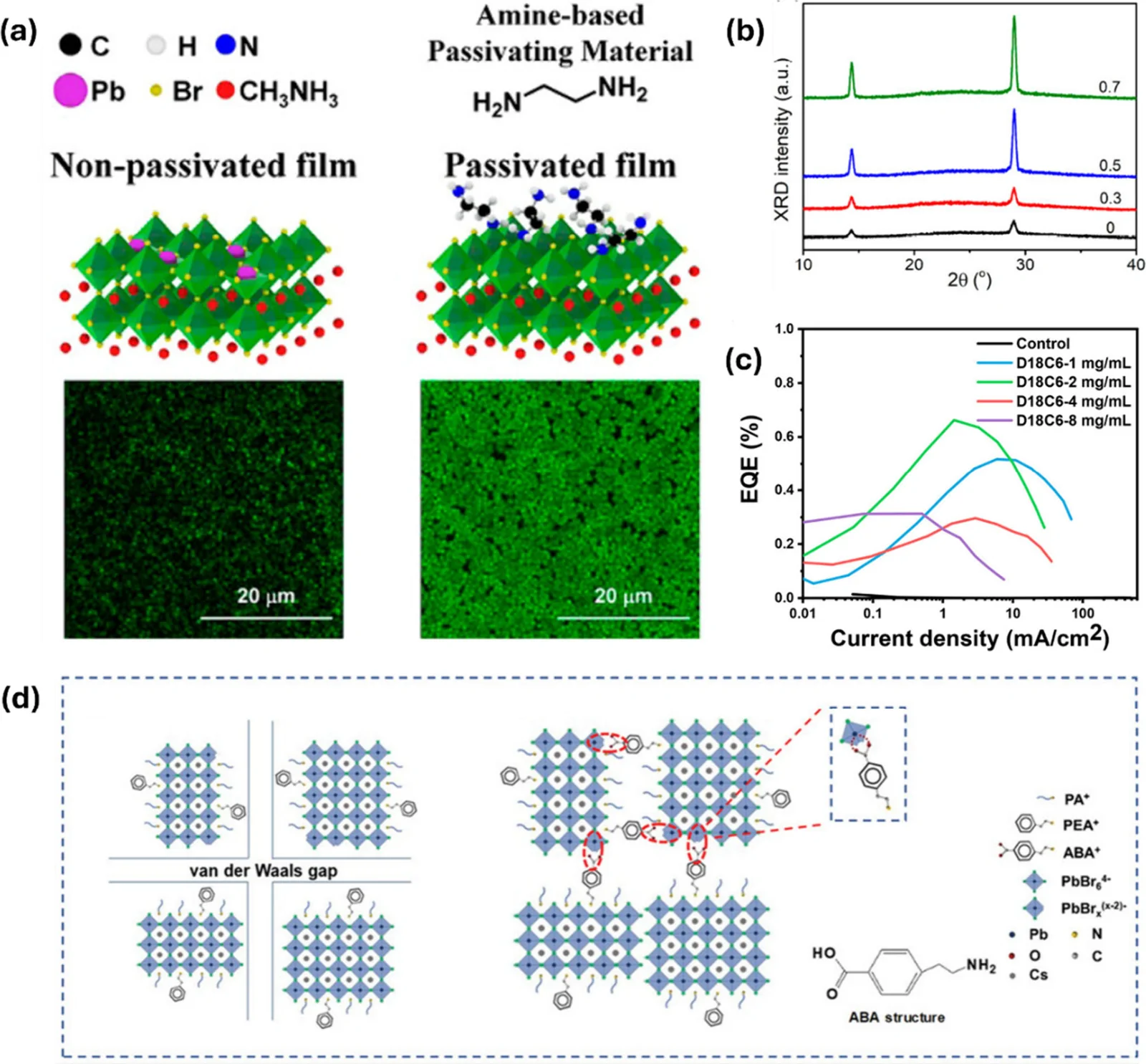
(a) Confocal images of MAPBI_3_ film with and without amine passivation showing enhanced PL intensity. Reproduced with permission from ref 279. Copyright 2017 American Chemical Society. (b) XRD patterns of FA_0.47_Cs_0.53_Pb­(I_0.87_Br_0.13_)_3_ films incorporating varying concentrations of 5-aminovaleric acid (5AVA). Reproduced with permission from ref 280. Copyright 2018 American Chemical Society. (c) EQE–*J* plots for perovskite films with varying D18C6 content and the control sample. Reproduced with permission from ref 281. Copyright 2024 American Chemical Society. (d) Schematic illustrating enhanced interactions within pristine perovskite films upon incorporation of 4-(2-aminoethyl) benzoic acid (ABA). Reproduced with permission from ref 282. Copyright 2020 Wiley-VCH GmbH.

2-(4-(Methylsulfonyl)­phenyl)­ethylamine (MSPE), a molecule containing both a sulfonyl (S=O) group and an amine (−NH_2_) functionality has shown effectiveness in passivating α-phase FAPbI_3_ films through multiple mechanisms.^283^ This passivation led to enhanced crystallinity, as evidenced by XRD and suppressed Joule heating, thereby enabling the device to maintain high EQE even under high-brightness conditions. The PLQE increased significantly, reaching 40% at an excitation intensity as low as 0.02 mW cm^–2^. Additionally, the operational half-life improved markedly from 5 to 32 h, and a notable reduction in energetic disorder was evidenced using hyperspectral microscopy. The corresponding PeLEDs exhibited near-infrared emission at ∼800 nm and achieved an EQE of 20%. Notably, the interaction between the S=O group and Pb^2+^ was analyzed using FTIR spectrscopy and XPS. In FTIR, exposure of MSPE to PbI_2_ resulted in broadening and a red shift of the S=O stretching vibration, indicating bond formation. Complementarily, XPS spectra showed a shift toward higher binding energy for O 1s and a corresponding lower shift for Pb 4f, confirming electron density transfer from oxygen to lead, evidence of strong coordination between MSPE and the perovskite surface.

Alongside neutral passivators, anionic and lone pair-containing functional groups like carboxylates have been explored for coordinating with Pb^2+^ ions, effectively passivating defects and enhancing both PLQY and EQE. For example, the use of 5-aminovaleric acid (5-AVA) passivator led to larger and more uniform grain formation, as evidenced by SEM and XRD analyses. Importantly, the XRD patterns did not display any new peaks upon 5-AVA treatment (Figure 10b).^280^ Instead, a reduction in the full width at half-maximum (fwhm) was observed, suggesting that no layered perovskite phase was formed; rather, the grain size increased with higher 5-AVA content. In addition, PLQY significantly improved from 0.1% to 30% at a low excitation intensity at ∼0.1 mW cm^–2^, indicating effective defect passivation. Consequently, the corresponding PeLEDs exhibited an EQE of 8.7% with an emission at 690 nm and enhanced phase stability. Generally, quasi-2D PeLEDs operate through energy funneling across different n-phases, with emission occurring from the phase with the narrowest bandgap. However, conventional organic spacer cations such as PEA^+^ and BA^+^ create van der Waals gaps between the phases, impeding efficient energy transfer. To address this limitation, a bifunctional ligand, 4-(aminoethyl)­benzoic acid (ABA), was introduced to simultaneously anchor and bridge adjacent MHPs of similar or different n-phases via its amine and carboxylate groups, thereby reducing the interphase distance as illustrated in Figure 10d.^282^ The interaction between the surface’s Pb^2+^ and the carboxylate group was confirmed through FTIR, XPS, and DFT calculations. The incorporation of ABA weakened the van der Waals interactions and strengthened the interphase coupling (Figure 10d). This facilitated more efficient energy funneling, increasing the EQE from 7.07% to 10.11% and extending the operational stability to 81.3 min.

Moving toward lead-free LEDs, copper-based perovskite CsCu_2_I_3_ has received increasing attention. A key challenge, however, is that copper incorporation inevitably causes structural disorder, leading to a high defect density that requires effective passivation.^281^ To address this, researchers have used crown ethers, which can coordinate with surface cations (Cs^+^ and Cu^+^) through the lone pairs on the ether oxygens (C–O–C), depending on the ionic size. The interaction of 18-crown-6 (18C6) and dibenzo-18-crown-6 (D18C6) with copper and cesium was confirmed by FTIR and XPS analysis. Espicially, D18C6 showed a stronger interaction, with larger shifts from the reference peaks in both measurements. This led to more effective passivation, increasing the PLQY from 4.71% to 19.9%, and achieving an EQE of 0.662% for the D18C6-treated CsCu_2_I_3_ perovskites (Figure 10c). Overall, acid–base interactions offer a versatile and effective approach to passivating under-coordinated sites and mitigating defects in perovskite films. By leveraging functional groups capable of coordinating with lead and other cations, these strategies enhance crystallinity, reduce nonradiative recombination, and improve device efficiency and stability.

#### Additives in Precursor Solution: Synergistic Phase Control and Passivation

Despite interesting features of quasi 2D perovskites, (RNH_3_)_2_A_*n*–1_B_*n*_X_3*n*+1_, they suffer from poor phase distribution, which ultimately leads to energetic disorder, and thus the poor LED performance.^289^ When fabricated, the perovskite film contains multiple phases of perovskites with different *n*-values. As discussed above, the EL maxima of this film are governed by the narrowest-bandgap phase, with charge carriers (in operational LED) funneling from higher-bandgap n-phases to this phase, where emission ultimately occurs. Thus, the inhomogeneous distribution of n hinders the paths of charge carrier funneling and inevitably leads to a high density of defects, ultimately degrading the LED performance.^290^ A promising way to address these challenges is to introduce passivator molecules into the precursor solutions, thereby facilitating the formation of phase-pure, defect-free thin films of both 2D and 3D perovskites (Figure 11).^291^ Table 9 summarizes some of the examples where additives are incorporated into MHPs by adding them to corresponding precursor solutions.

**Figure 11:**
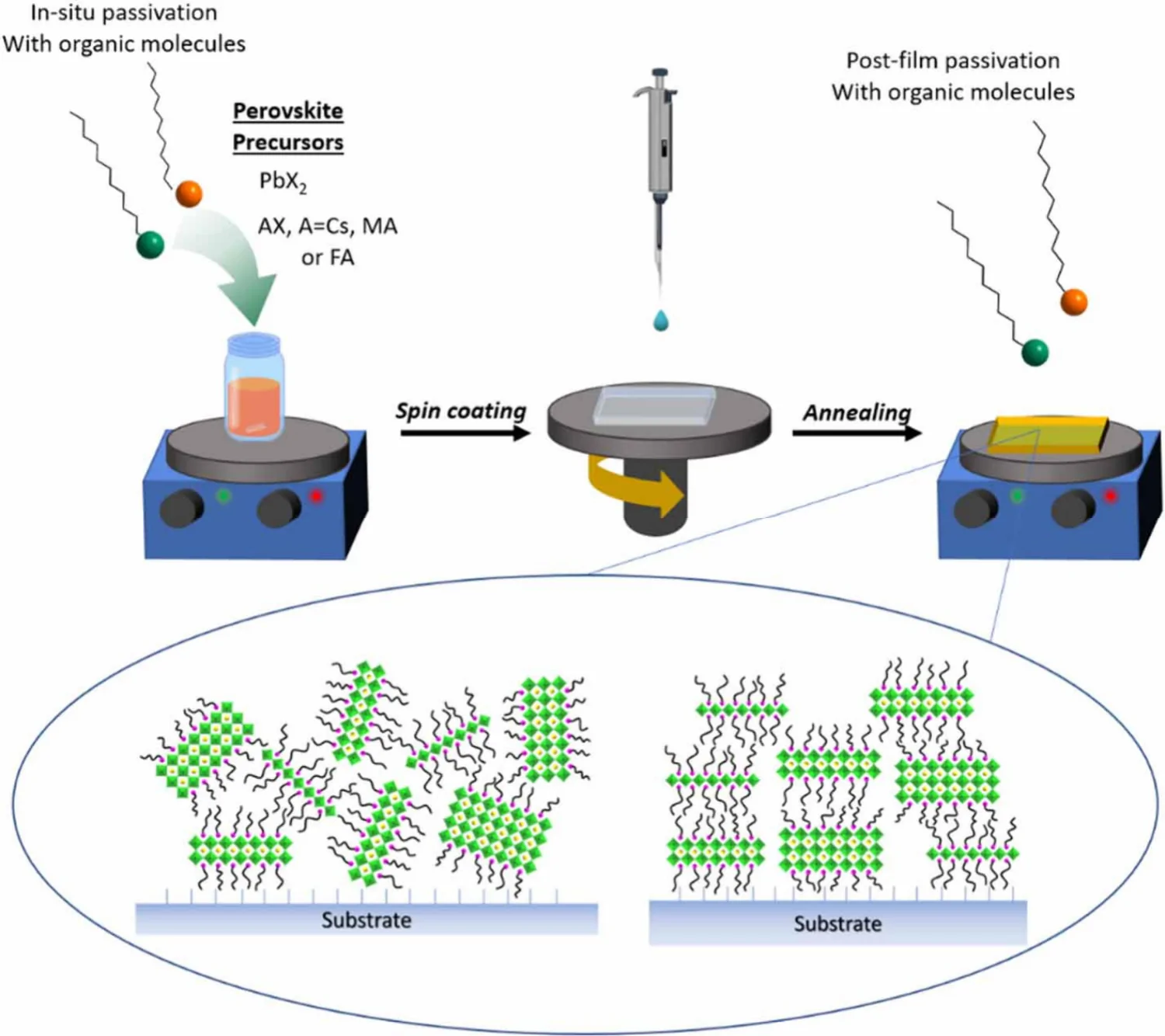
Schematic illustration of MHPs passivation achieved by incorporating passivators either directly into the precursor solution or through the post-treatment of the perovskite film. Reproduced with permission from ref 291. Available under a CC-BY 4.0. Copyright 2024 The Author(s). Published by IOP Publishing Ltd.

**Table 9:**
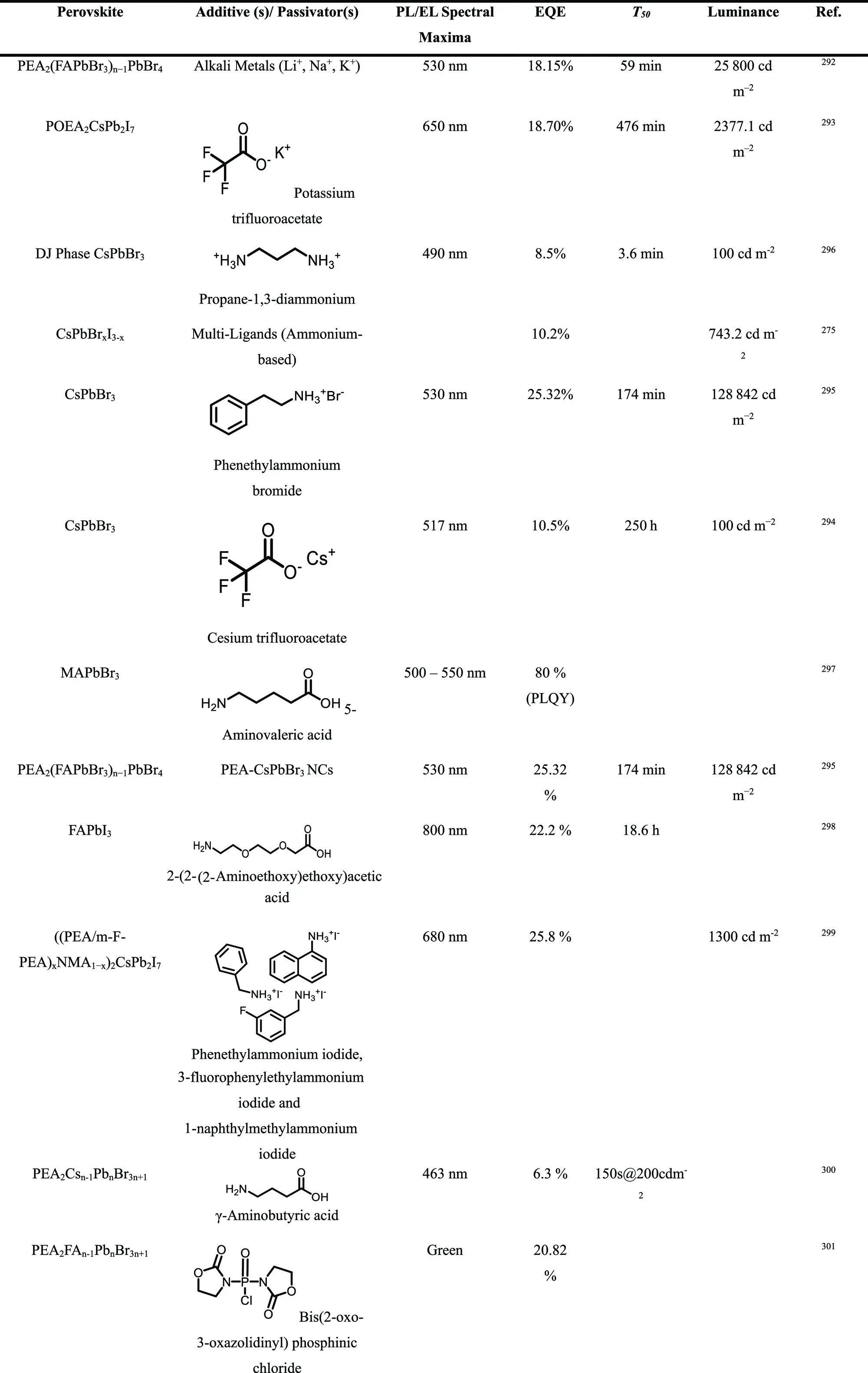
List of Additives That Regulate the Growth of Quasi-2D Perovskite Films and Provide Defect Passivation^275,292,293,294,295,296,297,298,299,300,301^

For example, KBr was used as an additive to regulate the growth of high-n phases in the quasi-2D perovskite PEA_2_(FAPbBr_3_)_*n*−1_PbBr_4_.^292^ This additive promotes the homogeneous distribution of low-n phases by interacting with PbBr_6_^–^ octahedra and slowing down the growth, facilitating efficient energy funneling, and achieving a PLQY of up to 66.8% (Figure 12a). The SEM images, along with AFM analysis, indicated a reduction in the surface roughness of the perovskite film. Further investigation using transient absorption spectroscopy revealed faster and more efficient energy transfer, leading to an EQE as high as 18.15% in the KBr-treated film. Similarly, potassium trifluoroacetate (KTFA) has also shown promise for regulating the phase distribution and defect passivation of red-emitting quasi-2D-perovskite (POEA_2_CsPb_2_I_7_)-based LEDs.^293^ Figure 12b depicts the various proposed interactions between KTFA and the perovskite structure. Potassium ions engage in ionic interactions with iodide ions, effectively suppressing their migration and thereby mitigating vacancy formation. Simultaneously, the carboxylate group binds to undercoordinated lead atoms, contributing to surface passivation. Additionally, the fluorine atoms in KTFA form hydrogen bonds with the hydrogen atoms of the ammonium group in POEA. These interactions, which are supported by XPS, NMR, and FTIR analyses, regulate the phase distribution by suppressing the formation of low-*n* phases (*n* = 2), thereby promoting efficient energy transfer to the emissive phase. This results in a substantial improvement in EQE, from 11.86% in the control sample to 18.70% with KTFA incorporation (Figure 12b).

**Figure 12:**
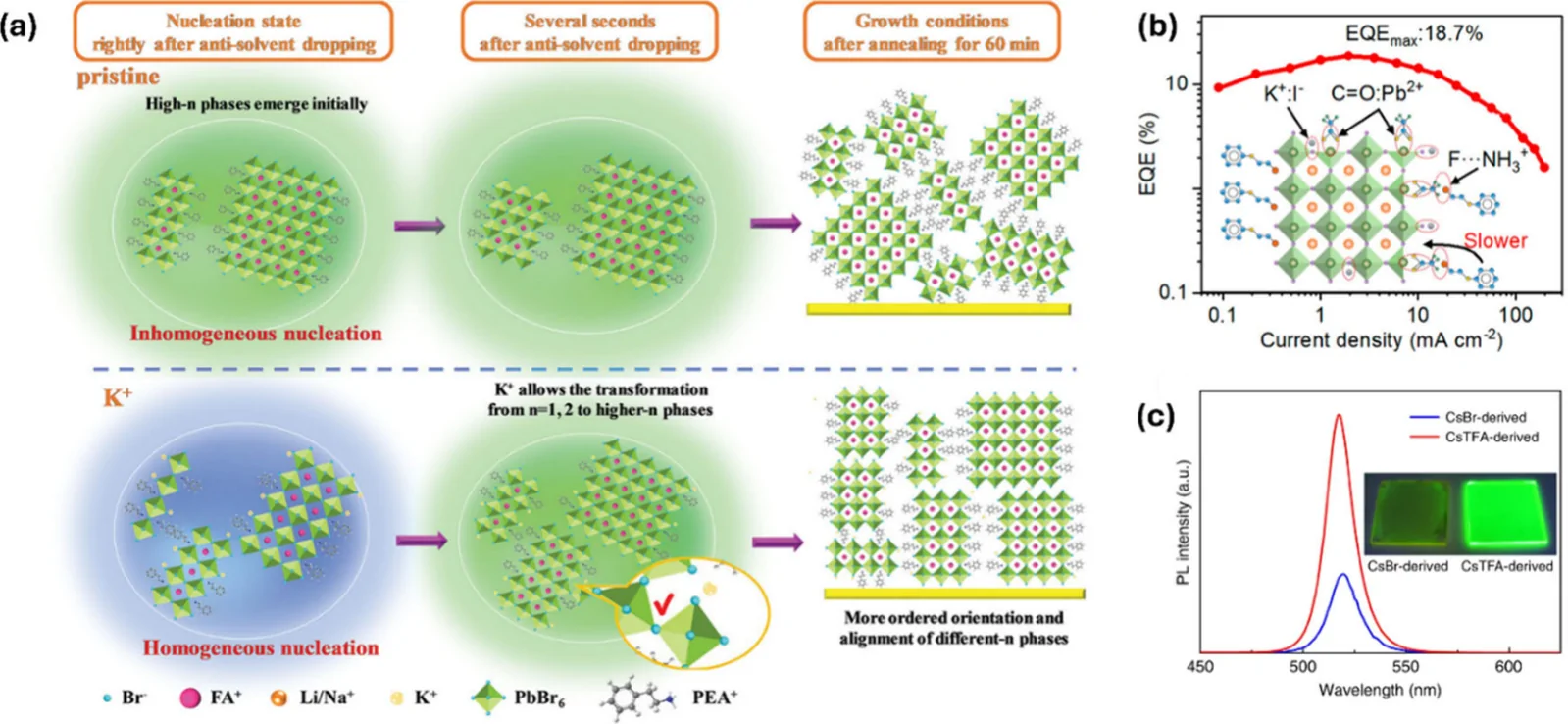
(a) Mechanistic illustration of the growth kinetics of PEA_2_(FAPbBr_3_)_*n*−1_PbBr_4_ in the presence and absence of K^+^. Reproduced with permission from ref 292. Copyright 2021 Wiley-VCH GmbH (b) The various possible interactions between the passivator, KTFA, and the perovskite, along with the EQE versus current density curve illustrating the LED performance after passivation. Reproduced with permission from ref 293. Copyright 2023 American Chemical Society. (c) PL spectra and UV-illuminated films (inset) of CsPbBr_3_ perovskite derived from CsBr and CsTFA precursors. Reproduced with permission from ref 294. Available under a CC-BY 4.0. Copyright 2019 The Author(s). Published by Springer Nature.

In another work, instead of using organic additives, phenethylammonium-modified CsPbBr_3_ nanocrystals were incorporated into quasi-2D perovskites with the formula PEA_2_(FAPbBr_3_)_*n*−1_.^295^ These nanocrystals act as heteronuclear seeds, effectively reducing surface roughness and mitigating halide vacancies, which in turn suppresses nonradiative recombination. Importantly, they also introduce an additional energy transfer pathway, facilitating the transfer of energy from low-n phases to the nanocrystals and, subsequently, to the emissive phases. Quantitatively, this strategy enhanced energy transfer efficiency by a factor of 3 and reduced the nonradiative recombination rate by 70%. Overall, the optimized device achieved an EQE of 25.32%, representing a 1.5-fold enhancement along with pure green emission centered at 530 nm.

Typically, all-inorganic perovskite films exhibit larger grain boundaries and thus the defects, which remains key obstacle in achieving efficient LEDs despite their superior stability over organic–inorganic hybrid perovskites.^294^ However, in a report,the simple replacement of a typical CsBr precursor with cesium trifluoroacetate (CsTFA) in the precursor solution resulted in the formation of smooth, pinhole-free, fine-grained, and highly emissive perovskite films as presented in Figure 12c. Remarkably, the TFA-based LED demonstrated a 17-fold improvement in operational stability, achieving a half-life of 250 h and an EQE of 10.5%, significantly outperforming its CsBr-based counterpart. These examples highlight that the in situ passivation of MHPs by adding additives in precursor solution is a promising approach for making phase pure, defect-free MHPs with smooth surfaces for the fabrication of efficient LEDs.

### Nanocrystal-Based PeLED

Nanocrystal PeLEDs offer additional advantages over bulk thin films due to their higher PLQY, the flexibility to modify surface chemistry, and the enhanced stability provided by surface ligands. However, as mentioned earlier, the ligand binding to perovskite NC’s surface is highly dynamic, and they often get detached during the washing process, leading to a high density of surface defects. Surface passivating agents in PeLEDs are systematically classified based on their molecular architecture and their specific interaction mechanisms with surface defects. These agents play a pivotal role in neutralizing trap states, enhancing crystallinity and film morphology, promoting charge carrier balance, and ultimately boosting radiative recombination to elevate the EQE.

While numerous comprehensive reviews over the past decade have focused on discussing the strategies reducing the defect density in colloidal perovskite nanocrystals, our discussion will specifically center on how these advancements translate into enhanced efficiency in PeLEDs.^252,302–308^ Figure 13 illustrates a comprehensive overview of various chemical passivants recently identified as promising candidates for effective surface engineering of lead–metal halide perovskite nanocrystals (LHP/MHP NCs).

**Figure 13:**
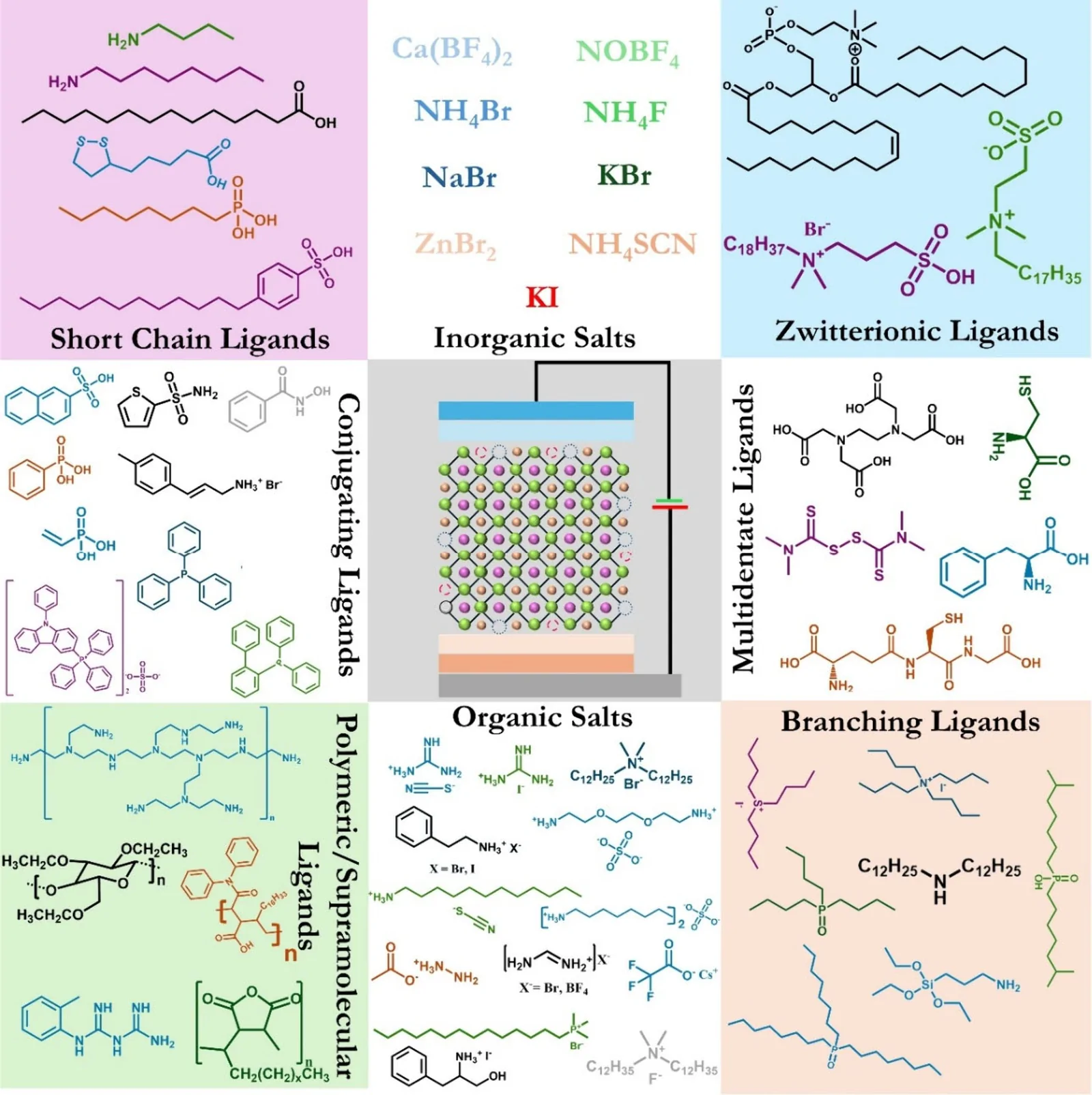
This Schematic Classifies the Chemical Reagents, i.e., Short Chain Ligands, Inorganic Salts, Zwitterionic Ligands, Conjugating Ligands, Multidentate Ligands, Polymeric/Supramolecular Ligands, Organic Salts, And Branching Ligands (Depending on Their Chemical Structure), Used for Surface Passivation of MHP NCs to Enhance PeLED Performance.

#### Organic Salt Passivation

Various organic salts have been tested for surface passivation of MHP nanocrystals, leveraging their functional cationic and anionic groups to donate halides or coordinate with under-coordinated Pb^2+^ ions, thereby effectively healing halide vacancies and suppressing trap states. Despite their defect-tolerant nature, Br- and Cl-based LHP NCs suffer from reduced PLQY after the washing process. On the other hand, Cl-based LHP NCs are less defect-tolerant and thus they exhibit weak photoluminescence (usually 1–5% PLQY) right from their colloidal synthesis.^28,252^ To passivate deep Cl-traps, Ma et al. introduced hydrazinium acetate in mixed halide CsPbBr_*x*_Cl_3–*x*_, where hydrazinium (Hz^2+^) captures isolated Cl-atoms to form a stable Hz–Cl–Cs bridge (Figure 14a).^309^ This reinforced surface passivation doubled the EQE from 3.78% to 7.82%. Similarly, a nonpolar solvent-soluble salt, *n*-dodecylammonium thiocyanate (DAT) treatment in CsPb­(Br_*x*_Cl_1–*x*_)_3_, achieved a notable EQE of 6.3% at 471 nm by passivating Cl-related defects.^310^ More recently, it has been demonstrated that the use of cesium trifluoroacetate for CsPbCl_3–*x*_Br_*x*_ QDs, where trifluoroacetate anions coordinate with under-coordinated Pb^2+^ sites, leads to a record EQE of 23.5% at 490 nm.^311^ Similarly, 2,2’-(ethylenedioxy)-bis­(ethylammonium) sulfate form strong bonds with undercoordinated Pb atoms, preventing CsPbBr_3_ NPL’s coalescence to achieve color pure EL under external bias.^312^

**Figure 14:**
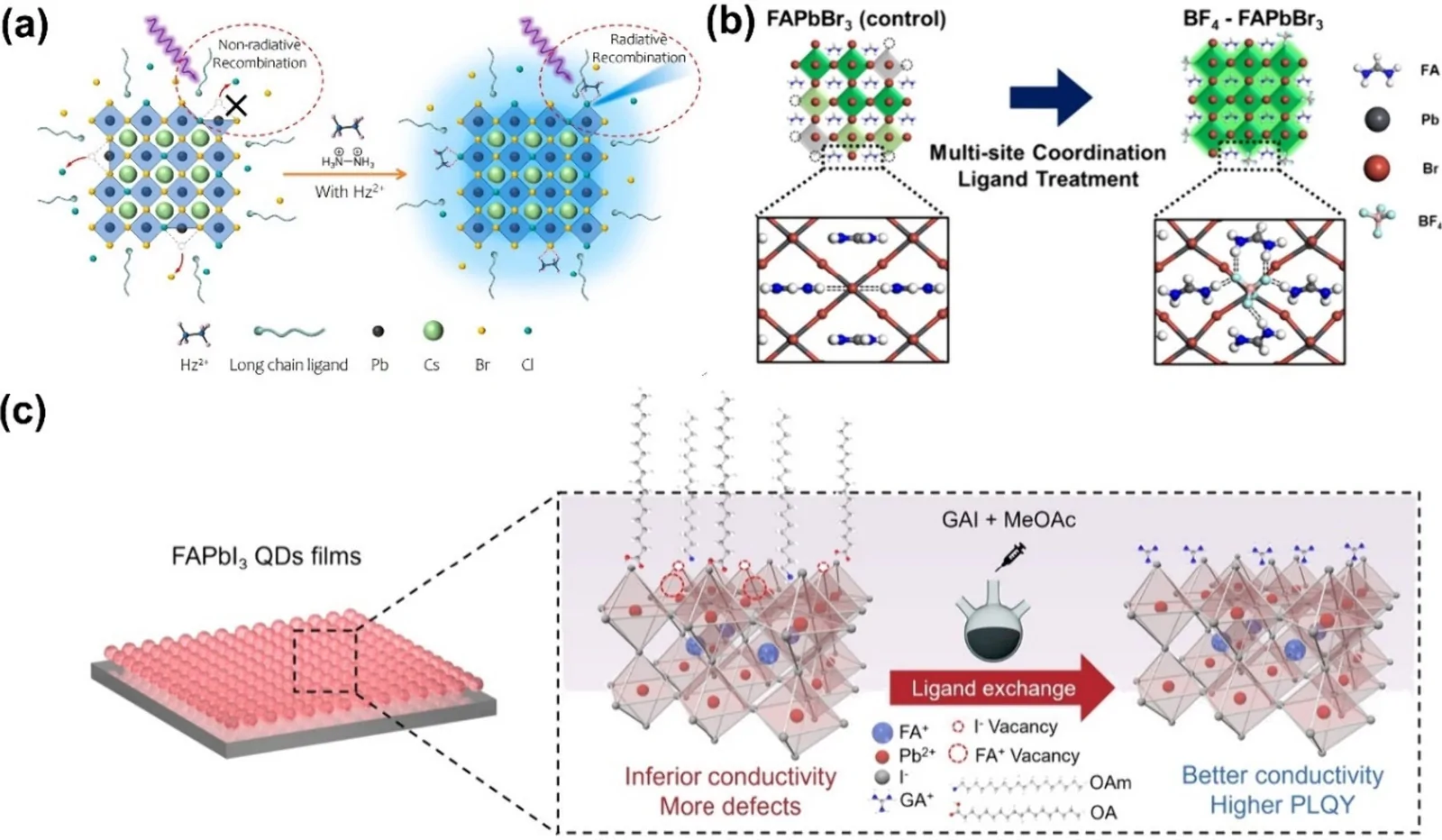
(a) Illustration of the Cl vacancies in the pristine NCs (left) and defect suppression after the passivation of Hz^2+^ (right). Reproduced with permission from ref 309. Copyright 2024 American Chemical Society. (b) Illustrations of control and BF_4_^–^ ion passivated FAPbBr_3_ PNCs and multisite coordination of BF_4_^–^ ion on the PNCs surface through hydrogen bonds with FA^+^ cations. Note that hydrogen bonds are shown as black dashed lines. Reproduced with permission from ref 313. Copyright 2025 American Chemical Society. (c) Schematic illustration of the in situ ligand exchange process of FAPbI_3_ QDs by guanidinium iodide (GAI) dissolved in antisolvent methyl acetate (MeOAC). Reproduced with permission from ref 314. Copyright 2024 Wiley-VCH GmbH.

In another related work, Lee et al. employed a multisite coordination approach for passivation of FAPbBr_3_ NCs using tetrafluoroborate (BF_4_^–^) (Figure 14b).^313^ Their model suggests that one fluorine atom forms a Pb–F bond while another engages in hydrogen bonding with the FA^+^ cation. This dual interaction suppresses Br^–^ migration by increasing the activation energy barrier, significantly improving device stability, and yielded a record EQE of 25.2%. In parallel, passivators like quaternary alkylphosphonium bromide, trimethyl tetradecyl phosphonium bromide were tested and compared them with the widely used trimethyl tetradecyl ammonium bromide.^315^ DFT calculations revealed that the phosphonium ion preferentially occupies Cs^+^ vacancies, with a P–CH_3_ bond oriented perpendicularly to the NC surface, enhancing electrostatic interactions. Further, the lower insulating character of phosphonium salts compared to ammonium analogues enabled a superior EQE of 17.2%, outperforming the 15% EQE achieved with the ammonium counterpart. To combat thermally induced photoluminescence quenching in PeLEDs, didodecyl dimethylammonium fluoride (DDAF) passivation was applied to CsPbBr_3_ nanocrystals and the corresponding LED maintained 80% of their peak EQE even at elevated temperatures of 343 K, demonstrating enhanced thermal robustness.^316^

On the other hand, A-site and iodide vacancies can be passivated with guanidinium-based derivatives such as guanidinium iodide (GAI) and guanidinium thiocyanate (GASCN), as have been widely investigated.^314,317–320^ The small guanidinium ion interacts strongly with the perovskite surface through hydrogen bonding with lattice iodide atoms. This interaction displaces weakly bound surface ligands such as oleic acid (OA) and oleylamine (OAm), resulting in enhanced charge conductivity, improved charge carrier injection, and suppression of Ostwald ripening during device operation. A particularly effective approach involves dissolving GAI in a methyl acetate antisolvent, enabling the introduction of guanidinium ions with strong surface anchoring capability during the NC purification^314^ or post-treatment process (Figure 14c).^319^

To passivate halide vacancy, benzenesulfonate is a promising passivator as it strongly interact with Pb^2+^ as a halide equivalent ion, and applying this to mixed halide CsPb­(Br,I)_3_ NC LEDs resulted in a record EQE of 23.5% at EL peak of 640 nm.^321^ Table 10 presents a curated summary of organic salt-assisted surface passivation strategies reported in the literature, highlighting their corresponding device performance metrics for MHP NC-based PeLEDs.

**Table 10:**
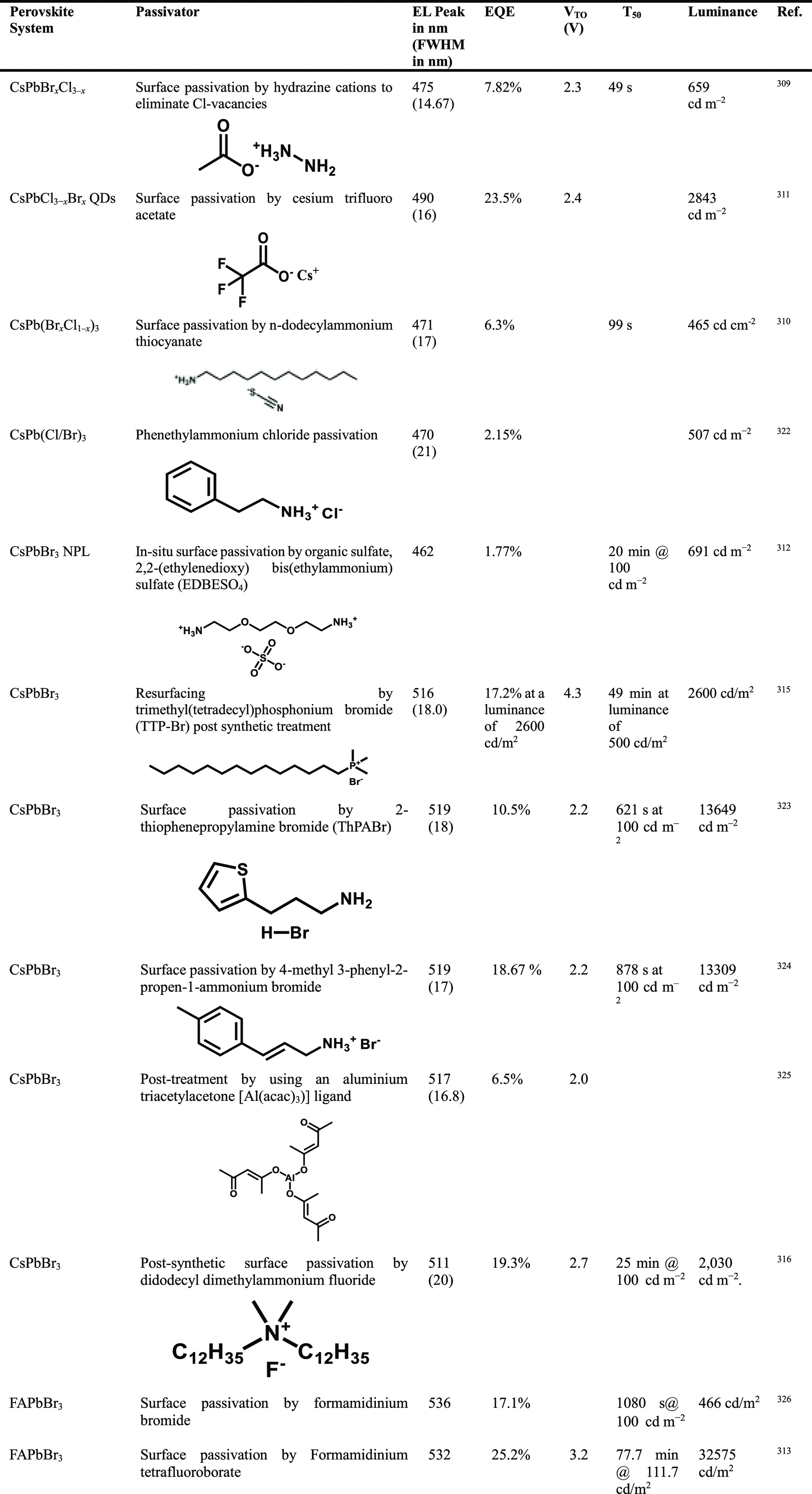

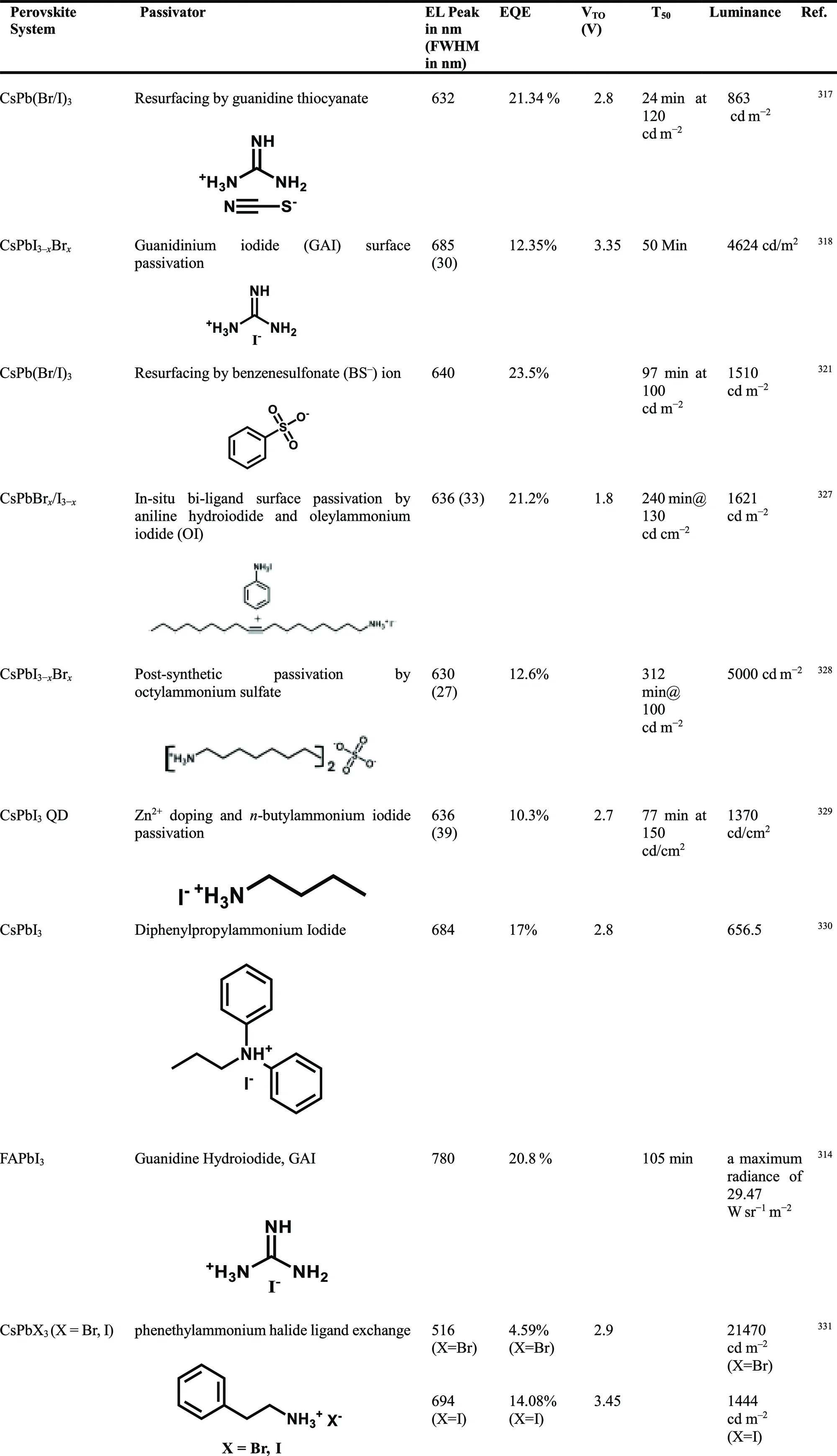

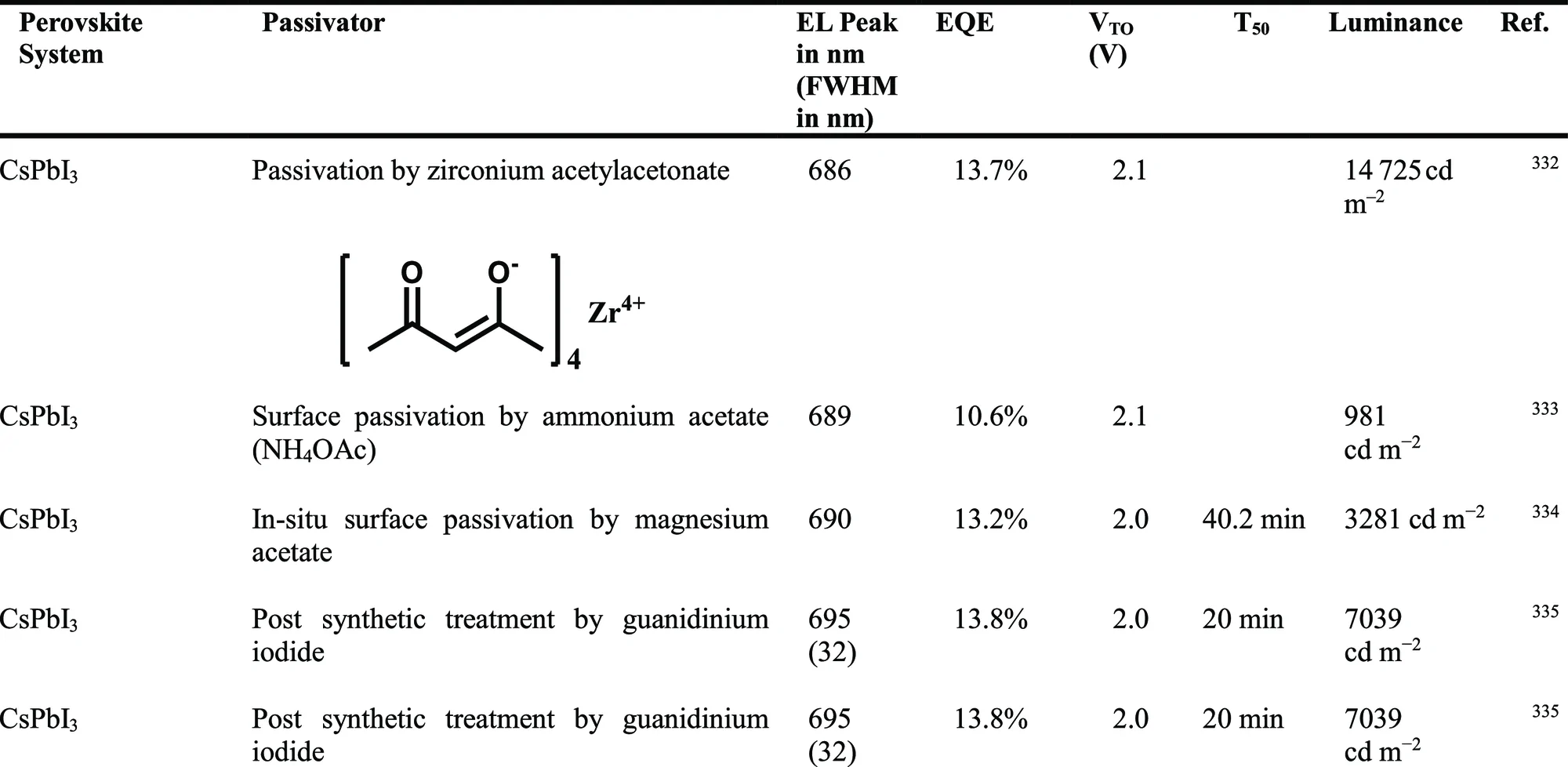
Summary of Organic Salt-Assisted Surface Passivation Strategies Reported for MHP NC-Based PeLEDs, Highlighting Their Influence on Device Efficiency and Performance Parameters^309,310,311,312,313,314,315,316,317,318,321,322,323,324,325,326,327,328,329,330,331,332,333,334,335^

#### Inorganic Salt Passivation

Inorganic salt passivation leveraging both cationic and anionic components has proven highly effective in mitigating surface defects, suppressing ion migration, and promoting well-ordered NC packing for enhanced charge transport in MHP NCs. These improvements collectively boost both device efficiency and operational stability of the corresponding LEDs. In this regard, ZnBr_2_ passivation has been proven to be effective for CsPbBr_3_ NCs, achieving LEDs with an EQE of 16.45% and a maximum luminance of 71850 cd m^–2^.^336^ A recent study demonstrated that NOBF_4_ treatment effectively suppress shallow surface defects like Pb^0^, Pb–O bonds and Pb^3+^ to increase thermal stability of CsPbBr_3_ PeLED.^337^ In another study, Sargent and co-workers have demonstrared a record EQE of 24% with *T*_50_ value of 20 h by replacing the typical organic ligands with purely inorganic atomic ligand, KI, for mixed halide perovskite NC during washing (Figure 15).^338^ In addition, applying this strategy for pure CsPbI_3_ NC resulted in stronger inter QDs interactions, enabling to obtain more oriented and mechanically coupled QDs in solid state, achieving phase-stable LED with an EQE of 23%.^339^ In Table 11, we have highlighted different inorganic salt-assisted surface passivation strategies for MHP NC-based LEDs.

**Figure 15:**
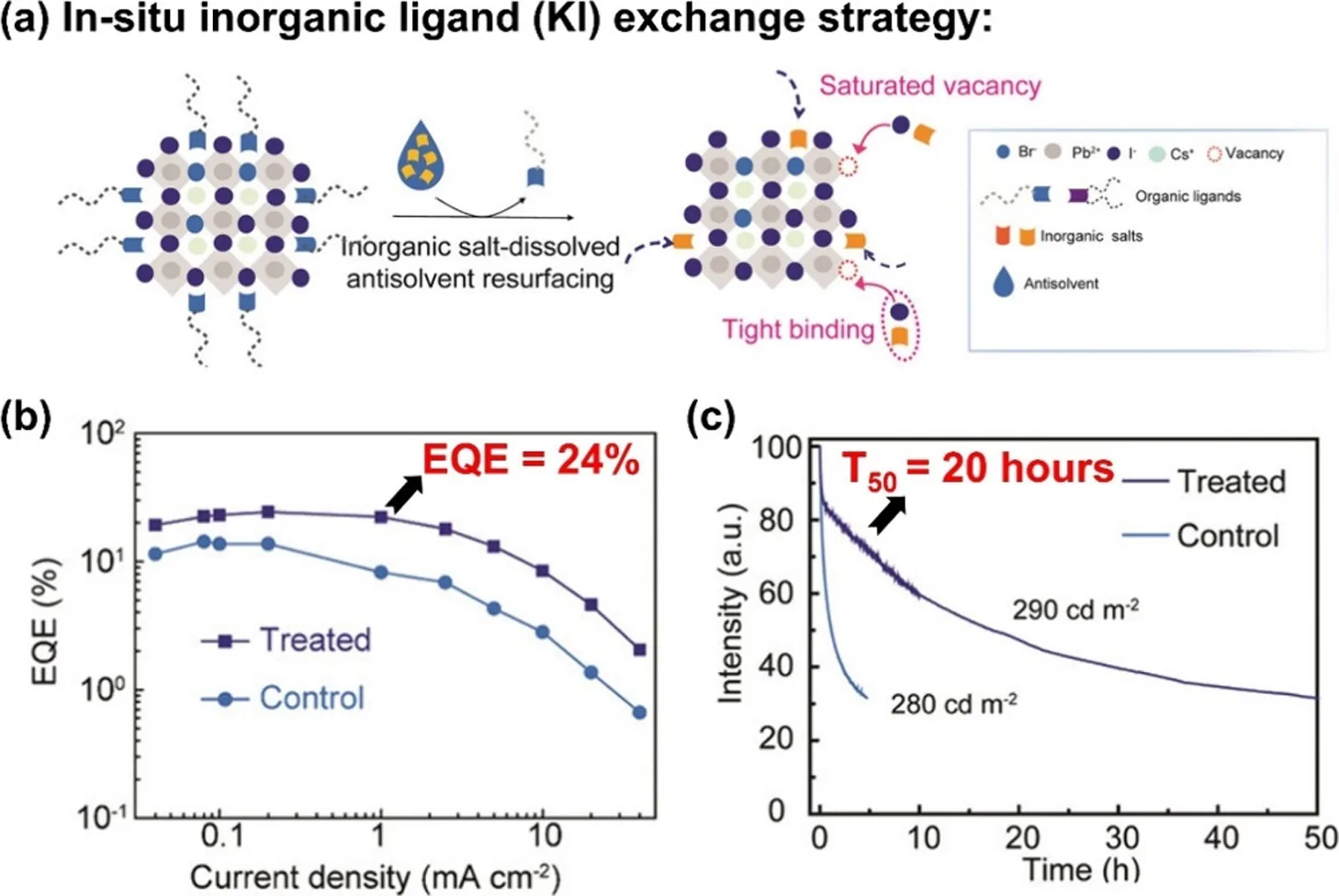
Process and advantage of the in situ inorganic ligand strategy via a mildly polar antisolvent. (b) EQE versus current density curve of treated LEDs. (c) Operating stability at luminances of 290 and 280 cd m^–2^ for treated and control LEDs, respectively. Panels (a)–(c) were reproduced with permission from ref 338. Copyright 2022 Wiley-VCH GmbH.

**Table 11:** Summary of Inorganic Salt-Assisted Surface Passivation Strategies Reported for MHP NC-Based PeLEDs, Highlighting Their Influence on Device Efficiency and Performance Parameters

perovskite system	passivator	EL peak In nm (fwhm in nm)	EQE (%)	VTO (V)	*T*_50_	luminance	ref
CsPb(Br/Cl)_3_	potassium passivation by potassium carbonate in octanoic acid	477 (19)	1.96		4.5 min	86.95 cd m^–2^	^340^
CsPb(Br/Cl)_3_	postsynthetic surface passivation by ammonium fluoride (NH_4_F)	468	1.21		104 s	135 cd m^–2^	^341^
CsPbBr_3_	NaBr and oleic acid	518 (20)	7.6			160900 cd m^–2^	^342^
CsPbBr_3_ QD	surface passivation by ZnBr_2_	518 (18)	16.48	2.4	136 min	71850 cd m^–2^	^336^
CsPbBr_3_	postsynthetic surface passivation by nitrosyl tetrafluoroborate (NOBF_4_)	515	11.24			5684 cd m^–2^	^337^
CsPb(I_*x*_Br_1–*x*_)_3_	antisolvent (ethyl acetate) assisted inorganic ligand (KI) passivation	650	24.4	2.6	20 h@290 cd m^–2^	815 cd m^–2^	^338^
CsPbI_3–*x*_Br_*x*_	KBr surface passivation	637 (31)	3.55	3.6	50 min	2671 cd m^–2^	^343^
CsPbBr_*x*_I_3–*x*_	Zn^2+^ doping with Te^2–^ passivation	633	16.1	2.5		1397.2 cd m^–2^	^344^
CsPbI_3_	inorganic atomic ligand exchange by KI	640 (31)	23	2.0	10 h	200 cd m^–2^	^339^
CsPbI_3_	surface passivation by ammonium thiocyanate	690	10.3	2.2		823 cd m^–2^	^345^

#### Multidentate Ligand Passivation

Multidentate ligands, featuring multiple coordination sites through heteroatoms such as oxygen, nitrogen, and sulfur with lone electron pairs, offer a robust strategy for passivating under-coordinated Pb^2+^ on perovskite NC surfaces via strong covalent bonding. To address surface trap states and suppress ion migration in 2D CsPbBr_3_ nanoplatelets, researchers employed diethylenetriaminepentaacetic acid (DTPA), which provides eight coordination sites for efficient surface coverage.^346^ Similarly, a combination of ethylenediaminetetraacetic acid (EDTA) and reduced l-glutathione were used to passivate MAPb­(I_*x*_Br_1–*x*_)_3_ NCs during their purification. These multidentate ligands demonstrated strong binding affinity to exposed NC surfaces and effectively prevented halide segregation during device operation, enhancing both stability and emission uniformity (Figure 16a,b).^347^

**Figure 16:**
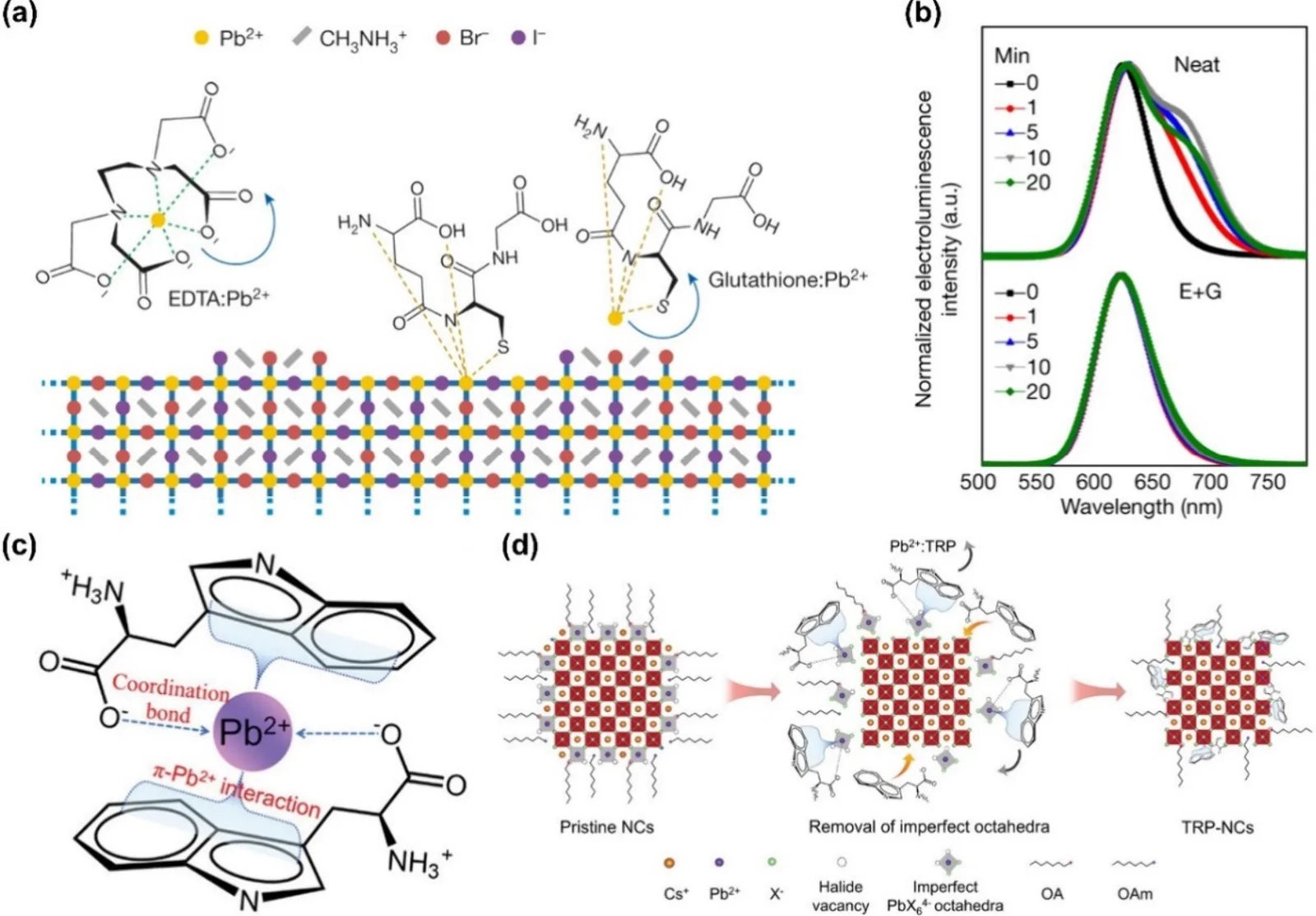
(a) The proposed molecular interactions of glutathione and EDTA with Pb^2+^ atoms on the nanocrystal surface (b) The comparison of stability in electroluminescence spectra for parent (neat) and treated (E+G, EDTA and Glutathione) at different time intervals of LEDs held at a constant current density of 1.5 mA cm^–2^. Panels (a) and (b) were reproduced with permission from ref 347. Copyright 2021 The Author(s). Published by Springer Nature. (c) The molecular interactions between the tryptophan and Pb^2+^ atoms. (d) The illustrated mechanisms of the tryptophan postsynthetic treatment. Panels (c) and (d) were reproduced with permission from ref 348. Copyright 2023 Wiley-VCH GmbH.

Further to increase the transportation of charge carrier with balanced charge injection, different short chain amino acids like tryptophan,^348^ cysteine,^349^ and l-phenyl alanine^350^ have been utilized. The strong cation−π interactions between the PbI_6_ octahedra and the electron-rich indole ring of tryptophan effectively passivate surface defects and enhance ligand binding (Figures 16c,d), resulting in LEDs with record EQE of 22.8%, and a maximum luminance of 12910 cd m^–2^ of CsPb­(Br/I)_3_ PeLED device.^348^ Table 12 showcases representative examples of multidentate ligand-based surface passivation strategies.

**Table 12:**
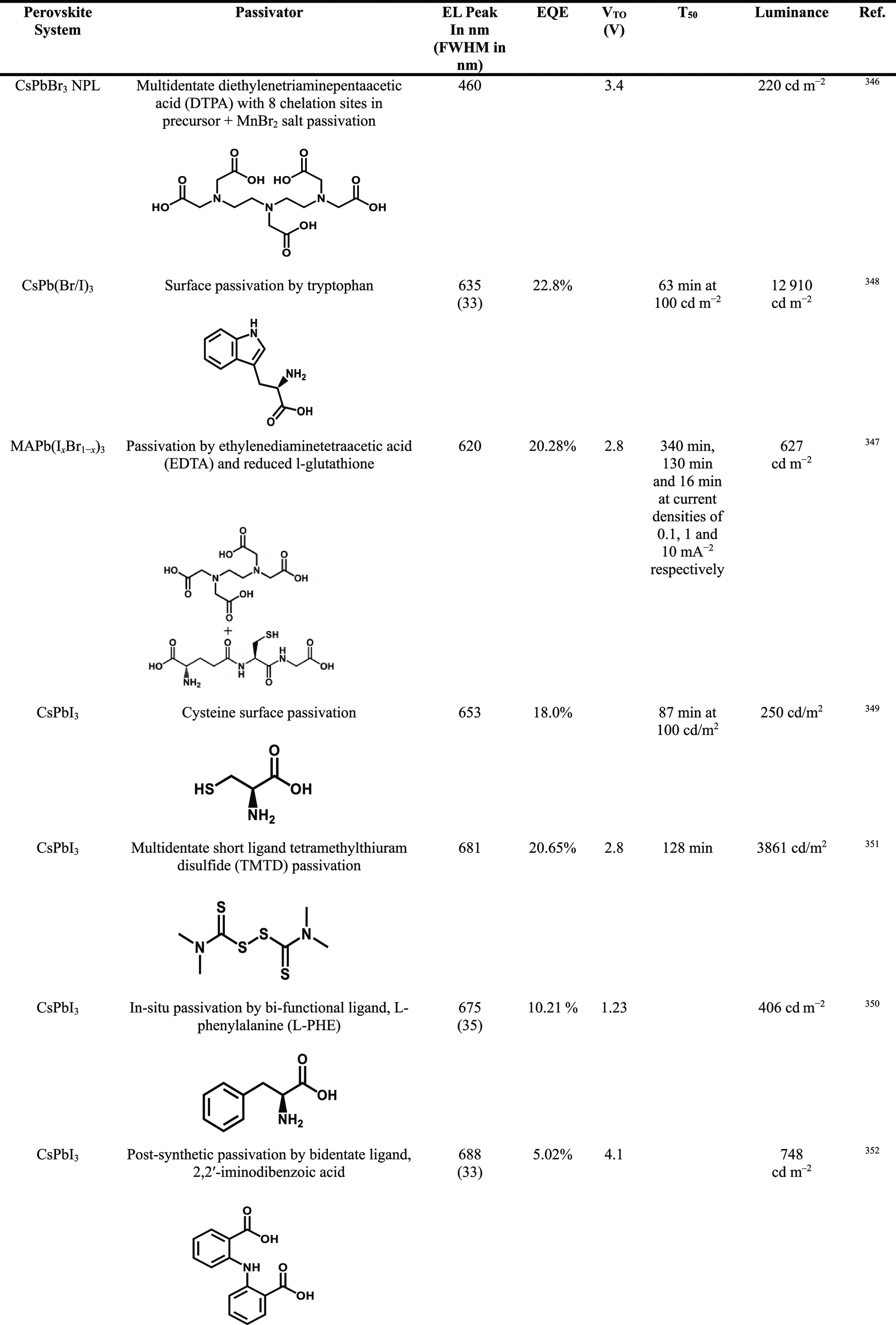
Summary of Multidentate Ligand-Assisted Surface Passivation Strategies Reported for MHP NC-Based PeLEDs, Highlighting Their Influence on the Device Efficiency and Performance Parameters^346,347,348,349,350,351,352^

#### Polymeric/Supramolecular Passivation

Polymeric ligands have emerged as powerful tools for stabilizing perovskite NCs by offering multiple functional groups for surface passivation and structural control, and they can also protect the NCs from external environments, such as air and moisture. For example, polyethylenimine (PEI), rich in −NH_2_ groups, can prevent the coalescence of CsPbBr_3_ NPLs, thereby enhancing their stability and preserving strong blue PL.^353^ To suppress halide segregation and improve operational stability, multifunctional, side-chain-engineered polymer ligands have been found to be efficient.^354^ These polymers feature alternating long alkyl and short aromatic side chains to optimize dispersibility and charge injection. Meanwhile, amide side chains promote ordered NC assembly and carboxylic acid groups on the main chain effectively passivate surface defects, leading to stable electroluminescence (Figure 17a). Similary, the use of poly­(maleic anhydride-*alt*-1-octadecene) (PMA) for stabilizing β-CsPbI_3_ NCs resulted in LEDs with an EQE of 17.8% and remarkable *T*_50_ lifetime of 317 h. PMA interacts with PbI_2_ precursors to guide NC growth and subsequently binds to the NC surface via strong Pb–O interactions.^355^ The notable polymeric surface passivation approaches are summarized in Table 13.

**Figure 17:**
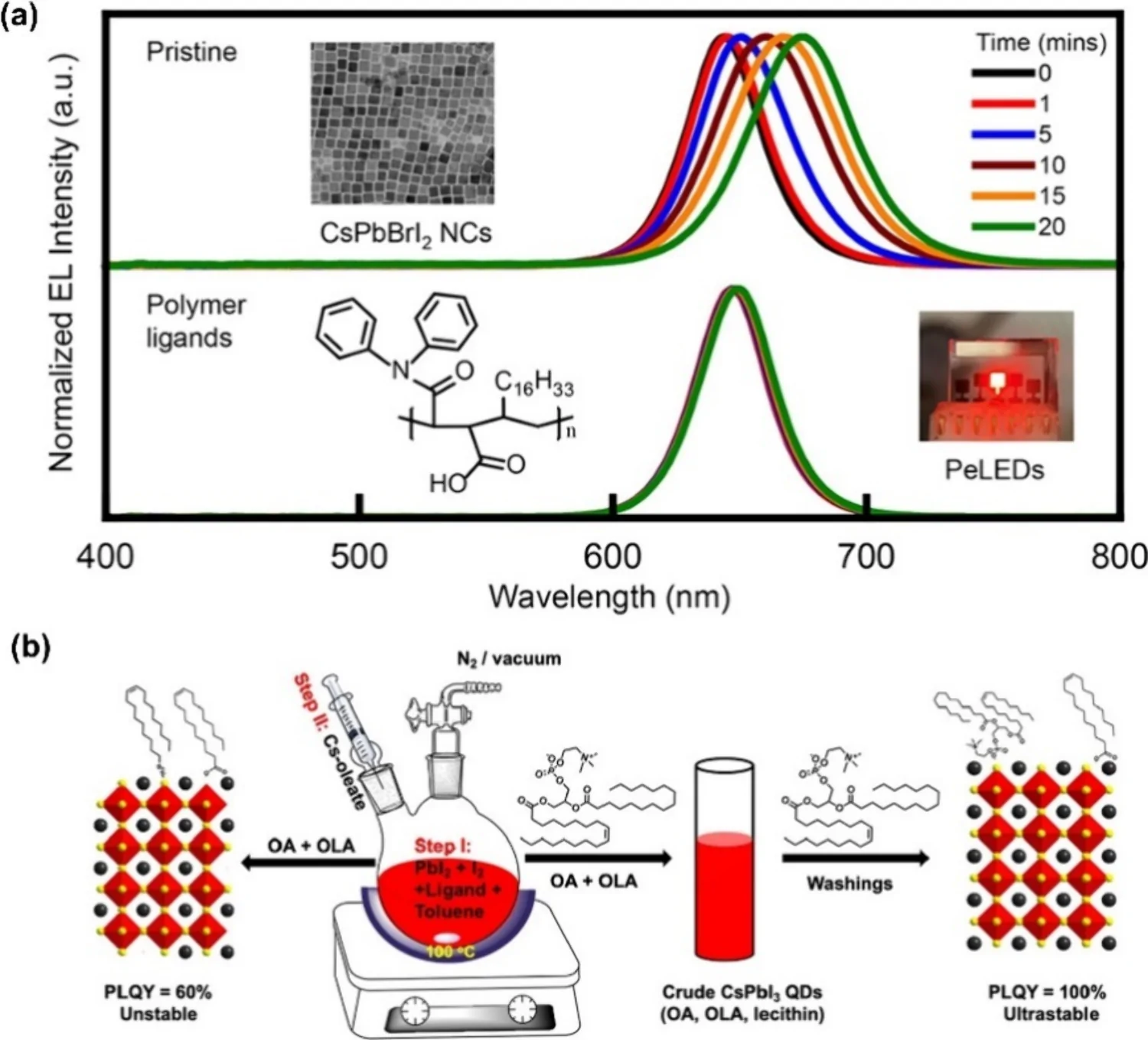
(a) EL spectra of pristine (top) and multifunctional side-chain-engineered polymer-passivated (bottom) CsPbBrI_2_ NC. In the bottom panel, the inset photographic image represents polymer passivated PeLED. Reproduced with permission from ref 354. Copyright 2024 American Chemical Society. (b) Synthesis of CsPbI_3_ QDs in the presence and absence of lecithin as a stabilizing ligand. The QDs prepared with lecithin are washed in the presence of additional lecithin to remove the bound oleylammonium ligands. Reproduced with permission from ref 356. Copyright 2022 American Chemical Society.

**Table 13:**
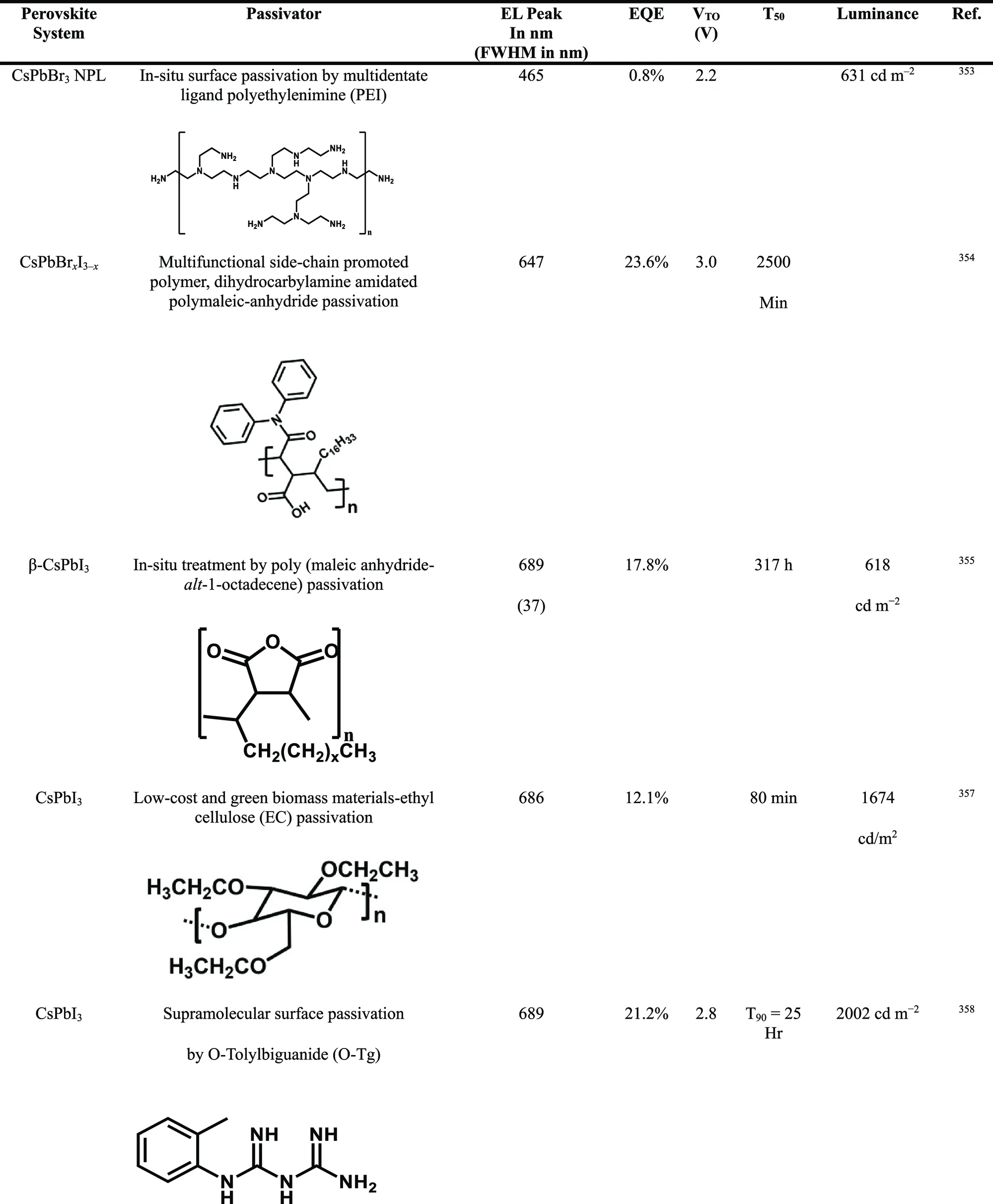
Summary of Polymeric/Supramolecular Surface Passivation Strategies Reported for MHP NC-Based PeLEDs, Highlighting Their Influence on Device Efficiency and Performance Parameters^353,354,355,357,358^

#### Zwitterionic Ligand Passivation

Zwitterionic passivators with simultaneous cationic and anionic ends can bind to the MHP NC surface. For example, the sulfobetaine ligand was used for mixed halide CsPb­(Br/Cl)_3_ to achieve stable EL at 463 nm under increasing bias.^359^ Among all, the zwitterionic ligand, lecithin, has been found to be one of the efficienct passivators, which strongly binds to CsPbI_3_ NCs without inducing surface strain, effectively stabilizing the perovskite phase, enhancing PL and device performance (Figure 17b). This has enabled the fabrication of phase-stable LEDs with EQE of 7.1%.^356^ Table 14 provides an overview of key zwitterionic ligands-based surface passivation strategies employed for MHP NC based PeLED.

**Table 14:**
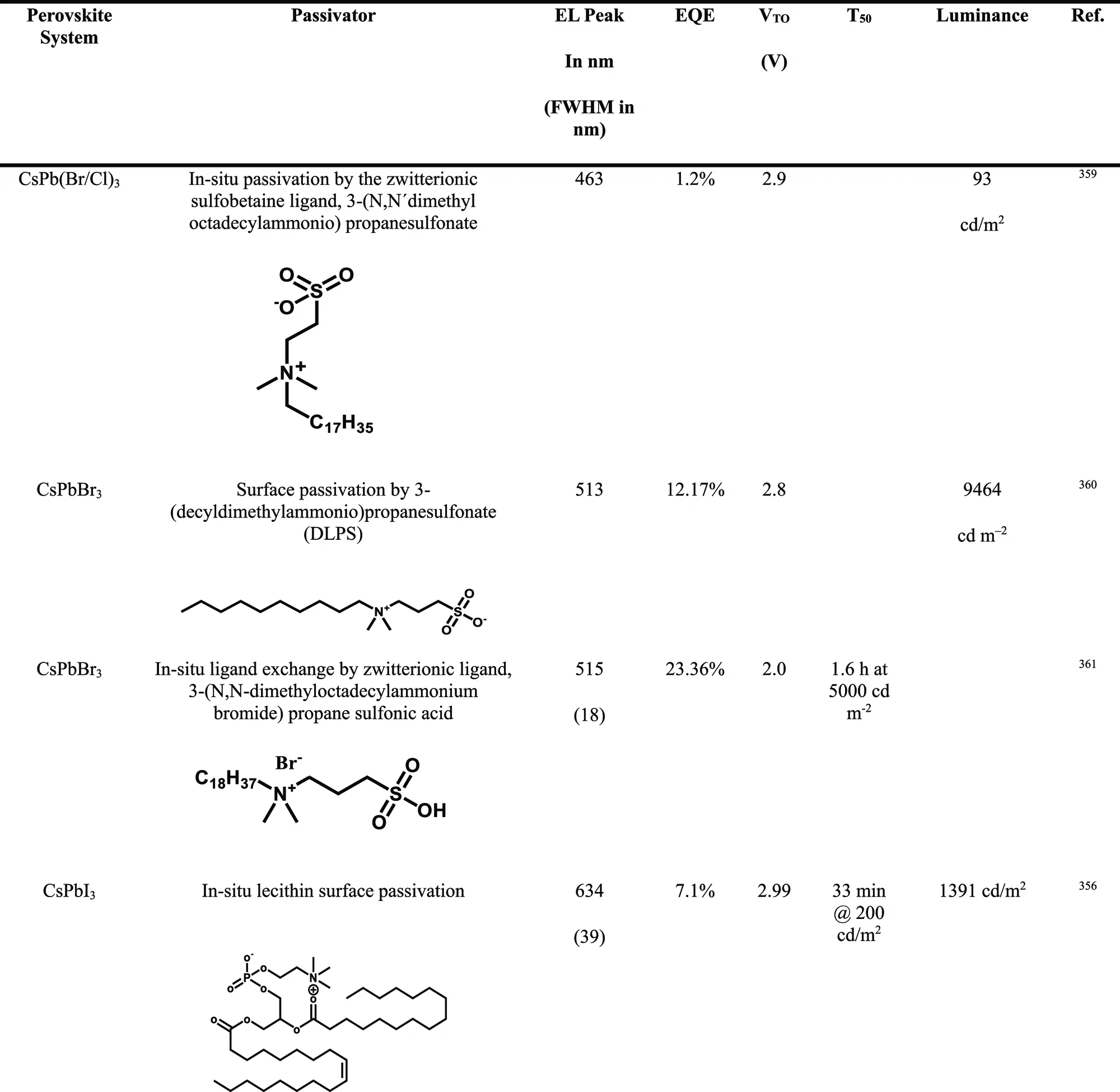
Summary of Zwitterionic Ligands Passivation Strategies Reported for MHP NC-Based Peleds, Highlighting Their Influence on Device Efficiency and Performance Parameters^356,359,360,361^

#### Short Chain Ligand Passivation

To overcome the limitations of the insulating nature and weakly bound long-chain ligands such as oleic acid (OA) and oleylamine (OAm), researchers have adopted various short-chain, strongly anchoring ligands to enhance surface passivation and charge transport in perovskite NCs. For example, *N*-dodecylbenzenesulfonic acid (DBSA) was used to achieve well-ordered CsPbBr_3_ NPL surfaces, which prevented bias-induced redshift of desired EL peak in PeLED device.^362^ The ligands can also be introduced during the synthesis of NCs via an in situ ligand compensation strategy. For example, the combined use of dimethyl butylamine and ethyl bromide leads to the in situ formation of dimethyl ethylammonium bromide, which then binds to perovskite CsPbBr_3_ NCs.^363^ The resultant alkylammonium ions passivate the Cs^+^ vacancies while Br^–^ ions heal surface bromide defects, significantly lowering trap density and enhancing electronic coupling with NC

To combat ion migration, multisite cross-linking ligands, such as α-lipoic acid (LA) has been used.^364^ During the NC purification, LA initially coordinates with Pb^2+^ ions, and upon thermal activation, its disulfide bonds cleave to form a cross-linked poly-LA network (Figure 18a,b). This robust structure passivates undercoordinated sites and stabilizes NC surfaces, delivering LEDs with a record EQE of 25.7% (Figure 18c), peak luminance of 56700 cd/m^2^, and a *T*_50_ lifetime of 1300 h at 100 cd/m^2^. To restrict the nucleation and for achieving stable blue EL in CsPbBr_3_ NPL, butylamine, myristic acid, and ammonium bromide were jointly used, followed by phenylethylammonium bromide passivation, achieving a notable EQE of 2% in blue PELED (Figure 18d).^365^ Additionally, short-chain ligands like octylphosphonic acid^367^ and aromatic 2-naphthalenesulfonic acid^368^ have also been employed due to their strong surface binding capabilities. More recently, iodotrimethylsilane (TMIS), a reactive and nonpolar ligand that generates HI in situ by reacting with oleate ligands, was used as passivator (Figure 18e).^366^ This etches the NC’s surface and enables iodide passivation, leading to tightly bound surface ligands. This enabled the fabrication of LEDs with a remarkable ∼ 23% EQE for red-emitting CsPbI_3_ NCs and EL at 656 nm (Figure 18f,g). In Table 15, we have highlighted various short chain ligand passivation strategies from recent literature reports.

**Figure 18:**
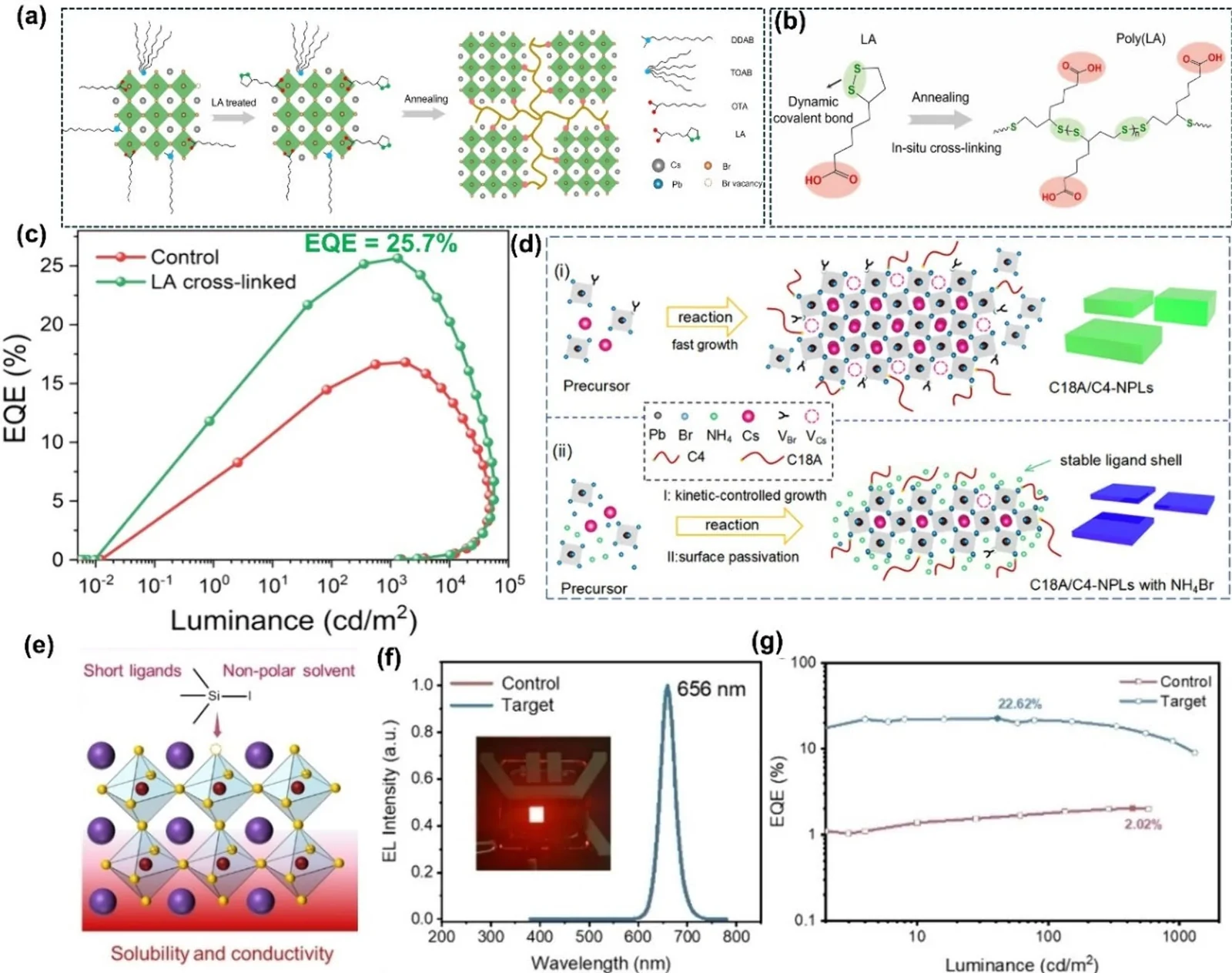
(a) Schematic illustration of the α-lipoic acid (LA)-treatment for CsPbBr_3_ QDs (b) Schematic illustration of the cross-linking process on the QD surface (c) EQE as a function of luminance for the QD based LED. Panels (a)–(c) reproduced with permission from ref 364. Copyright 2025 Wiley-VCH GmbH. (d) Schematic presentation of the formation of (i) C18A/C4 and (ii) NH_4_^+^/C18A/C4. Reproduced with permission from ref 365. Copyright 2022 American Chemical Society. (e) Schematic presentation for new strategy employing the liquid and chemically reactive agent iodotrimethylsilane (TMIS) to enable conductive and well-passivated NCs. (f) EL spectra of control and TMIS-treated CsPbI_3_ LEDs (inset: the photo of TMIS-treated operating CsPbI_3_ LEDs at 3 V). (g) EQE vs Luminance curves of control and TMIS-treated CsPbI_3_ LEDs. Panels (e)–(g) reproduced with permission from ref 366. Copyright 2023 Wiley-VCH GmbH.

**Table 15:**
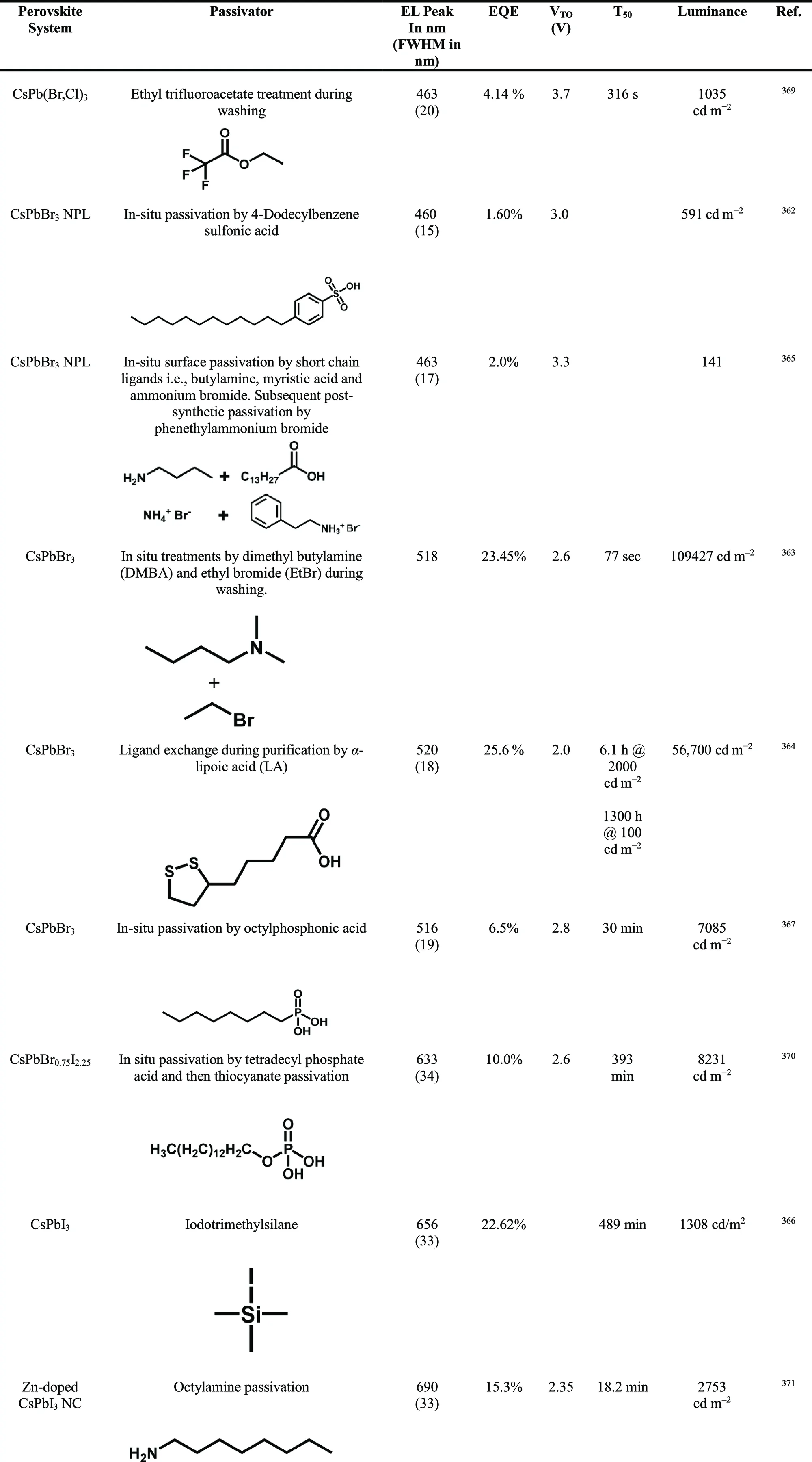
Summary of Short Chain Ligands Passivation Strategies Reported For MHP NC-Based PeLEDs, Highlighting Their Influence on Device Efficiency and Performance Parameters^362,363,364,365,366,367,369,370,371^

#### Conjugated Ligand Passivation

Conjugated ligands, containing π-conjugated systems, play a crucial role in advancing PeLED performance by offering dual functionality: robust surface passivation and enhanced charge transport. The semiconducting ligands like triphenyl­(9-phenyl-9*H*-carbazol-3-yl) phosphonium sulfate (TPPcarz_2_SO_4_) and bromide analogue (TPPcarzBr) where the phosphonium-based cation promotes efficient charge transport, producing PeLEDs with a EQE of 4.15% (Figure 19a).^372^ To extend dual passivation approach, a simple aromatic compound, benzhydroxamic acid was introduced as a surface modifier, which simultaneously bind with Pb^2+^ and make H-bonding with formamidinium cation, and thus reduces nonradiative recombination, leading to the fabrication of PeLEDs with 24.8% EQE (Figure 19b).^373^ On another front, by leveraging the strong surface anchoring properties of 2-naphthalene sulfonic acid and ammonium hexafluorophosphate through a ligand exchange strategy, Ostwald ripening in CsPbI_3_ NCs can be effectively suppressed (Figure 19c). This has enabled the preparation of high-quality CsPbI_3_ QDs, yielding LEDs with an EQE of 26.4% with EL peaking at 628 nm and a notable *T*_50_ lifetime of 729 min at 1000 cd. m^–2^.^374^ In Table 16, we have summarized different representative examples of conjugating-ligand-based surface passivation strategies.

**Figure 19:**
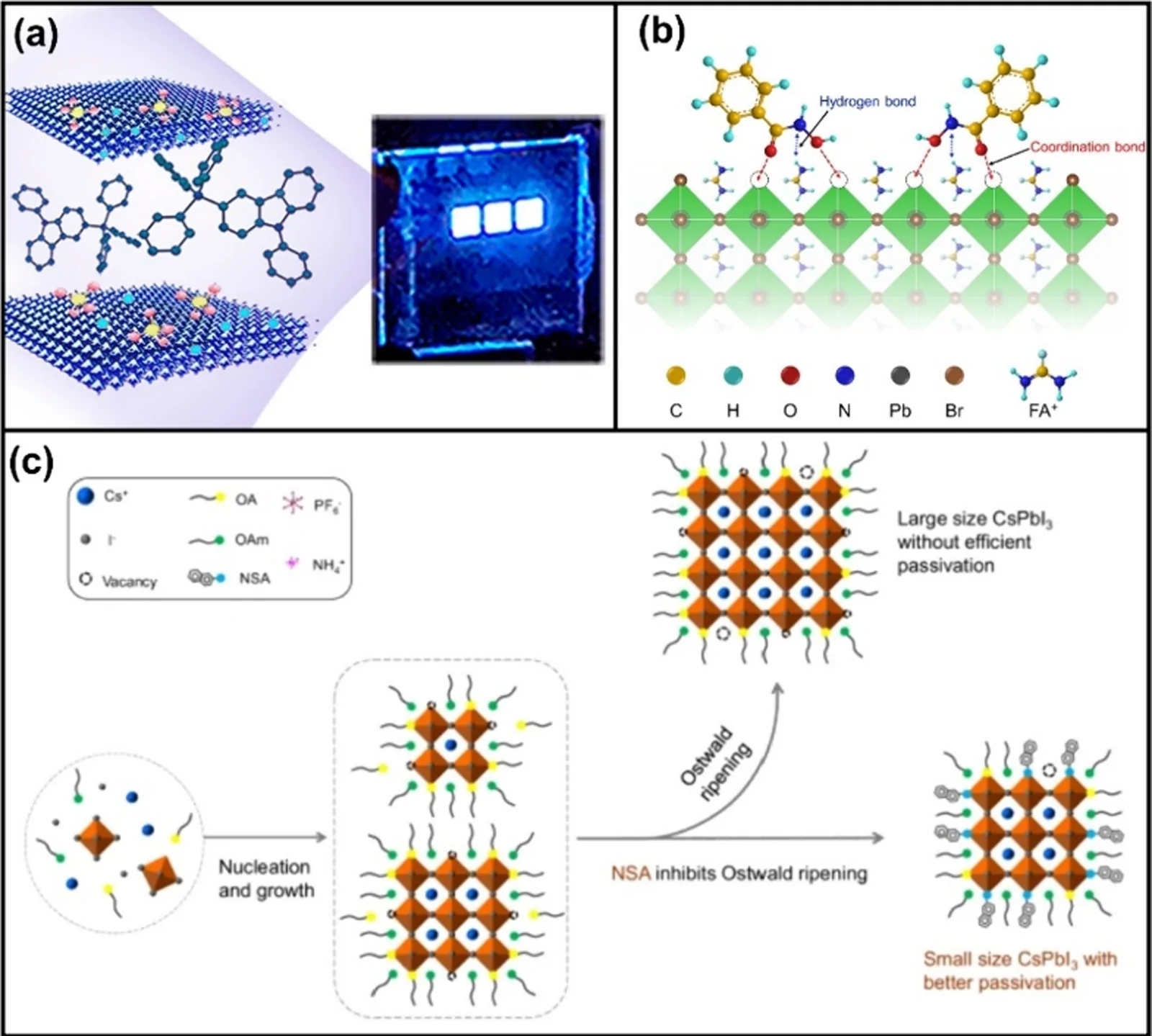
(a) This schematic shows the passivation of CsPbBr_3_ NPL by organic semiconducting ligands triphenyl­[(9-phenyl-9*H*-carbazol-3-yl) phosphonium] sulfate (TPPcarz_2_SO_4_) and bromide (TPPcarzBr) with corresponding blue EL of LED. The figure is reprinted with permission from ref 372. Copyright 2023 American Chemical Society. (b) Schematic illustration of the binding property of benzhydroxamic acid at the surface of the FAPbBr_3_ PeQDs. Reproduced with permission from ref 373. Copyright 2024 American Chemical Society. (c) Schematic diagram of 2-naphthalenesulfonic acid (NSA) ligand passivating CsPbI_3_ QDs and inhibiting Ostwald ripening. Reproduced with permission from ref 374. Available under a CC-BY 4.0. Copyright 2024 The Author(s). Published by Springer Nature.

**Table 16:**
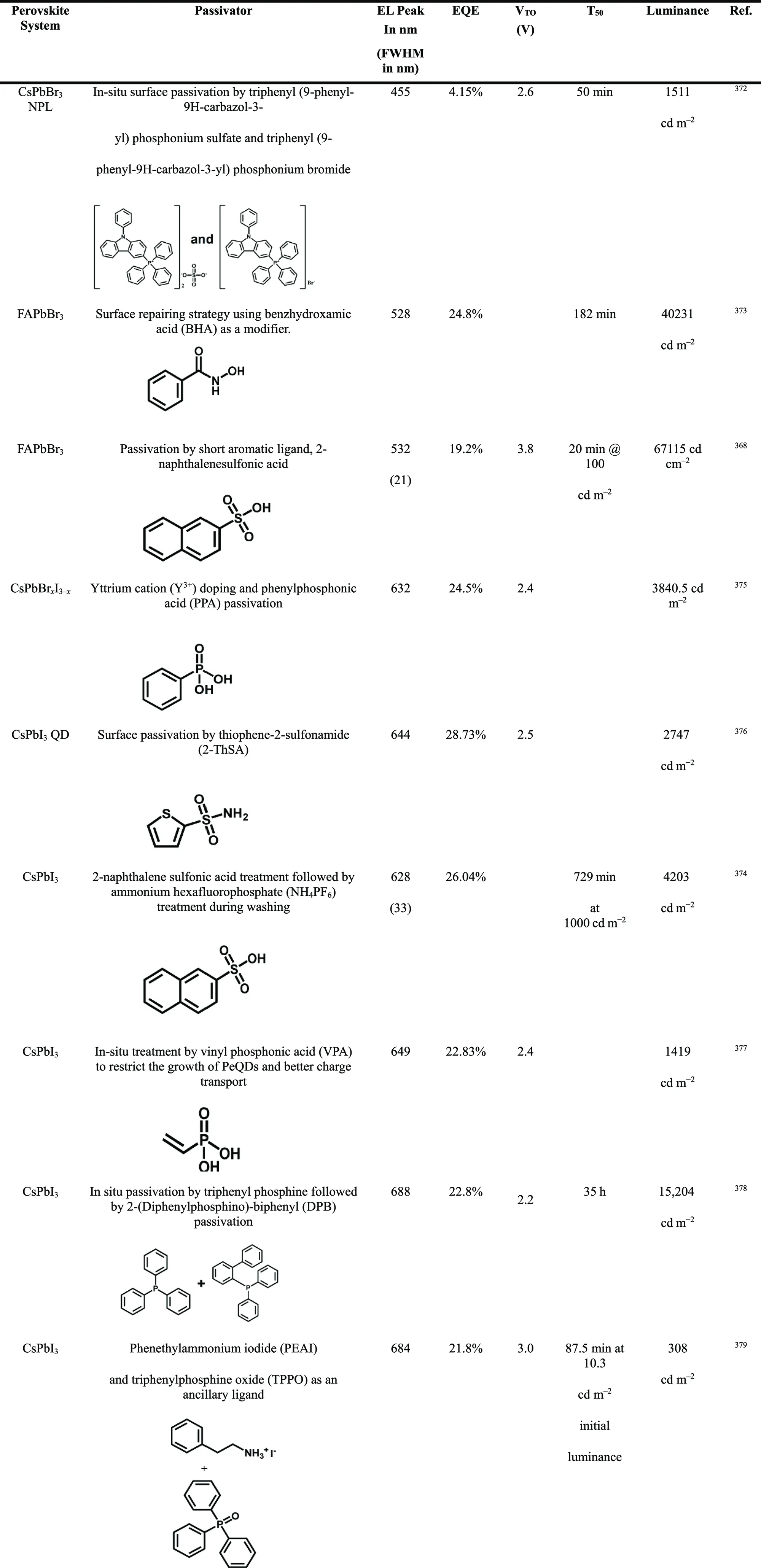
Summary of Conjugating Ligands Passivation Strategies Reported For MHP NC -Based PeLEDs, Highlighting Their Influence On Device Efficiency And Performance Parameters^368,372,373,374,375,376,377,378,379^

#### Branched Chain Ligand Passivation

Branched ligands effectively passivate lead halide perovskite nanocrystals by offering steric hindrance and multidentate surface anchoring. Their bulky architecture ensures dense surface coverage and strong binding, efficiently neutralizing defect sites and suppressing nonradiative recombination. For instance, it was found that the incorporation of strong surface anchoring and branched-chain diisooctylphosphinic acid passivation, followed by hydroiodic acid (HI) etching into a crude perovskite NC solution (later strategy promotes in situ formation of oleylammonium iodide), totally modifies the surface capping ligand environment (Figure 20a). This has enabled the fabrication of CsPbI_3_ PeLEDs of 28.5% EQE with a record *T*_50_ operational lifetime of 30 h at 100 cd m^–2^ initial luminance.^244^ Table 17 provides some representative examples of branched-chain surface passivation strategies adapted in the literature for PeLED.

**Table 17:**
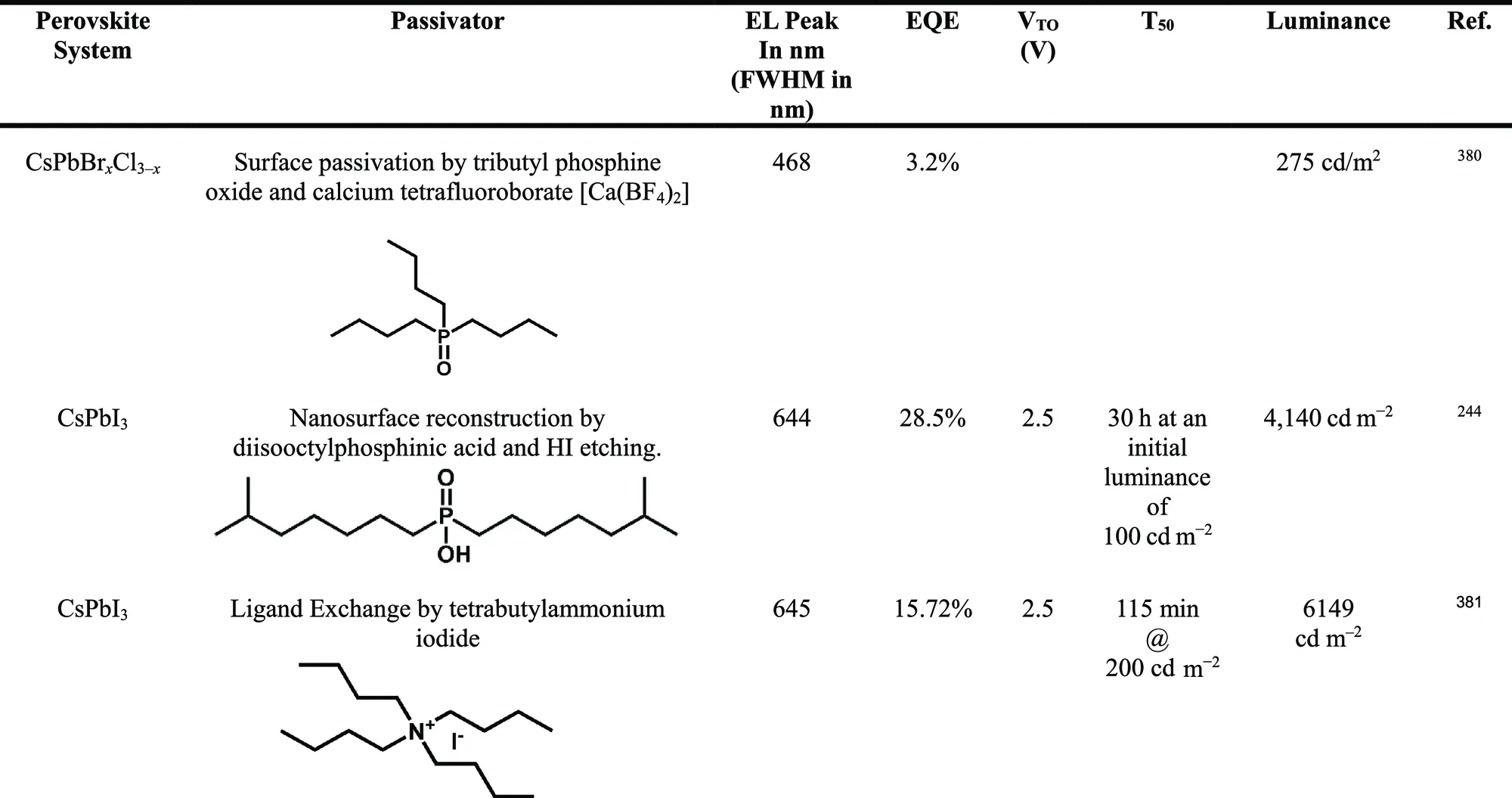
Summary of Branched Chain Ligands Passivation Strategies Reported for MHP NC-Based PeLEDs, Highlighting Their Influence on Device Efficiency and Performance Parameters^244,380,381^

**Figure 20:**
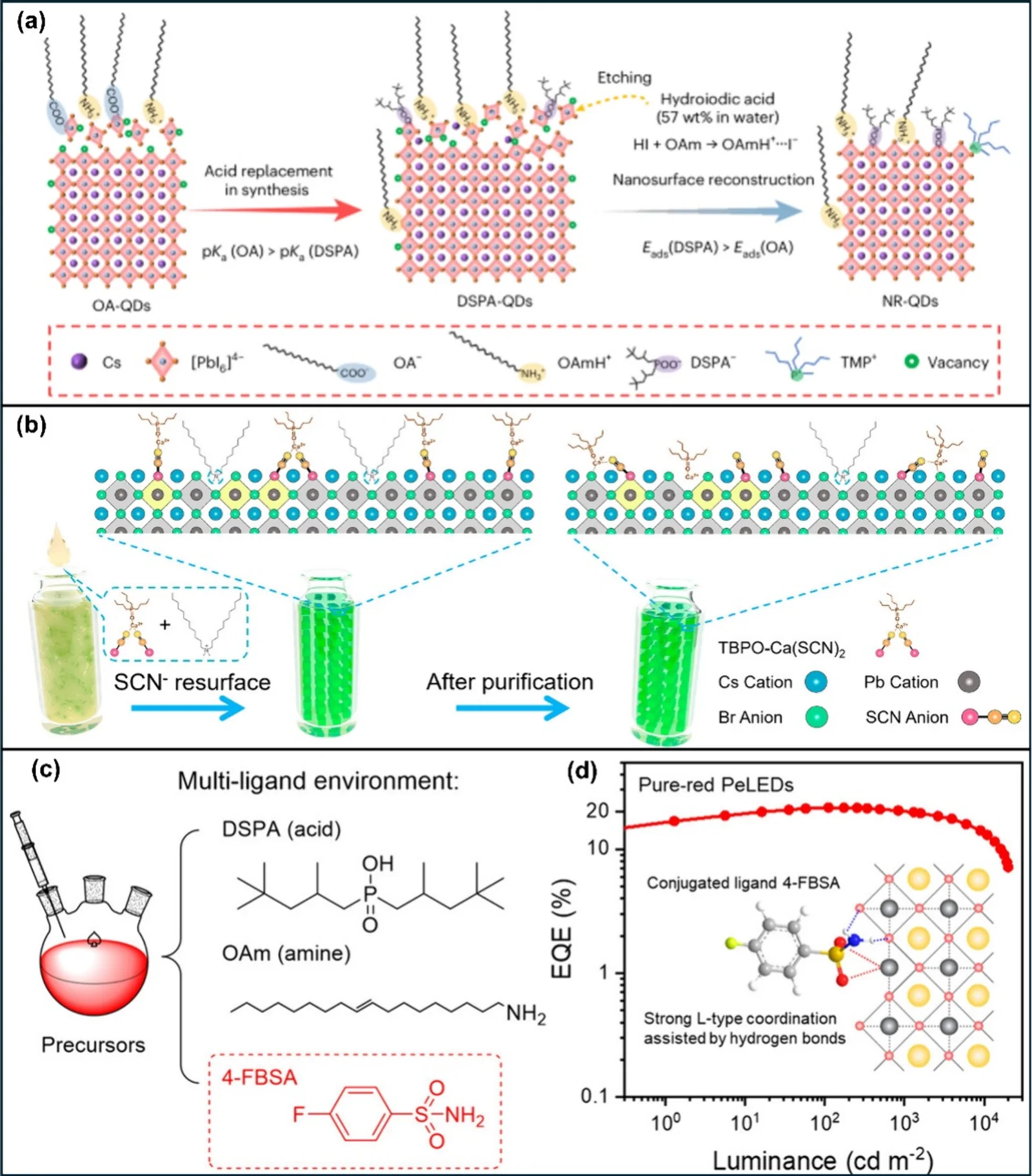
(a) Schematic illustration of the nanosurface reconstruction of the QDs by diisooctylphosphinic acid and HI etching. Reproduced with permission from ref 244. Copyright 2024 The Author(s), under exclusive license to Springer Nature Limited. (b) Schematic diagram of the introduction of thiocyanate ions to modify the surface of CsPbBr_3_ NCs by a TBPO-Ca­(SCN)_2_ toluene solution. Reproduced with permission from ref 383. Copyright 2023 American Chemical Society. (c) Chemical structures of ligands in the precursors. (d) EQE–luminance curves of the 4-FBSA passivated device. The inset schematic illustration represents the interaction between the 4-FBSA and NCs, showing L-type coordination to the CsPb­(Br/I)_3_ NCs between the Pb and −S­(=O)_2_– group, and the hydrogen bond between the −NH_2_ group and the halide. Reproduced with permission from ref 384. Copyright 2024 American Chemical Society.

#### Branched Ligands Passivation Combination with Other Functional Ligands

Branched ligands, when synergistically combined with inorganic salts, organic salts, or conjugated ligands, significantly boost the PeLED performance by reinforcing surface passivation and minimizing defect-mediated losses. Strongly confined CsPbBr_3_ QDs often suffer from halide vacancy-induced trap states due to high surface area to volume ratio. To address this, first an effective HBr treatment followed by branched ligands passivation were applied. The HBr treatment repairs Br-vacancy with removal of excess long-chain carboxylic acids). Subsequent surface passivation with a combination of branched didodecylamine and aromatic phenethylamine ligands resulted in the fabrication of PeLEDs stable EL at 470 nm for CspbBr_3_ QDs with device stability.^382^ Mitigating surface Br-vacancies by external Br^–^ ions could lead to the accumulation of excess Br^–^ ions at grain boundaries, which can accelerate ion migration-induced degradation in PeLEDs, undermining long-term device stability. To avoid the awful effect of excess Br^–^ ions in device, pseudohalide i.e., SCN^–^ surface passivation strategy is adopted. As a pseudohalide source, Ca­(SCN)_2_ salt was employed, but to increase its ionic dissociation in nonpolar solvent, branched tributyl phosphine oxide (TBPO) was utilized. The strong adhesion of SCN^–^ with Pb^2+^ enhances the surface interactions (Figure 20b) with a commendable EQE of 17.2%.^383^

During the colloidal synthesis of perovskite NCs, it is well-known that the oleylamine is easily protonated by oleic acid OA, weakening its ability to passivate the surface as an L-type ligand. To overcome this, 4-fluorobenzenesulfonamide, a small aromatic amine with reduced −NH_2_ basicity, was introduced due to strong P−π conjugation (Figure 20c).^384^ This allowed for more effective, unprotonated L-type surface coordination of the mixed halide CsPb­(Br,I)_3_ NC, enabling the fabrication of mixed-halide PeLEDs with an EQE of 21.8% and luminance of 21590 cd/m^2^ (Figure 20d). Based on the collected reports, Table 18 provides a summary of passivation strategies, where branched-chain ligands with other functional ligands were used as passivators.

**Table 18:**
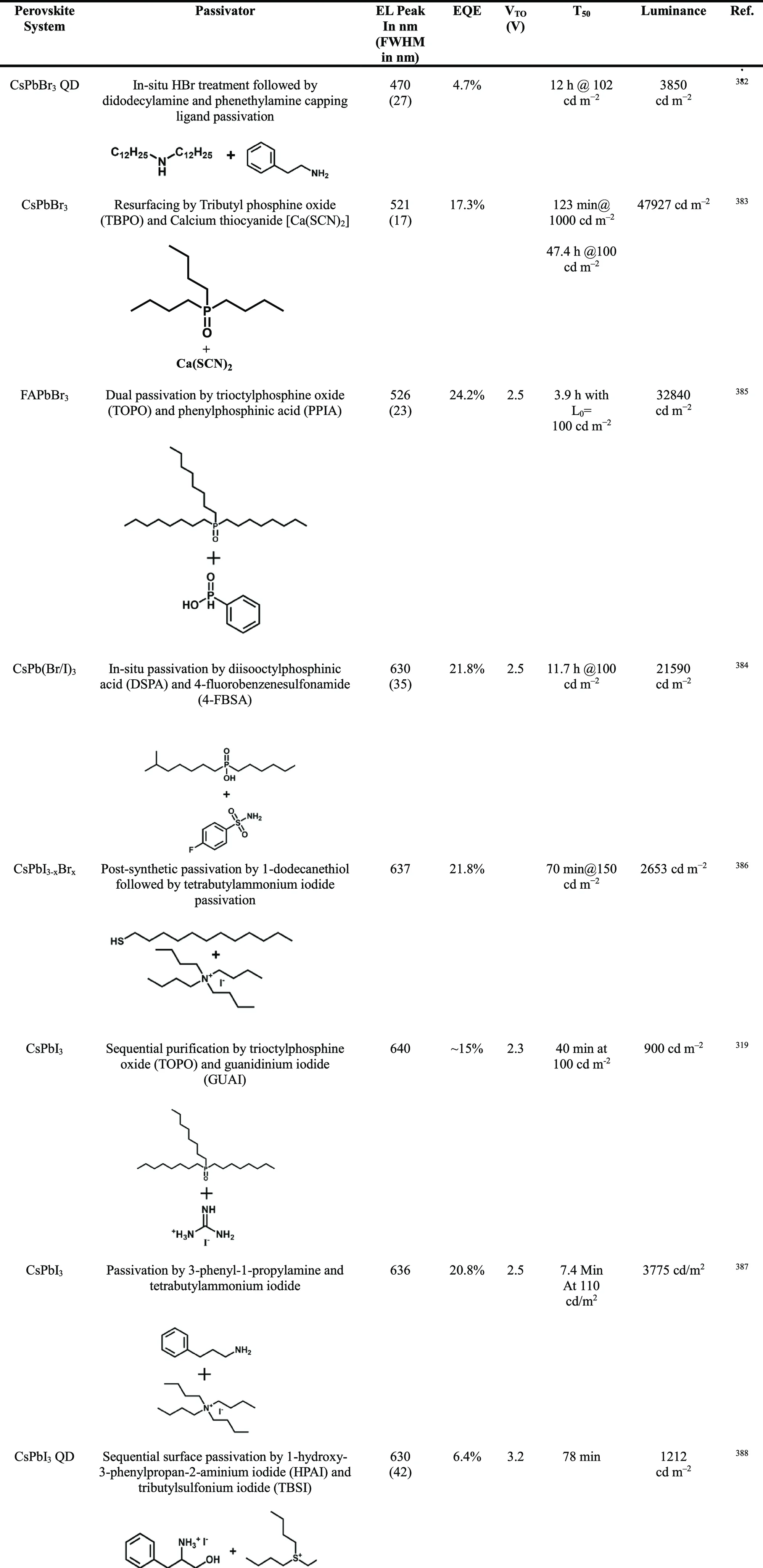
Summary of Branched Chain Ligands Combination with Other Functional Ligands Used For Passivation of MHP NC-For PeLEDs, Highlighting Their Influence On Device Efficiency and Performance Metrics^319,382,383,384,385,386,387,388^

## Summary and Outlook

Although metal halide perovskites exhibit an inherent degree of defect tolerance, defects at surfaces, grain boundaries, and interfaces remain decisive factors, limiting efficiency, stability, and carrier dynamics. This comprehensive review offers a mechanistic perspective on defect formation and passivation in MHPs, along with passivator molecule types, highlighting the fundamental photophysics of defects and how passivation strategies influence the performance of PSCs and PeLEDs. It first discusses the origin of bulk and surface defects in perovskites, including vacancies and undercoordinated ions, and explains how these defects introduce trap states that promote nonradiative recombination, ion migration, and energetic disorder. Subsequently, the review details a wide range of passivation approaches developed to mitigate these defects, focusing on molecular-, ionic-, and ligand-based strategies. For perovskite solar cells, passivation is noted to suppress recombination losses, improve charge extraction, reduce hysteresis, and enhance the operational stability under light and thermal stress. Parallelly, the summary of passivation strategies in PeLEDs highlights their role in improving radiative recombination efficiency, balancing charge injection, and stabilizing emissive phases. Comparative tables throughout the discussion not only summarize different passivator ligand types reported in the literature but also illustrate the effectiveness of defect passivation that leads to significant improvements in PCE, FF, EQE, luminance, and device lifetime across different perovskite compositions. Overall, the review establishes defect passivation as a central strategy linking microscopic defect control to macroscopic performance enhancement in both PSCs and PeLEDs. Despite remarkable progress, challenges persist in achieving efficient PSCs and PeLEDs with long-term stability through passivation.

PSCs have greatly benefited from defect passivation, in terms of improving their PCE and stability. However, the operational stability of PSCs is far behind the commercial solar cells (a few hrs and days vs years). More efforts should be focused on improving the stability through combined passivation and encapsulation. The effective passivators should be rationalized and sorted from the huge library of available materials in the literature. The in situ and postsynthetic film or nanocrystal passivation strategies should be compared. We strongly believe that further in-depth studies are needed to fully understand the complexity and density of defects and the passivation of surface vs bulk defects. It is noted that most strategies passivate surface defects and grain boundaries. Furthermore, it is critical to further understand the ligand–perovskite interactions, as they react with each other and can form different chemical species on the surface of perovskites. More effort should also be devoted to the long-term viability of passivation strategies, as the passivators can degrade over time under moisture, heat, and light. We believe that future studies will also focus on suppressing ion migration-induced hysteresis through effective passivation of defects. It would be interesting to explore which ligand types would be more effective in this regard.

Deeper atomic-level understanding of passivator–perovskite interactions and passivation mechanisms, supported by multiscale simulations and machine-learned potentials, is essential to rationally design passivators that simultaneously passivate defects and preserve charge transport.

To further advance PSCs toward commercialization, passivation strategies should be tested for large-area perovskite solar modules. However, significant stability and efficiency challenges arise during the fabrication of large-area solar cells. Lab-scale devices are fabricated under precisely controlled conditions via spin coating and antisolvent quenching, providing dense, large, and uniform grains with low defect density and pinhole-free films. In contrast, large-area devices fabricated by scalable deposition techniques, such as slot-die coating, blade coating, spray coating, or vapor deposition, often cause several problems, including nonuniform solvent evaporation, thermal gradients, increased defect densities, grain boundaries, and thickness fluctuations. Among these, grain boundaries are the most crucial, leading to halide vacancies, ion migration, and increased nonradiative recombination. Also, larger PSCs suffer from atomic point defects to macroscopic morphological defects. Addressing these defects requires precise, scalable passivation strategies that are first evaluated at the lab scale and then scaled up for larger PSCs, including adding ligands such as fullerene and its derivatives,^125,146–159^ alkyl and aryl amines and ammonium salts,^160–164^ carboxylic acids, esters, carbon quantum dots, graphitic carbon nitride, carbon nanotubes,^193–199^ organic dyes,^200–202^ inorganic oxides and salts,^203–215^ and Lewis acid and base.^74,124,137,148,175,190,210^ These ligands provide chemical robustness, solution-processability, uniform thickness, and the ability to heal grain boundaries and pinholes, thereby enhancing the efficiency and stability of PSCs. Also, ligands, as well as the ETL and HTL layers, with matching energy alignments are important for interfacial carrier extraction and transport, in addition to the chemical and thermal stability of each component. Therefore, rational selection of ETL and HTL, together with additive ligands, enables the simultaneous enhancement of PSC efficiency and operational stability during large-scale fabrication.

Along similar lines, PeLEDs have demonstrated significant improvements in EQE and relative stability; however, their long-term viability remains limited by operational instability, efficiency roll-off, and unresolved degradation mechanisms under an electrical bias. After discussing various surface passivation strategies, it is essential to evaluate the current status of EQE and the operational stability of PeLEDs. As highlighted in this review, effective surface passivation significantly mitigates defect states and enhances radiative recombination pathways, enabling EQEs exceeding 30%. However, despite these impressive efficiency gains, operational stability remains a critical challenge, primarily governed by ion migration within the perovskite lattice. In typical PeLEDs, the activation energy for ion migration is below 1 V,^389^ whereas the minimum turn-on voltage required for PeLED device operation is considerably higher. This voltage mismatch accelerates ion migration under the operating conditions, leading to device degradation. Although EQE and operational stability are often treated as independent performance metrics, future surface passivation strategies hold promise for simultaneously addressing both simultaneously. In particular, multifunctional and cross-linkable passivators with multisite coordination capabilities can not only suppress defect states but also increase the activation energy for ion migration with enhanced bonding interactions, thereby improving both efficiency and operational stability. It can simultaneously address mitigating Auger recombination and charge imbalance at high current densities. Therefore, a key perspective for the field is shifting from ex situ performance metrics to operando characterization, where real-time spectroscopic and structural probes can directly observe ligand desorption, ion migration, interfacial reactions, and morphological changes caused by Joule heating during device operation. In parallel, a deeper atomic-level understanding of ligand–perovskite interactions, supported by multiscale simulations and machine-learned potentials, will be essential to rationally design ligands that simultaneously passivate defects, immobilize halides, and preserve charge transport. As summarized in tables, the library of passivator molecules is huge; therefore, the selection of effective passivators requires comparative studies between different passivator molecules under similar experimental conditions. Future studies could focus on combining molecular passivation and polymer encapsulation of devices to achieve long-term stability and efficiency of perovskite optoelectronic devices. Moreover, blue PeLEDs remain a critical bottleneck, necessitating integrated strategies that combine phase control, interfacial engineering, and nanoscale confinement rather than relying solely on defect passivation. Finally, extending robust passivation concepts to lead-free perovskites represents a promising yet underexplored direction. Collectively, these advances point toward a transition from empirical optimization to data-driven, mechanism-guided design, paving the way for efficient, spectrally stable, and commercially viable PeLED technologies. A few key prospects for PeLEDs are briefly outlined here.

- *In-operando Measurements to Probe the Degradation Mechanism*: Extensive efforts are needed toward understanding the degradation mechanisms of PeLEDs under electrical bias by in situ and operando characterization techniques. Advanced spectroscopic and imaging methods such as XPS, FTIR, TRPL, GIWAXS, and electroluminescence mapping can provide real-time insights into ligand loss, interfacial reactions, and ion migration. In parallel, infrared thermal imaging combined with thermal simulations may be fruitful for elucidating heat generation and dissipation pathways that affect stability.
- *Poor Operational Lifetime* (*T*_50_): Despite record-high EQEs, PeLEDs still suffer from noticeable operational lifetimes (*T*_50_), far behind QDLED benchmarks (>1000000 h vs ∼3220 h at 100 cd m^–2^). Future strategies should prioritize extending benchmark operational lifetimes, rather than focusing solely on achieving high PLQY.
- *Atomic-Level Ligand–Nanocrystal Interactions*: It is essential to elucidate how ligands interact with perovskites, govern their stability, and optoelectronic properties at the atomic level. Greater involvement from theoretical researchers, particularly through dedicated *ab initio* computational studies, is needed to address these fundamental mechanisms.
- *Efficiency Roll-off at Higher Current Density*: Decrease of EQE at high bias conditions, referred to as efficiency roll-off, occurs due to enhanced Auger recombination, charge-injection imbalance, and Joule heating. As the library of passivators is large, a mixed passivation approach, such as the simultaneous utilization of inorganic ligands and short, π-conjugated ligands, may be an alternative solution to tackle the aforementioned problems.
- *Employing New Multifunctional Ligands*: Although many passivation approaches remain largely empirical, rationally designed multifunctional ligands, i.e., chelating motifs with carefully tuned conjugation, can simultaneously enhance color purity, operational stability, and charge balance. Researchers have been continuously looking for new passivator types. Continued efforts of synthetic chemists and device engineers will be needed for developing a new set of next-generation, reliable, multifunctional ligands.
- *Efforts toward Blue PeLEDs*: Despite the substantial progress of green and red PeLEDs in terms of their EQE and stability, their blue counterparts are currently lagging with low EQEs. This performance gap is largely attributed to the defect-intolerant nature of chloride perovskites and instability of mixed bromide-chloride compositions, and the tendency of their wider bandgap to induce charge accumulation at interfaces. Therefore, improving blue PeLEDs requires more than simple defect passivation. A synergistic approach, guided by theory, interfacial engineering, and the use of multifunctional passivators is currently essential to bridge this gap.
- *Lead-free LEDs through Surface Passivation*: Despite significant progress in lead-based PeLEDs, only a few studies have been dedicated to lead-free systems, mainly due to their instability in the case of tin perovskites and low PLQY in the case of double perovskite systems. Nevertheless, several low-dimensional lead-free systems, such as CsCu_2_Br_3_, Cs_3_Cu_2_Br_5_, and Cs_2_ZrCl_6,_ etc., have attracted considerable attention owing to their self-trapped exciton (STE) emission and high PLQY, and these systems show promise for down-conversion LEDs. Further studies could be focused on the passivation of tin perovskite LEDs to improve their stability, analogous to the passivation strategies developed for lead-based perovskites.

## ○

Institute of Physics, University of Rostock, Albert-Einstein-Straße 23, 18059 Rostock, Germany
